# Supplementary material for: DFT Investigation of Hydrogen Atom Abstraction from NHC-Boranes by Methyl, Ethyl and Cyanomethyl Radicals—Composition and Correlation Analysis of Kinetic Barriers
Source: Molecules. 2020 Oct 1;25(19):4509. doi: 10.3390/molecules25194509 (PMC7582687; doi:10.3390/molecules25194509)
Supplement: Supplementary file 1 [file molecules-25-04509-s001.pdf]

Supplementary Materials

DFT investigation of hydrogen atom abstraction from NHC-boranes by methyl, ethyl, and cyanomethyl radicals: Composition and correlation analysis of kinetic barriers

Hong-jie Qu, Lang Yuan, Cai-xin Jia, Hai-tao Yu, Hui Xu**

[1] Key Laboratory of Functional Inorganic Material Chemistry (Ministry of Education) and School of Chemistry and Materials Science, Heilongjiang University, Harbin 150080, P. R. China; qhjsxm@163.com (H.-j.Q.); 2020007@hlju.edu.cn (L.Y.); jiacx@hlju.edu.cn (C.-x.J.);

[2] College of Science, Heilongjiang Bayi Agricultural University, Daqing, 163319, P. R. China

[*] Correspondence: yuhaitao@hlju.edu.cn (H.-t.Y.); hxu@hlju.edu.cn (H.X.); Tel.: +86-451-86608616

List of Contents in Supplementary Materials

A preliminary mathematical physical treatment for the origin of the linear three-variable model used in manuscript······6

Figure S1. Percentages of thermal contribution and intrinsic barrier in activation barrier computed at the M06-2X level. ······8

Figure S2. Percentage stacked bar chart of thermal contribution and intrinsic barrier as the components of activation barrier for the HAA reactions of NHC-boranes by $^{•}CH_2CN$ (a), Me$^{•}$ (b), and Et$^{•}$ (c) at the B3LYP level. ······9

Figure S3. Percentage stacked bar chart of thermal contribution and intrinsic barrier as the components of activation barrier for the HAA reactions of NHC-boranes by $^{•}CH_2CN$ (a), Me$^{•}$ (b), and Et$^{•}$ (c) at the M06-2X level. ······10

Figure S4. Computed activation Gibbs thermal corrections (a), thermal contributions (b), reaction energies (c), and free energy barriers (d), activation barriers (e), and intrinsic barriers (f) at the M06-2X level. ······11

Figure S5. Vertical bar plots of thermal contributions, intrinsic barriers, and activation barriers for the HAA reactions of NHC-boranes by $^{•}CH_2CN$ (a), Me$^{•}$ (b), and Et$^{•}$ (c) at the B3LYP level. ······12

Figure S6. Vertical bar plots of thermal contributions, intrinsic barriers, and activation barriers for the HAA reactions of NHC-boranes by $^{•}CH_2CN$ (a), Me$^{•}$ (b), and Et$^{•}$ (c) at the M06-2X level. ······13

Figure S7. Dependence of activation barrier on intrinsic barrier and reaction energy using the suggested three-variable linear model at the M06-2X level. The R-squared values in red and the plane in blue represent the linear GOF of the computed data points (in red) and the image of the fitted equation using the suggested three-variable model. The R-squared values in olive and violet refer to the linear fitting CODs of the projection points of the computed data points on the corresponding coordinate planes. ······14

Figure S8. Vertical bar plots of activation Gibbs thermal corrections, thermal contributions, activation ZPVE corrections, intrinsic barriers, and free energy barriers for the HAA reactions of NHC-boranes by $^{•}CH_2CN$ (a), Me$^{•}$ (b), and Et$^{•}$ (c) at the B3LYP level. ······15

Figure S9. Vertical bar plots of activation Gibbs thermal corrections, thermal contributions, activation ZPVE corrections, intrinsic barriers, and free energy barriers for the HAA reactions of NHC-boranes by $^{•}CH_2CN$ (a), Me$^{•}$ (b), and Et$^{•}$ (c) at the M06-2X level. ······16

Figure S10. Percentage stacked bar chart of activation ZPVE corrections, thermal contributions, intrinsic barriers, and activation Gibbs thermal corrections as the components of free energy barriers for the HAA reactions of NHC-boranes by $^{•}CH_2CN$ (a), Me$^{•}$ (b), and Et$^{•}$ (c) at the B3LYP level. ······17

Figure S11. Percentage stacked bar chart of activation ZPVE corrections, thermal contributions, intrinsic barriers, and activation Gibbs thermal corrections as the components of free energy barriers for the HAA reactions of NHC-boranes by $^{\bullet}CH_2CN$ (a), $Me^{\bullet}$ (b), and $Et^{\bullet}$ (c) at the M06-2X level. ·····18
Figure S12. Percentage stacked bar chart of activation ZPVE corrections, activation barriers, and activation Gibbs thermal corrections as the components of free energy barriers for the HAA reactions of NHC-boranes by $^{\bullet}CH_2CN$ (a), $Me^{\bullet}$ (b), and $Et^{\bullet}$ (c) at the B3LYP level. ·····19
Figure S13. Percentage stacked bar chart of activation ZPVE corrections, activation barriers, and activation Gibbs thermal corrections as the components of free energy barriers for the HAA reactions of NHC-boranes by $^{\bullet}CH_2CN$ (a), $Me^{\bullet}$ (b), and $Et^{\bullet}$ (c) at the M06-2X level. ·····20
Figure S14. Bar chart of activation barriers, free energy barriers, and activation Gibbs thermal corrections for the HAA reactions of NHC-boranes by $^{\bullet}CH_2CN$ (a), $Me^{\bullet}$ (b), and $Et^{\bullet}$ (c) at the B3LYP level. ·····21
Figure S15. Bar chart of activation barriers, free energy barriers, and activation Gibbs thermal corrections for the HAA reactions of NHC-boranes by $^{\bullet}CH_2CN$ (a), $Me^{\bullet}$ (b), and $Et^{\bullet}$ (c) at the M06-2X level. ·····22
Figure S16. Linear correlation analysis of free energy barrier with intrinsic and activation barriers (a) and with activation Gibbs thermal corrections and thermal contributions (b) at the M06-2X level. ·····23
Figure S17. Dependence of free energy barrier on reaction Gibbs free energy and intrinsic barrier (a)/activation barrier (b) using the suggested three-variable linear model at the M06-2X level. The R-squared values in red and the plane in blue represent the linear GOF of the computed data points (in red) and the image of the fitted equation using the suggested three-variable linear model. The R-squared values in olive and violet refer to the linear fitting CODs of the projection points of the computed data points on the corresponding coordinate planes. ·····24
Figure S18. Linear correlation analysis of BDEs with reaction energies and thermal contributions at the M06-2X level. ·····25
Table S1. Total electronic energies (TE, hartree), zero-point vibrational energies (ZPVE, hartree/particle), Gibbs free energies (G, hartree), enthalpies (H, hartree), thermal corrections to Gibbs free energy (G_{corr}, hartree), and thermal corrections to enthalpy (H_{corr}, hartree) of the structures of NHC-boranes, NHC-boryl radicals, attacking radical $R^{\bullet}$ and RH optimized at the B3LYP/6-311++G(d,p) level of theory in BTF at 376 K. ·····26
Table S2. Total electronic energies (TE, hartree), zero-point vibrational energies (ZPVE, hartree/particle), Gibbs free energies (G, hartree), enthalpies (H, hartree), thermal corrections to Gibbs free energy (G_{corr}, hartree), and thermal corrections to enthalpy (H_{corr}, hartree) of the structures of NHC-boranes, NHC-boryl radicals, attacking radical $R^{\bullet}$ and RH optimized at the M06-2X/6-311++G(d,p) level of theory

in BTF at 376 K. ··27
Table S3. Total electronic energies (TE, hartree), zero-point vibrational energies (ZPVE, hartree/particle), Gibbs free energies (G, hartree), enthalpies (H, hartree), thermal corrections to Gibbs free energy (G_{corr}, hartree), and thermal corrections to enthalpy (H_{corr}, hartree) of the transition states (**TS**) of the H-atom abstraction reactions of NHC-boranes by Me$^{•}$ optimized at the B3LYP/6-311++G(d,p) and M06-2X/6-311++G(d,p) levels of theory in BTF at 376 K. ·······························28
Table S4. Total electronic energies (TE, hartree), zero-point vibrational energies (ZPVE, hartree/particle), Gibbs free energies (G, hartree), enthalpies (H, hartree), thermal corrections to Gibbs free energy (G_{corr}, hartree), and thermal corrections to enthalpy (H_{corr}, hartree) of the transition states (**TS**) of the H-atom abstraction reactions of NHC-boranes by Et$^{•}$ optimized at the B3LYP/6-311++G(d,p) and M06-2X/6-311++G(d,p) levels of theory in BTF at 376 K. ·······························29
Table S5. Total electronic energies (TE, hartree), zero-point vibrational energies (ZPVE, hartree/particle), Gibbs free energies (G, hartree), enthalpies (H, hartree), thermal corrections to Gibbs free energy (G_{corr}, hartree), and thermal corrections to enthalpy (H_{corr}, hartree) of the transition states (**TS**) of the H-atom abstraction reactions of NHC-boranes by $^{•}CH_2CN$ optimized at the B3LYP/6-311++G(d,p) and M06-2X/6-311++G(d,p) levels of theory in BTF at 376 K. ·······························30
Table S6. Activation barrier ($\Delta E^{\neq}$), activation zero-point vibrational energy correction ($\Delta E^{\neq}_{ZPVE}$), intrinsic barrier ($\Delta E^{\neq}_{0}$), thermal contribution ($\Delta E^{\neq}_{therm}$), activation Gibbs free energy correction ($\Delta G^{\neq}_{corr}$), and Gibbs free energy barrier ($\Delta G^{\neq}$) of the H-atom abstraction reactions of NHC-boranes by Me$^{•}$, Et$^{•}$, and $^{•}CH_2CN$ computed at the B3LYP/6-311++G(d,p) level of theory in BTF at 376 K. All units are in kcal mol^{-1}. ··31
Table S7. Activation barrier ($\Delta E^{\neq}$), activation zero-point vibrational energy correction ($\Delta E^{\neq}_{ZPVE}$), intrinsic barrier ($\Delta E^{\neq}_{0}$), thermal contribution ($\Delta E^{\neq}_{therm}$), activation Gibbs free energy correction ($\Delta G^{\neq}_{corr}$), and Gibbs free energy barrier ($\Delta G^{\neq}$) of the H-atom abstraction reactions of NHC-boranes by Me$^{•}$, Et$^{•}$, and $^{•}CH_2CN$ computed at the M06-2X/6-311++G(d,p) level of theory in BTF at 376 K. All units are in kcal mol^{-1}. ··········32
Table S8. Spin densities ($\rho_{s,B}$, *e*) on the central B atoms, global nucleophilicity (N, eV) and electrophilicity indices (ω, eV), and local nucleophilicity (N_B, eV) and electrophilicity indices (ω_B, eV) of the NHC-boranes and NHC-boryl radicals computed at the mPWPW91/6-311+G(d,p) level of theory in BTF. ·····························33
Table S9. CODs of the linear correlation of reaction energy ($\Delta_r E$) and thermal contribution ($\Delta E^{\neq}_{therm}$) with the spin density distributions of the HAA product NHC-boryl radicals. The basis set is 6-311++G(d,p). ···································34
Table S10. CODs of the linear correlation of the reaction Gibbs free energies ($\Delta_r G$) with the free energy barriers ($\Delta G^{\neq}$) for the HAA reactions of NHC-boranes by different

radicals. The basis set is 6-311++G(d,p). ······34
Table S11. Fitted equations and CODs using the linear three-variable model at the M06-2X/6-311++G(d,p) level of theory. ······34
Table S12. Computed orbital interaction energies (kcal mol^{-1}) in the HAA transition states at the B3LYP/6-311++G(d,p) level of theory. ······35
Table S13. CODs of the linear correlation fittings of barriers with the global nucleophilicity/electrophilicity indices and charges. ······36
Table S14. CODs of the linear correlation fittings of the B3LYP- and M06-2X-computed BDEs and BDHs with the spin densities of the center B atoms of the product NHC-boryl radicals. ······37
Relationship between the BEDs and HAA reaction energies for a series NHC-boranes······38
Table S15. List of Cartesian coordinates of located stationary points at the B3LYP/6-311++G(d,p) and M06-2X/6-311++G(d,p) levels of theory in BTF at 376 K. ······39

A preliminary mathematical physical treatment for the origin of the *linear three-variable model* used in manuscript

The improved Marcus equation (*J. Am. Chem. Soc. 2008, 130, 15038*), a nonlinear expression of the three variables $\Delta E^{\neq}$, $\Delta E_0^{\neq}$, and $\Delta_r E$, can be written as

$$\Delta E^{\neq} = \Delta E_0^{\neq} + \frac{1}{2}\Delta_r E + \frac{(\Delta_r E)^2}{16\Delta E_0^{\neq}} \quad \text{(R1)}$$

We can make the following formula derivation

$$\begin{aligned}\Delta E^{\neq} &= \Delta E_0^{\neq} + \frac{1}{2}\Delta_r E + \frac{(\Delta_r E)^2}{16\Delta E_0^{\neq}} \\ &= \Delta E_0^{\neq} + \frac{8\Delta E_0^{\neq}\Delta_r E + (\Delta_r E)^2}{16\Delta E_0^{\neq}} \\ &= \Delta E_0^{\neq} + \frac{\Delta_r E(8\Delta E_0^{\neq} + \Delta_r E)}{16\Delta E_0^{\neq}}\end{aligned}$$

When $8\Delta E_0^{\neq} \gg |\Delta_r E|$, which is very easy to satisfy for most of reactions, the equation can be directly simplified into a linear relationship, that is,

$$\Delta E^{\neq} \approx \Delta E_0^{\neq} + \frac{1}{2}\Delta_r E \quad \text{(R2)}$$

The simple Marcus-Theory-Type Model equation derived from the Marcus theory and the Proton-Coupled Electron Transfer (PCET) model (*J. Phys. Chem. Lett. 2011, 2, 1481*) is

$$\Delta G^{\neq} = \frac{(\lambda + \Delta_r G)^2}{4\lambda} \quad \text{(R3)}$$

where λ is the Marcus intrinsic barrier. We can make the following transformation

$$\begin{aligned}\Delta G^{\neq} &= \frac{(\lambda + \Delta_r G)^2}{4\lambda} = \frac{\lambda^2 + 2\lambda\Delta_r G + (\Delta_r G)^2}{4\lambda} \\ &= \frac{1}{4}\lambda + \frac{\Delta_r G(2\lambda + \Delta_r G)}{4\lambda}\end{aligned}$$

Under a low reaction driving force ($\Delta_r G$), *i.e.*, $2\lambda \gg |\Delta_r G|$, it can be simplified into

$$\Delta G^{\neq} \approx \frac{1}{4}\lambda + \frac{1}{2}\Delta_r G \quad \text{(R4)}$$

The equations (R4) and (R2) exhibit linear relationships among $\Delta E^{\neq}$, $\Delta E_0^{\neq}$, and $\Delta_r E$ and among $\Delta G^{\neq}$, λ, and $\Delta_r G$, respectively. The two equations can be considered as the mathematical foundation of the *linear three-variable model*, as shown in equations

$$\Delta E^{\neq} = a\Delta E_0^{\neq} + b\Delta_r E + c \quad \text{(R5)}$$

and

$$\Delta G^{\neq} = d\lambda + d\Delta_r G + f \quad \text{(R6)}$$

where the constants a, b, c, d, e, and f can be determined by fitting the two equations

For a type of reaction, if $\Delta E_0^{\neq}$ and 1/4λ vary very slightly or $\Delta E_0^{\neq}$ and λ are approximately linear to $\Delta_r E$ and $\Delta_r G$, respectively, equations (R4) and (R2) can be further simplified as

$$\Delta E^{\neq} = \alpha + \beta \Delta_r E \quad \text{(R7)}$$

and

$$\Delta G^{\neq} = \gamma + \delta \Delta_r G \quad \text{(R8)}$$

where α, β, γ, and δ are constants and can be determined by fitting the two equations. The equations (R7) and (R8) exhibit as *linear bivariable model*, which can be used to describe the relationships between reaction barrier and thermodynamic driving force. Also, the two equations are similar in expression to the well-known Linear Free Energy Relationships (LFERs) at 0 K and a given experimental temperature, although the former come from the Marcus theory.

However, not all types of reactions follow the simplest LFERs like bimolecular addition reactions, especially the atom- or group-abstraction reactions (*J. Am. Chem. Soc. 2019, 141, 15183*). Relative to the equations (R2) and (R4), the disturbance coming from the effect of intrinsic barrier and Marcus intrinsic barrier should be responsible for the inapplicability of the equations (R7) and (R8) in describing H-atom abstraction reactions. If the conditions, $8\Delta E_0^{\neq} \gg |\Delta_r E|$ and $2\lambda \gg |\Delta_r G|$, can be approximately satisfied and the perturbation of intrinsic barrier to reaction driving force is nonlinear, the *linear three-variable models* (R5) and (R6) should be more suitable for descripting the correlation of kinetic barriers and thermodynamic driving force. In the discussion in text, we used intrinsic barrier as a substitute for the Marcus intrinsic barrier in (R6) to consider the applicability of *models*, but not precise mathematical and physical description, because the former has a clearer physical meaning. More detailed mathematical processing is still in progress, and more types of reactions will be incorporated into further investigations to evaluate the applicability of the *model.*

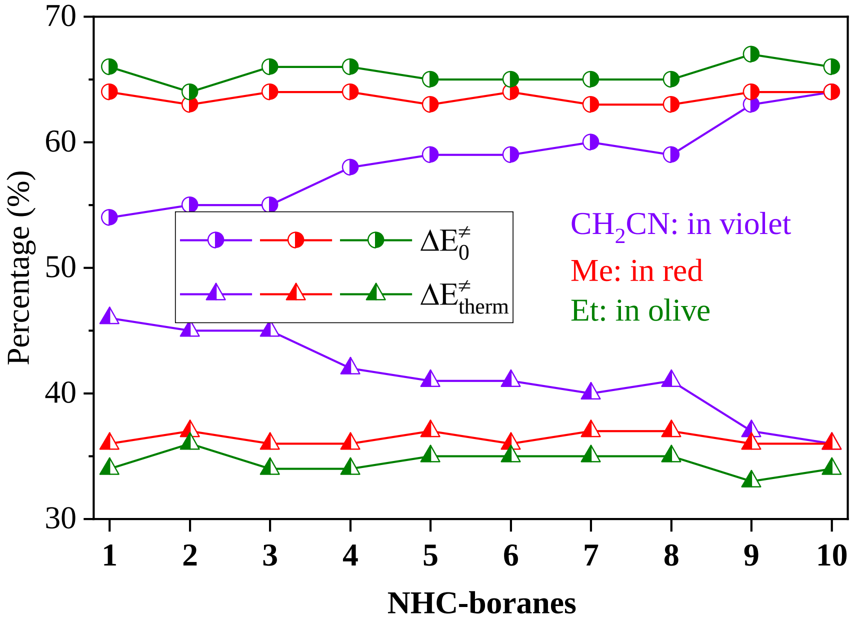

Figure S1. Percentages of thermal contribution and intrinsic barrier in activation barrier computed at the M06-2X level.

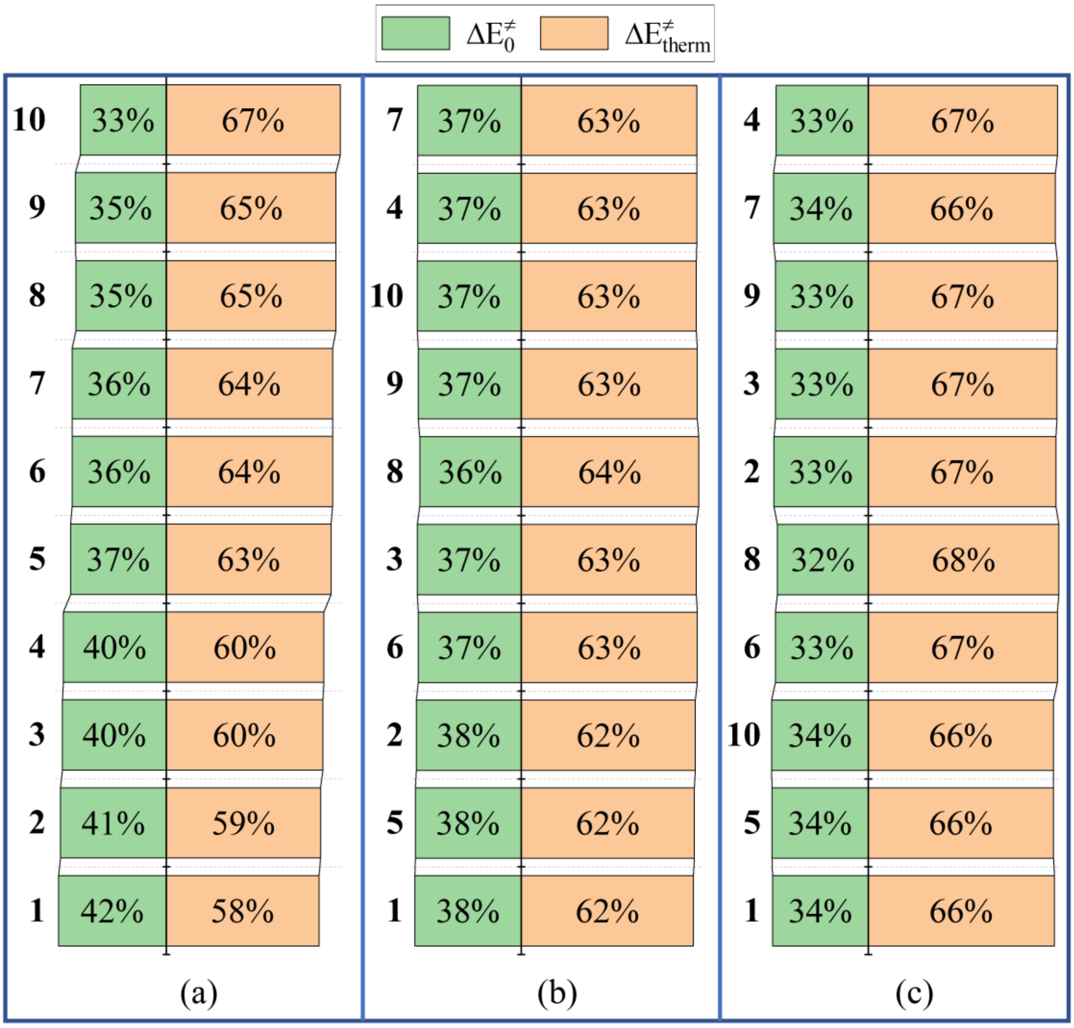

Figure S2. Percentage stacked bar chart of thermal contribution and intrinsic barrier as the components of activation barrier for the HAA reactions of NHC-boranes by $^{\bullet}CH_2CN$ (a), $Me^{\bullet}$ (b), and $Et^{\bullet}$ (c) at the B3LYP level.

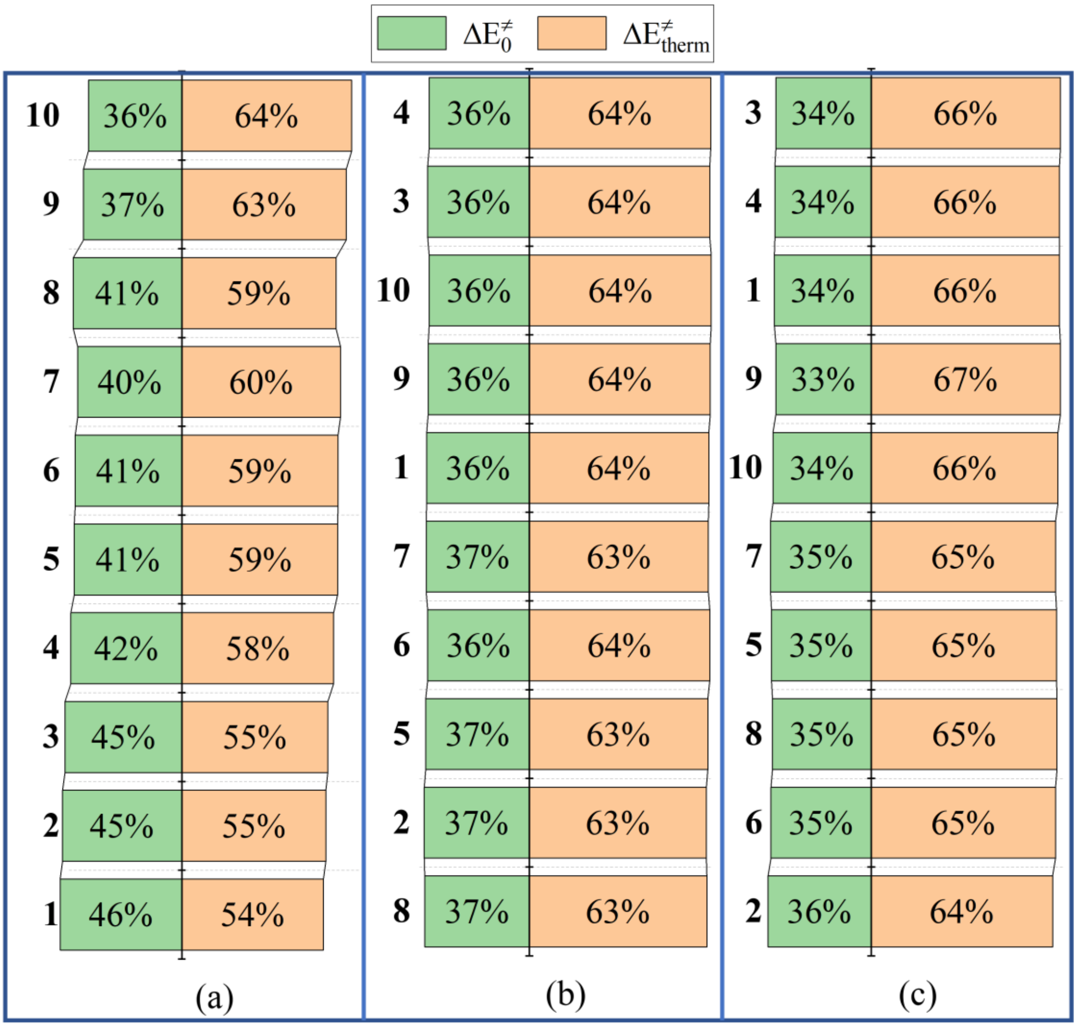

Figure S3. Percentage stacked bar chart of thermal contribution and intrinsic barrier as the components of activation barrier for the HAA reactions of NHC-boranes by $^{\bullet}CH_2CN$ (a), $Me^{\bullet}$ (b), and $Et^{\bullet}$ (c) at the M06-2X level.

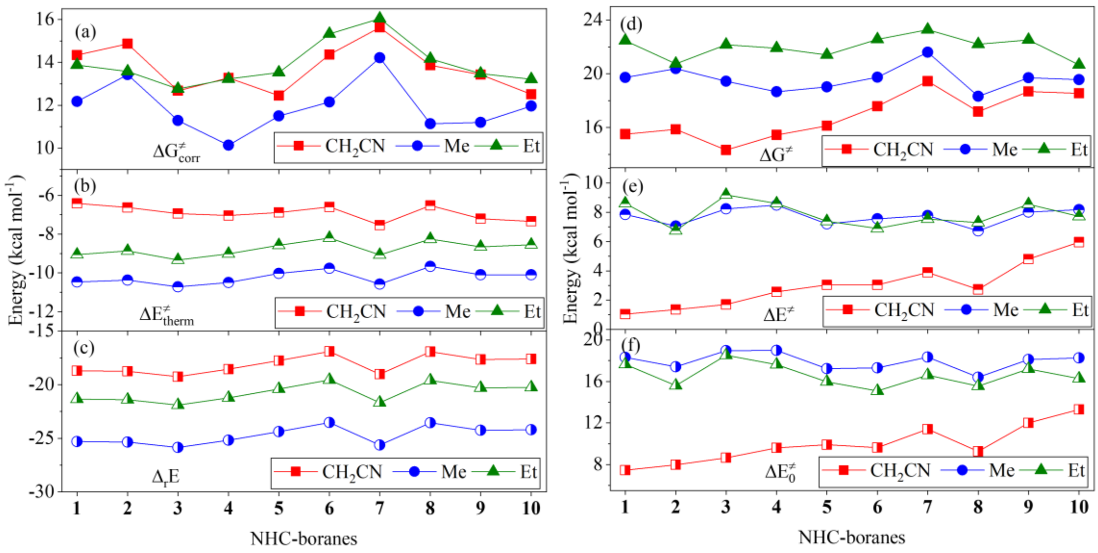

Figure S4. Computed activation Gibbs thermal corrections (a), thermal contributions (b), reaction energies (c), and free energy barriers (d), activation barriers (e), and intrinsic barriers (f) at the M06-2X level.

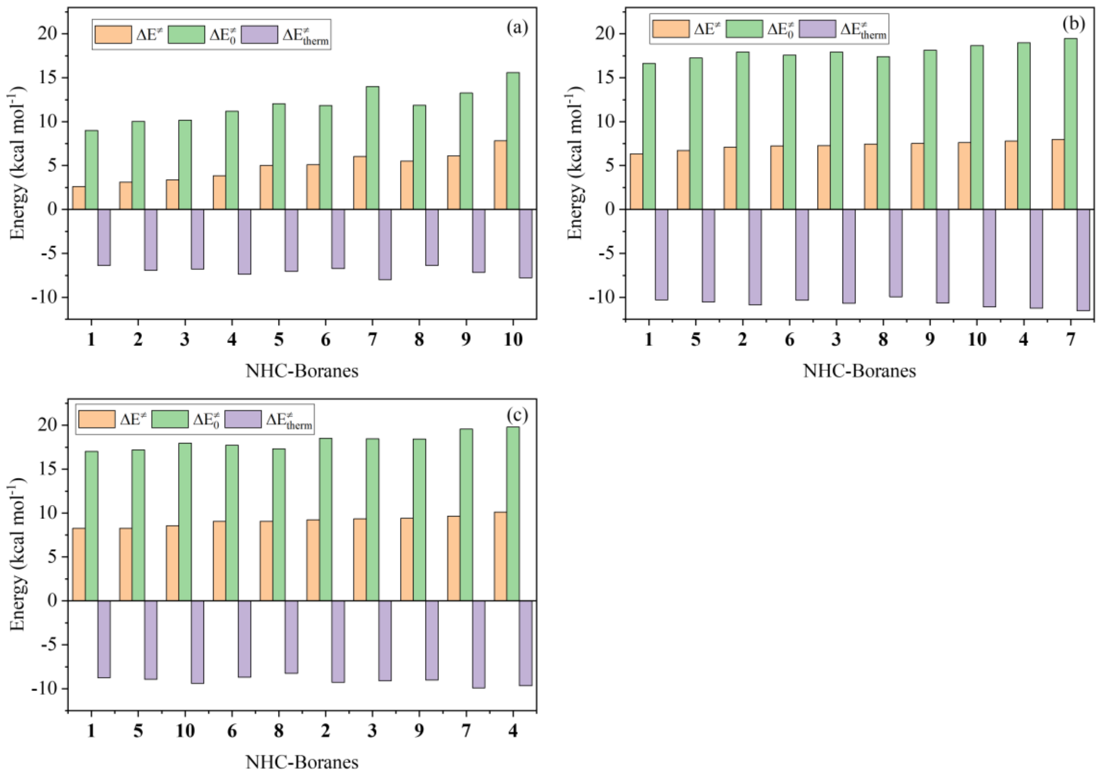

Figure S5. Vertical bar plots of thermal contributions, intrinsic barriers, and activation barriers for the HAA reactions of NHC-boranes by $^{\bullet}CH_2CN$ (a), Me$^{\bullet}$ (b), and Et$^{\bullet}$ (c) at the B3LYP level.

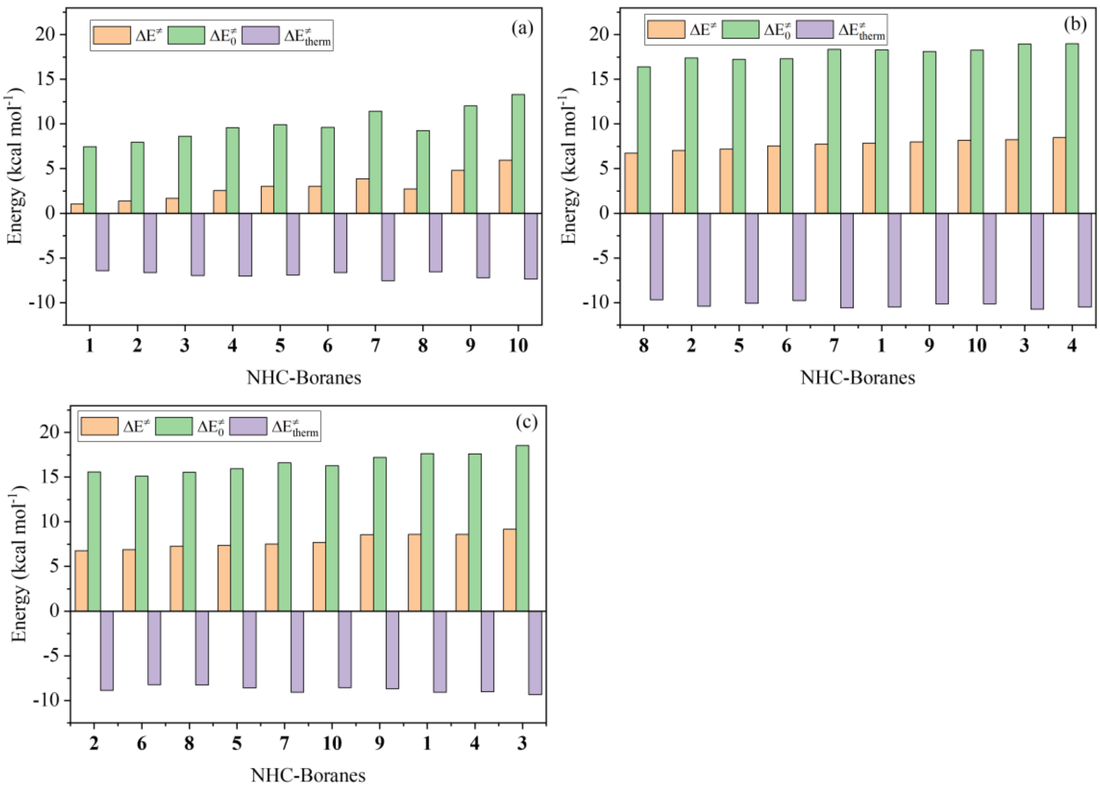

Figure S6. Vertical bar plots of thermal contributions, intrinsic barriers, and activation barriers for the HAA reactions of NHC-boranes by $^{\bullet}CH_2CN$ (a), Me$^{\bullet}$ (b), and Et$^{\bullet}$ (c) at the M06-2X level.

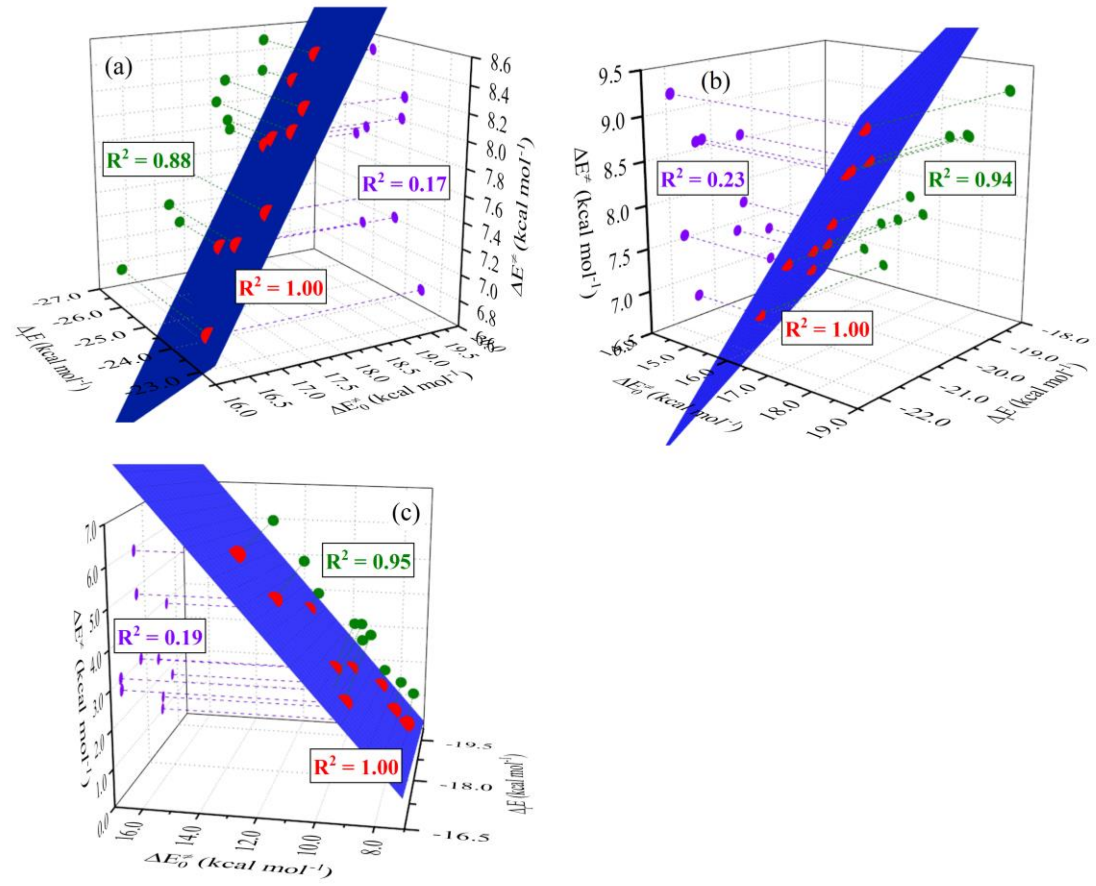

Figure S7. Dependence of activation barrier on intrinsic barrier and reaction energy using the suggested three-variable linear model at the M06-2X level. The R-squared values in red and the plane in blue represent the linear GOF of the computed data points (in red) and the image of the fitted equation using the suggested three-variable model. The R-squared values in olive and violet refer to the linear fitting CODs of the projection points of the computed data points on the corresponding coordinate planes.

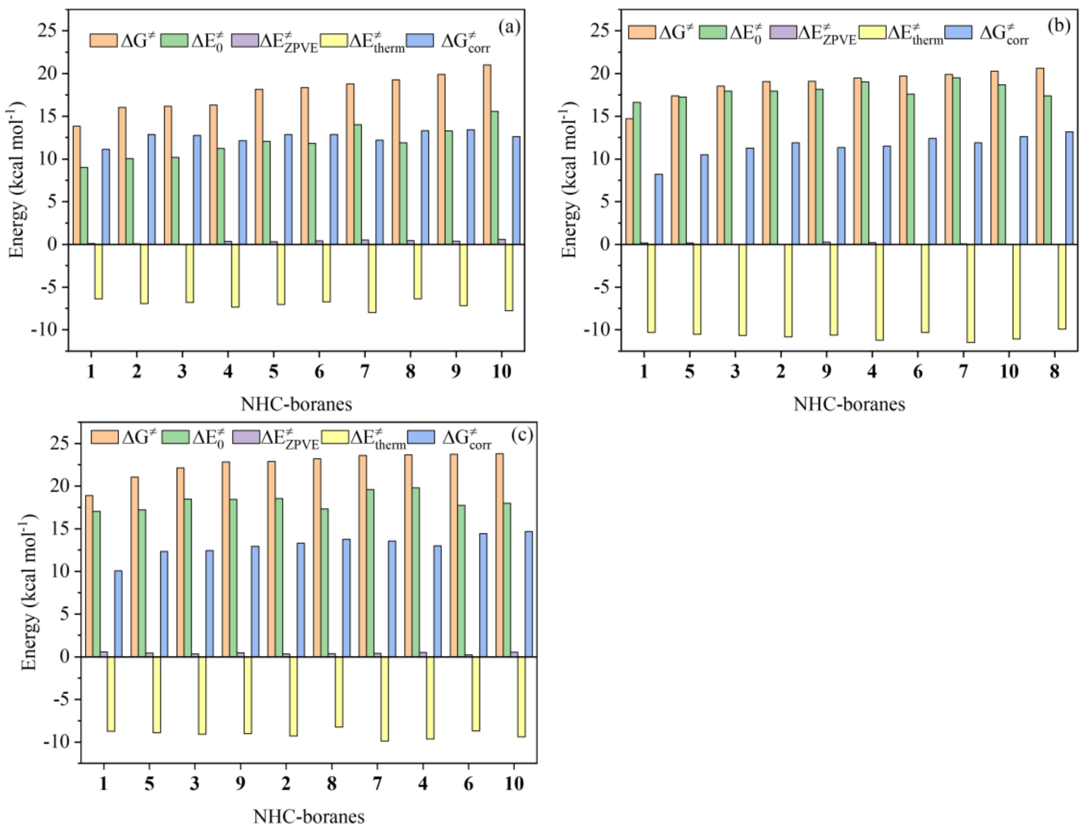

Figure S8. Vertical bar plots of activation Gibbs thermal corrections, thermal contributions, activation ZPVE corrections, intrinsic barriers, and free energy barriers for the HAA reactions of NHC-boranes by $^{\bullet}CH_2CN$ (a), $Me^{\bullet}$ (b), and $Et^{\bullet}$ (c) at the B3LYP level.

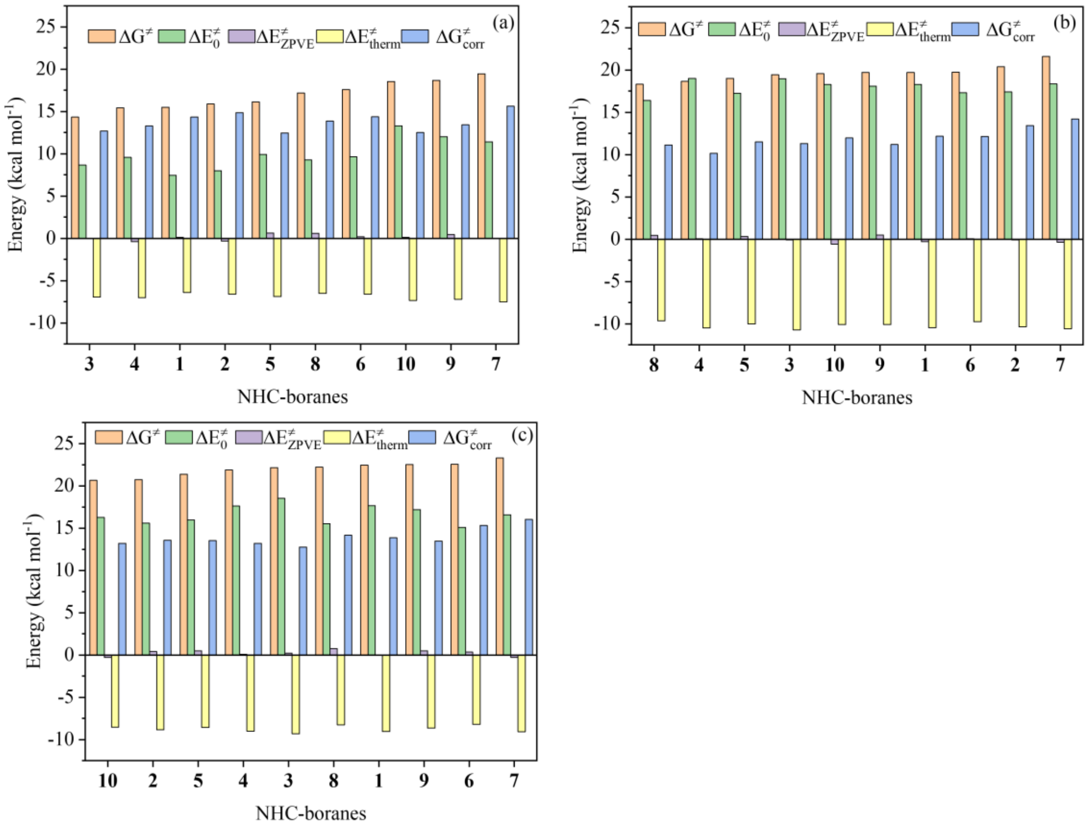

Figure S9. Vertical bar plots of activation Gibbs thermal corrections, thermal contributions, activation ZPVE corrections, intrinsic barriers, and free energy barriers for the HAA reactions of NHC-boranes by $^{\bullet}CH_2CN$ (a), Me$^{\bullet}$ (b), and Et$^{\bullet}$ (c) at the M06-2X level.

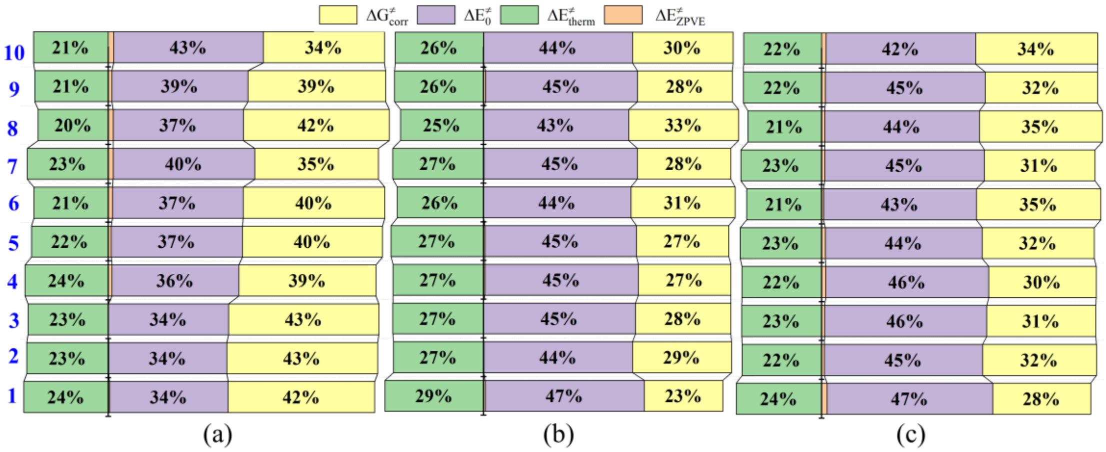

Figure S10. Percentage stacked bar chart of activation ZPVE corrections, thermal contributions, intrinsic barriers, and activation Gibbs thermal corrections as the components of free energy barriers for the HAA reactions of NHC-boranes by $^{\bullet}CH_2CN$ (a), Me$^{\bullet}$ (b), and Et$^{\bullet}$ (c) at the B3LYP level.

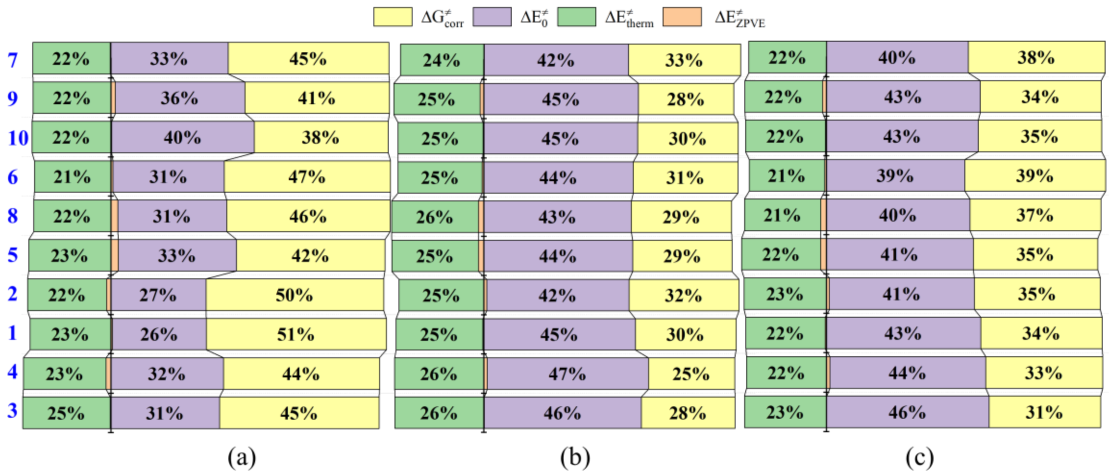

Figure S11. Percentage stacked bar chart of activation ZPVE corrections, thermal contributions, intrinsic barriers, and activation Gibbs thermal corrections as the components of free energy barriers for the HAA reactions of NHC-boranes by $^{\bullet}CH_2CN$ (a), $Me^{\bullet}$ (b), and $Et^{\bullet}$ (c) at the M06-2X level.

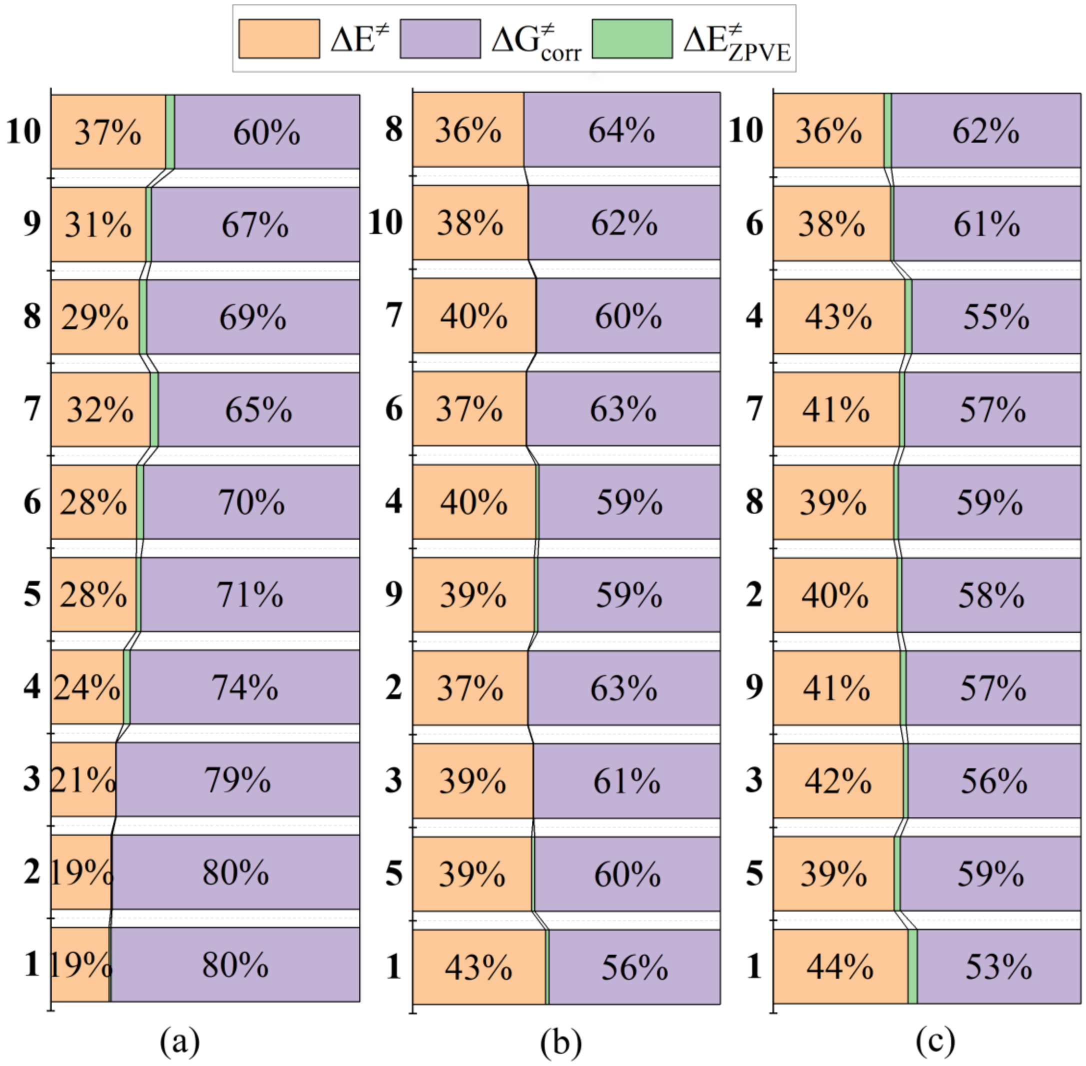

Figure S12. Percentage stacked bar chart of activation ZPVE corrections, activation barriers, and activation Gibbs thermal corrections as the components of free energy barriers for the HAA reactions of NHC-boranes by $^{\bullet}CH_2CN$ (a), $Me^{\bullet}$ (b), and $Et^{\bullet}$ (c) at the B3LYP level.

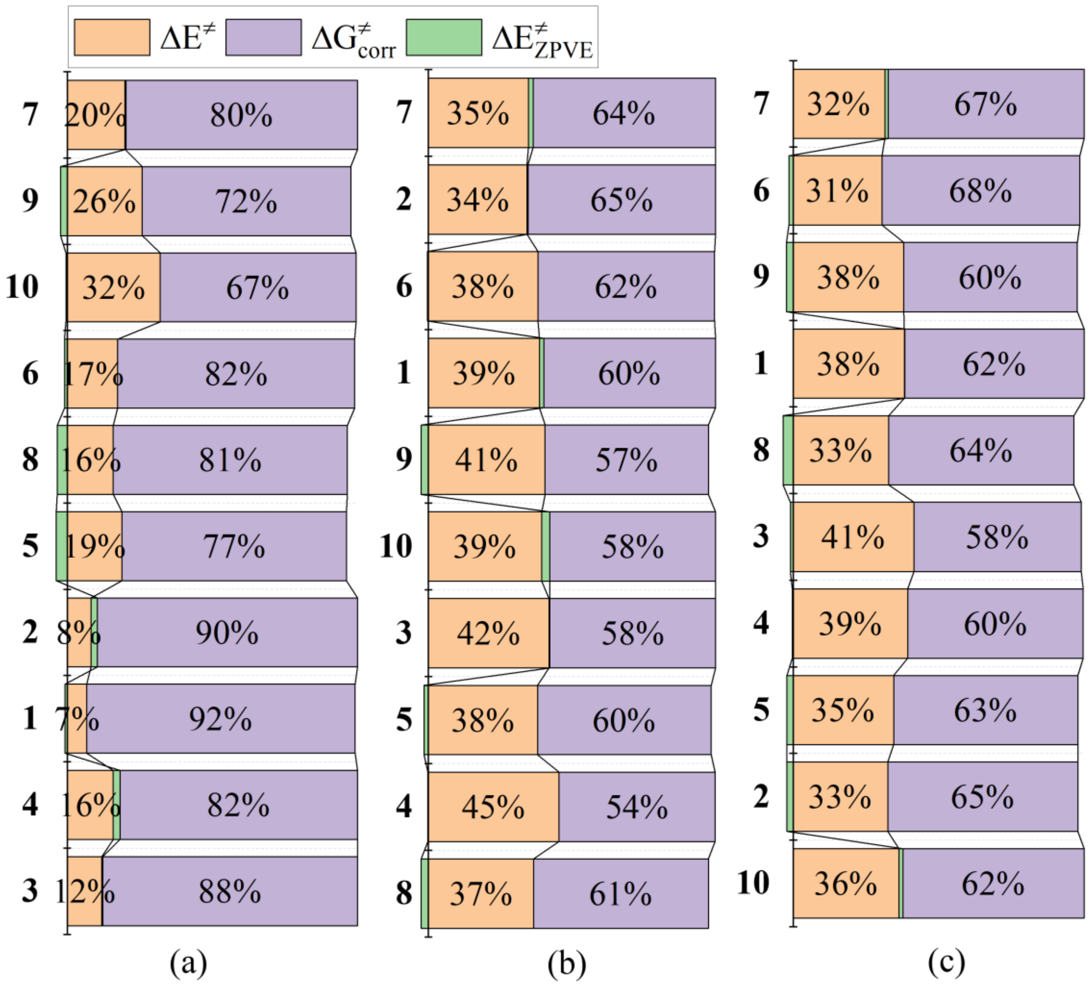

Figure S13. Percentage stacked bar chart of activation ZPVE corrections, activation barriers, and activation Gibbs thermal corrections as the components of free energy barriers for the HAA reactions of NHC-boranes by $^{\bullet}CH_2CN$ (a), $Me^{\bullet}$ (b), and $Et^{\bullet}$ (c) at the M06-2X level.

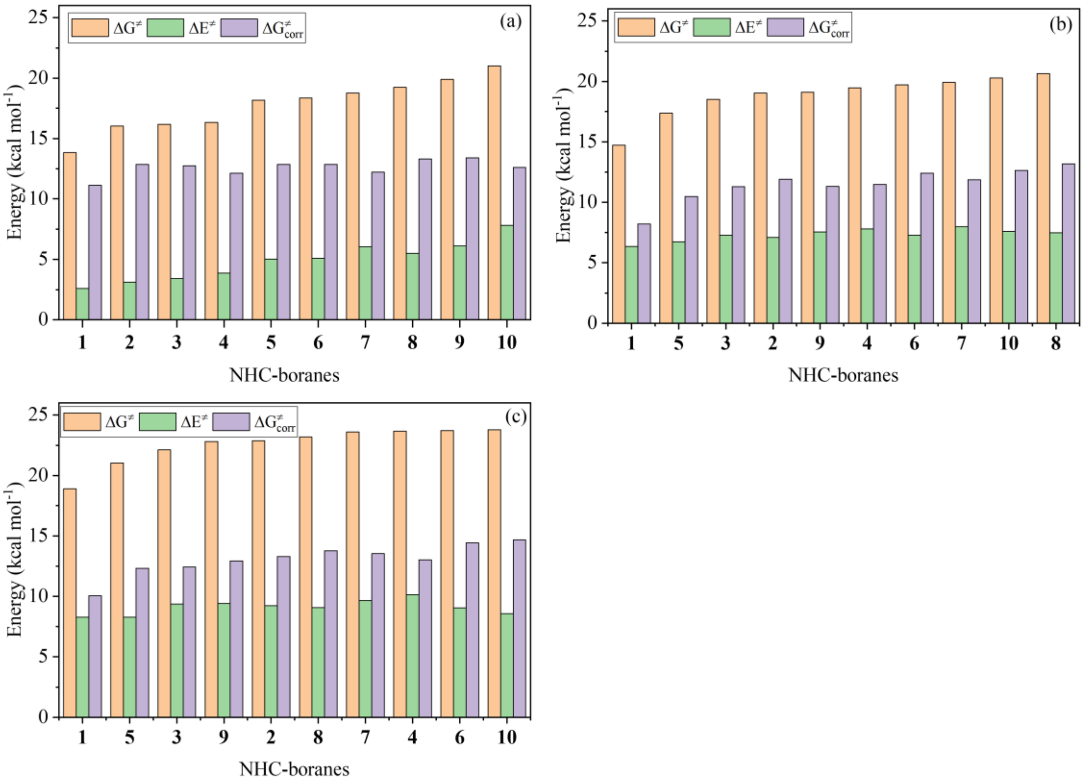

Figure S14. Bar chart of activation barriers, free energy barriers, and activation Gibbs thermal corrections for the HAA reactions of NHC-boranes by $^{\bullet}CH_2CN$ (a), $Me^{\bullet}$ (b), and $Et^{\bullet}$ (c) at the B3LYP level.

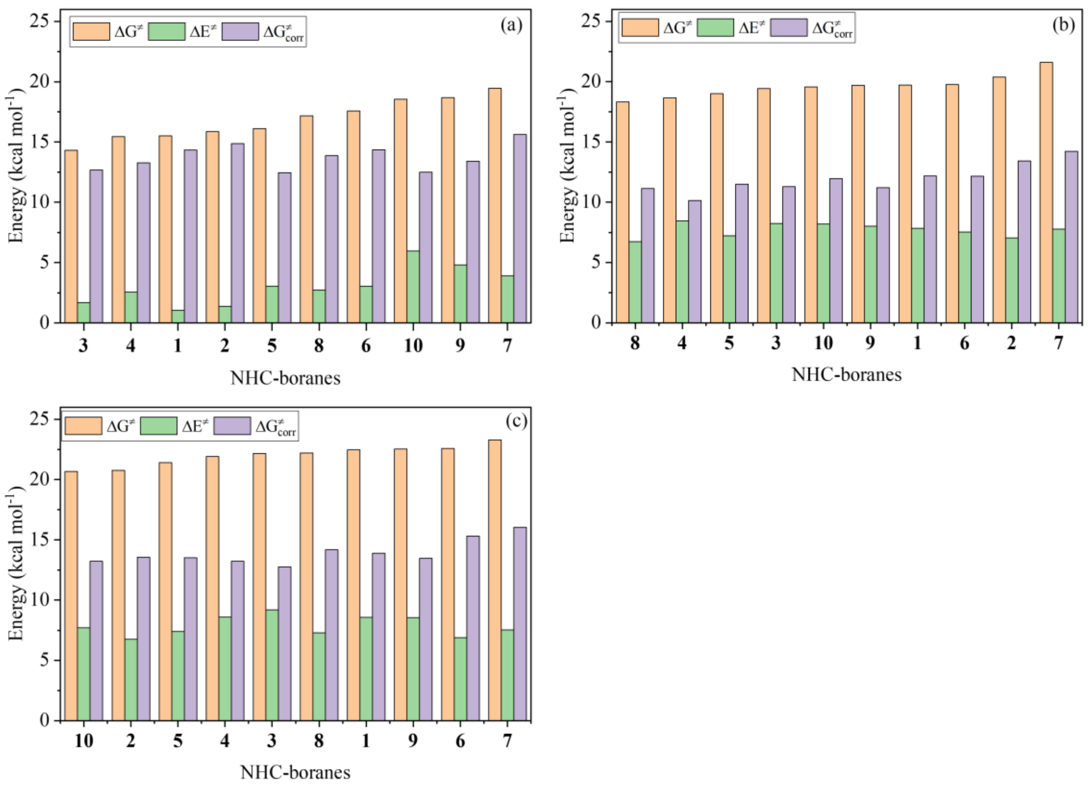

Figure S15. Bar chart of activation barriers, free energy barriers, and activation Gibbs thermal corrections for the HAA reactions of NHC-boranes by ${}^{\bullet}CH_2CN$ (a), Me$^{\bullet}$ (b), and Et$^{\bullet}$ (c) at the M06-2X level.

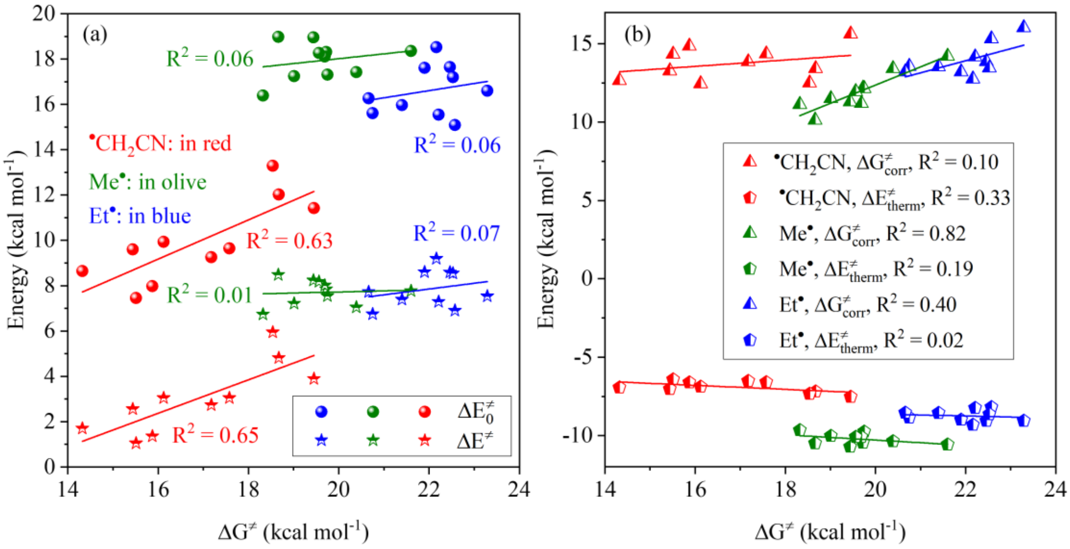

Figure S16. Linear correlation analysis of free energy barrier with intrinsic and activation barriers (a) and with activation Gibbs thermal corrections and thermal contributions (b) at the M06-2X level.

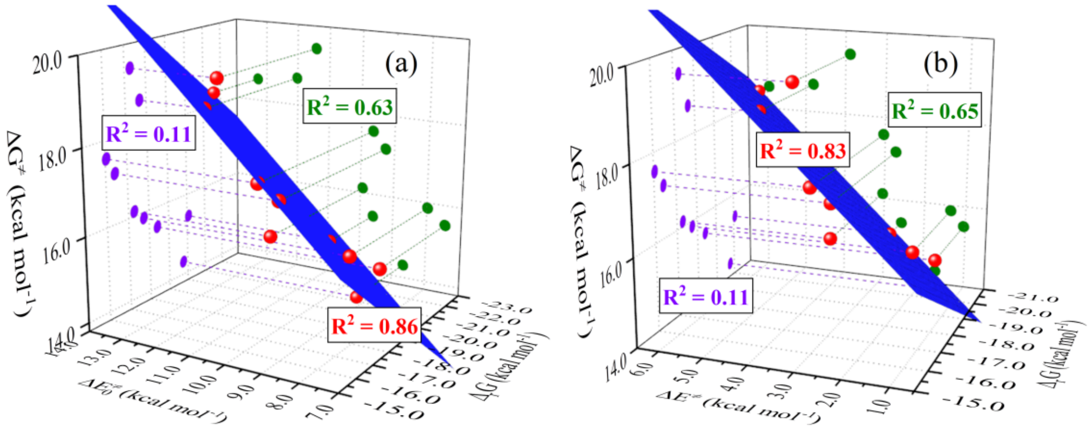

Figure S17. Dependence of free energy barrier on reaction Gibbs free energy and intrinsic barrier (a)/activation barrier (b) using the suggested three-variable linear model at the M06-2X level. The R-squared values in red and the plane in blue represent the linear GOF of the computed data points (in red) and the image of the fitted equation using the suggested three-variable linear model. The R-squared values in olive and violet refer to the linear fitting CODs of the projection points of the computed data points on the corresponding coordinate planes.

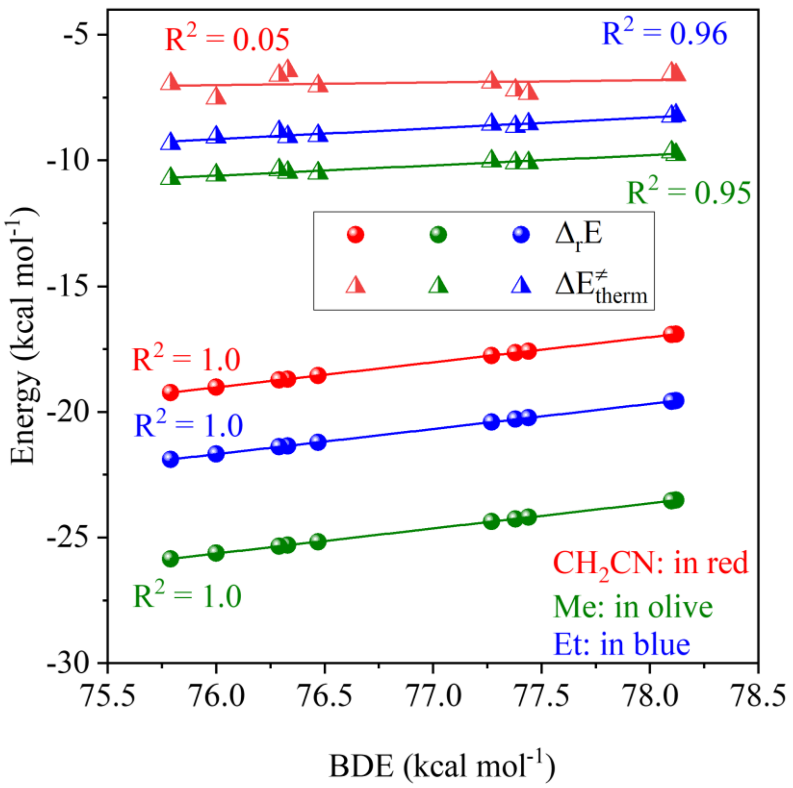

Figure S18. Linear correlation analysis of BDEs with reaction energies and thermal contributions at the M06-2X level.

Table S1. Total electronic energies (TE, hartree), zero-point vibrational energies (ZPVE, hartree/particle), Gibbs free energies (G, hartree), enthalpies (H, hartree), thermal corrections to Gibbs free energy (G_{corr}, hartree), and thermal corrections to enthalpy (H_{corr}, hartree) of the structures of NHC-boranes, NHC-boryl radicals, attacking radical $R^{•}$ and RH optimized at the B3LYP/6-311++G(d,p) level of theory in BTF at 376 K.

Species	TE	ZPVE	H	G	H_{coor}	G_{coor}
**1**	-331.598609	0.158913	-331.425269	-331.484030	0.173340	0.114579
**2**	-562.714687	0.241219	-562.451551	-562.531551	0.263136	0.183136
**3**	-430.929220	0.153135	-430.760217	-430.825111	0.169003	0.104109
**4**	-662.048342	0.235679	-661.789692	-661.871352	0.258650	0.176990
**5**	-423.905043	0.160229	-423.727771	-423.795270	0.177272	0.109773
**6**	-502.566091	0.215403	-502.328670	-502.408627	0.237421	0.157464
**7**	-655.018473	0.242222	-654.751799	-654.837484	0.266674	0.180989
**8**	-581.210643	0.272478	-580.912986	-581.000615	0.297657	0.210028
**9**	-523.231271	0.154042	-523.058875	-523.130360	0.172396	0.100911
**10**	-516.199254	0.160785	-516.018822	-516.093108	0.180432	0.106146
**R1**	-330.964966	0.148261	-330.802167	-330.862962	0.162799	0.102004
**R2**	-562.083303	0.230953	-561.830851	-561.908551	0.252452	0.174751
**R3**	-430.296629	0.142531	-430.138401	-430.202922	0.158228	0.093707
**R4**	-661.417338	0.224612	-661.169850	-661.251401	0.247488	0.165937
**R5**	-423.272422	0.149815	-423.106003	-423.171992	0.166419	0.100429
**R6**	-501.931890	0.204738	-501.705362	-501.784418	0.226528	0.147472
**R7**	-654.389370	0.231903	-654.133545	-654.217147	0.255824	0.172223
**R8**	-580.574954	0.262103	-580.287990	-580.373720	0.286964	0.201234
**R9**	-522.598108	0.143181	-522.436836	-522.508095	0.161272	0.090013
**R10**	-515.568566	0.150554	-515.399017	-515.471846	0.169549	0.096720
$Me^{•}$	-39.855770	0.029550	-39.821029	-39.852370	0.034741	0.003401
$Et^{•}$	-79.185621	0.058826	-79.120105	-79.158902	0.065516	0.026720
$^{•}CH_2CN$	-132.139999	0.030847	-132.103102	-132.141013	0.036897	-0.001014
CH_4	-40.534131	0.044375	-40.484839	-40.512734	0.049292	0.021397
CH_3CH_3	-79.856731	0.074104	-79.776540	-79.813149	0.080192	0.043582
CH_3CN	-132.803769	0.045139	-132.752464	-132.790282	0.051305	0.013486

Table S2. Total electronic energies (TE, hartree), zero-point vibrational energies (ZPVE, hartree/particle), Gibbs free energies (G, hartree), enthalpies (H, hartree), thermal corrections to Gibbs free energy (G_{corr}, hartree), and thermal corrections to enthalpy (H_{corr}, hartree) of the structures of NHC-boranes, NHC-boryl radicals, attacking radical $R^•$ and RH optimized at the M06-2X/6-311++G(d,p) level of theory in BTF at 376 K.

Species	TE	ZPVE	H	G	H_{coor}	G_{coor}
**1**	-331.439771	0.160249	-331.264753	-331.325221	0.175017	0.114549
**2**	-562.464829	0.243972	-562.199352	-562.277625	0.265477	0.187203
**3**	-430.743689	0.155001	-430.574152	-430.635390	0.169536	0.108298
**4**	-661.772777	0.238142	-661.513147	-661.589792	0.259631	0.182985
**5**	-423.722223	0.162362	-423.543122	-423.608962	0.179101	0.113261
**6**	-502.342687	0.217999	-502.103001	-502.179983	0.239686	0.162704
**7**	-654.745764	0.244562	-654.476935	-654.562621	0.268829	0.183144
**8**	-580.949379	0.275743	-580.648946	-580.732659	0.300433	0.216720
**9**	-523.022433	0.156600	-522.848071	-522.916502	0.174362	0.105932
**10**	-515.992214	0.162132	-515.812650	-515.880793	0.179563	0.111421
**R1**	-330.809395	0.149728	-330.645288	-330.705254	0.164107	0.104142
**R2**	-561.834536	0.233476	-561.579876	-561.656576	0.254660	0.177960
**R3**	-430.113969	0.144275	-429.954271	-430.017562	0.159698	0.096407
**R4**	-661.141716	0.227156	-660.892044	-660.972472	0.249671	0.169244
**R5**	-423.090303	0.151804	-422.922199	-422.987181	0.168105	0.103122
**R6**	-501.709949	0.207974	-501.480988	-501.556245	0.228961	0.153704
**R7**	-654.116244	0.234382	-653.858319	-653.940833	0.257926	0.175411
**R8**	-580.315743	0.264781	-580.026653	-580.109509	0.289090	0.206234
**R9**	-522.388730	0.144435	-522.227453	-522.295363	0.161277	0.093367
**R10**	-515.360723	0.152267	-515.189651	-515.263963	0.171072	0.096760
$Me^•$	-39.821536	0.029624	-39.786634	-39.818129	0.034902	0.003407
$Et^•$	-79.128081	0.059240	-79.062115	-79.100938	0.065967	0.027143
$^•CH_2CN$	-132.076365	0.031249	-132.038905	-132.077104	0.037460	-0.000739
CH_4	-40.497045	0.044942	-40.447192	-40.475075	0.049854	0.021970
CH_3CH_3	-79.797300	0.074572	-79.716622	-79.753269	0.080678	0.044031
CH_3CN	-132.740336	0.045562	-132.688618	-132.726417	0.051717	0.013919

Table S3. Total electronic energies (TE, hartree), zero-point vibrational energies (ZPVE, hartree/particle), Gibbs free energies (G, hartree), enthalpies (H, hartree), thermal corrections to Gibbs free energy (G_{corr}, hartree), and thermal corrections to enthalpy (H_{corr}, hartree) of the transition states (**TS**) of the H-atom abstraction reactions of NHC-boranes by Me$^{•}$ optimized at the B3LYP/6-311++G(d,p) and M06-2X/6-311++G(d,p) levels of theory in BTF at 376 K.

**TS**	TE	ZPVE	H	G	H_{coor}	G_{coor}
B3LYP/6-311++G(d,p)						
**TS1**	-371.443995	0.188197	-371.236539	-371.312926	0.207456	0.131069
**TS2**	-602.559080	0.270704	-602.262028	-602.35356	0.297052	0.205520
**TS3**	-470.773440	0.182738	-470.570444	-470.647958	0.202996	0.125482
**TS4**	-701.891398	0.264934	-701.598953	-701.692701	0.292445	0.198697
**TS5**	-463.749830	0.189518	-463.538794	-463.619946	0.211036	0.129884
**TS6**	-542.410230	0.244907	-542.139000	-542.229585	0.271230	0.180645
**TS7**	-694.861449	0.271695	-694.560974	-694.658120	0.300475	0.203329
**TS8**	-621.054518	0.302045	-620.723007	-620.820093	0.331511	0.234425
**TS9**	-563.074640	0.183187	-562.868624	-562.952281	0.206016	0.122359
**TS10**	-556.042856	0.190295	-555.828760	-555.913181	0.214096	0.129675
M06-2X/6-311++G(d,p)						
**TS1**	-371.249277	0.190345	-371.040159	-371.111925	0.209118	0.137352
**TS2**	-602.275275	0.273742	-601.975925	-602.063263	0.299351	0.212013
**TS3**	-470.552233	0.184756	-470.347600	-470.422541	0.204633	0.129692
**TS4**	-701.580740	0.267706	-701.285972	-701.378185	0.294768	0.202555
**TS5**	-463.531782	0.191504	-463.319184	-463.396794	0.212599	0.134989
**TS6**	-542.152116	0.247550	-541.878667	-541.966641	0.273449	0.185476
**TS7**	-694.555514	0.274777	-694.252633	-694.346324	0.302881	0.209190
**TS8**	-620.759465	0.304647	-620.425633	-620.521590	0.333832	0.237875
**TS9**	-562.830438	0.185462	-562.622719	-562.703246	0.207720	0.127192
**TS10**	-555.801640	0.192674	-555.585753	-555.667750	0.215886	0.133890

Table S4. Total electronic energies (TE, hartree), zero-point vibrational energies (ZPVE, hartree/particle), Gibbs free energies (G, hartree), enthalpies (H, hartree), thermal corrections to Gibbs free energy (G_{corr}, hartree), and thermal corrections to enthalpy (H_{corr}, hartree) of the transition states (**TS**) of the H-atom abstraction reactions of NHC-boranes by $Et^{\bullet}$ optimized at the B3LYP/6-311++G(d,p) and M06-2X/6-311++G(d,p) levels of theory in BTF at 376 K.

**TS**	TE	ZPVE	H	G	H_{coor}	G_{coor}
B3LYP/6-311++G(d,p)						
**TS11**	-410.7701344	0.216844	-410.532177	-410.612824	0.237957	0.157310
**TS12**	-641.885068	0.299520	-641.557395	-641.653996	0.327673	0.231072
**TS13**	-510.099382	0.211431	-509.865807	-509.948745	0.233575	0.150637
**TS14**	-741.217004	0.293708	-740.893920	-740.992550	0.323084	0.224454
**TS15**	-503.076804	0.218388	-502.835111	-502.920661	0.241693	0.156142
**TS16**	-581.736929	0.273878	-581.435004	-581.529735	0.301925	0.207194
**TS17**	-734.188075	0.300424	-733.856996	-733.958773	0.331079	0.229302
**TS18**	-660.381254	0.330747	-660.019092	-660.122559	0.362162	0.258695
**TS19**	-602.401173	0.212150	-602.164412	-602.252913	0.236761	0.148260
**TS20**	-595.370343	0.218744	-595.126846	-595.214094	0.243497	0.156249
M06-2X/6-311++G(d,p)						
**TS11**	-410.554173	0.219499	-410.314145	-410.390362	0.240028	0.163811
**TS12**	-641.581459	0.302518	-641.251302	-641.345491	0.330157	0.235968
**TS13**	-509.856790	0.213911	-509.621211	-509.701013	0.235579	0.155777
**TS14**	-740.887035	0.297269	-740.561171	-740.655838	0.325864	0.231197
**TS15**	-502.837760	0.220838	-502.594154	-502.675791	0.243605	0.161969
**TS16**	-581.459215	0.276674	-581.156013	-581.244951	0.303202	0.214264
**TS17**	-733.862288	0.304248	-733.528211	-733.626444	0.334078	0.235845
**TS18**	-660.064624	0.333769	-659.700952	-659.798201	0.363672	0.266422
**TS19**	-602.136078	0.215025	-601.897130	-601.981533	0.238949	0.154545
**TS20**	-595.108418	0.221796	-594.861456	-594.948806	0.246962	0.159612

Table S5. Total electronic energies (TE, hartree), zero-point vibrational energies (ZPVE, hartree/particle), Gibbs free energies (G, hartree), enthalpies (H, hartree), thermal corrections to Gibbs free energy (G_{corr}, hartree), and thermal corrections to enthalpy (H_{corr}, hartree) of the transition states (**TS**) of the H-atom abstraction reactions of NHC-boranes by $^{\bullet}CH_2CN$ optimized at the B3LYP/6-311++G(d,p) and M06-2X/6-311++G(d,p) levels of theory in BTF at 376 K.

TS	TE	ZPVE	H	G	H_{coor}	G_{coor}
B3LYP/6-311++G(d,p)						
**TS21**	-463.734286	0.189598	-463.524024	-463.602989	0.210262	0.131297
**TS22**	-694.849614	0.271967	-694.549680	-694.647021	0.299934	0.202593
**TS23**	-563.063770	0.183954	-562.858033	-562.940363	0.205737	0.123407
**TS24**	-794.181636	0.265963	-793.886440	-793.986336	0.295196	0.195300
**TS25**	-556.036572	0.190610	-555.823012	-555.907333	0.213560	0.129239
**TS26**	-634.697320	0.245613	-634.423623	-634.520383	0.273697	0.176937
**TS27**	-787.148058	0.272282	-786.845136	-786.948602	0.302922	0.199456
**TS28**	-713.341155	0.302612	-713.007282	-713.110950	0.333873	0.230205
**TS29**	-655.360940	0.184316	-655.152338	-655.239653	0.208602	0.121287
**TS30**	-648.325868	0.190708	-648.109533	-648.200643	0.216335	0.125225
M06-2X/6-311++G(d,p)						
**TS21**	-463.514272	0.191290	-463.304425	-463.377602	0.209847	0.136670
**TS22**	-694.539579	0.275772	-694.236658	-694.329433	0.302921	0.210146
**TS23**	-562.817418	0.186327	-562.609664	-562.689670	0.207754	0.127748
**TS24**	-793.845703	0.270030	-793.547372	-793.642292	0.298331	0.203411
**TS25**	-555.792739	0.192607	-555.577392	-555.660380	0.215347	0.132359
**TS26**	-634.413920	0.248963	-634.137631	-634.229073	0.276289	0.184847
**TS27**	-786.816031	0.275911	-786.510324	-786.608722	0.305708	0.207309
**TS28**	-713.020449	0.306051	-712.683797	-712.782382	0.336652	0.238067
**TS29**	-655.090429	0.187150	-654.879639	-654.963846	0.210790	0.126583
**TS30**	-648.058962	0.193242	-647.840658	-647.928350	0.218303	0.130611

Table S6. Activation barrier ($\Delta E^{\neq}$), activation zero-point vibrational energy correction ($\Delta E^{\neq}_{ZPVE}$), intrinsic barrier ($\Delta E^{\neq}_{0}$), thermal contribution ($\Delta E^{\neq}_{therm}$), activation Gibbs free energy correction ($\Delta G^{\neq}_{corr}$), and Gibbs free energy barrier ($\Delta G^{\neq}$) of the H-atom abstraction reactions of NHC-boranes by Me$^{\bullet}$, Et$^{\bullet}$, and $^{\bullet}CH_2CN$ computed at the B3LYP/6-311++G(d,p) level of theory in BTF at 376 K. All units are in kcal mol^{-1}.

NHC-boranes	$\Delta E^{\neq}$	$\Delta E^{\neq}_{ZPVE}$	$\Delta E^{\neq}_{0}$	$\Delta E^{\neq}_{therm}$	$\Delta G^{\neq}_{corr}$	$\Delta G^{\neq}$	$\Delta_r E$	$\Delta_r G$	$\Delta_r H$
Me$^{\bullet}$									
**1**	6.35	-0.17	16.64	-10.29	8.21	14.73	-25.44	-24.66	-25.54
**2**	7.10	-0.04	17.94	-10.84	11.91	19.05	-26.62	-23.45	-27.05
**3**	7.28	0.03	17.95	-10.67	11.28	18.53	-26.07	-23.96	-26.35
**4**	7.79	-0.19	19.01	-11.22	11.49	19.47	-27.36	-25.36	-27.59
**5**	6.73	-0.16	17.26	-10.53	10.49	17.38	-25.93	-23.27	-26.38
**6**	7.27	-0.03	17.58	-10.31	12.41	19.71	-25.10	-22.69	-25.42
**7**	7.98	-0.05	19.49	-11.51	11.88	19.91	-28.08	-25.12	-28.59
**8**	7.47	0.01	17.40	-9.93	13.18	20.64	-23.98	-21.00	-24.36
**9**	7.53	-0.25	18.16	-10.63	11.32	19.11	-25.87	-23.91	-26.21
**10**	7.61	-0.03	18.68	-11.07	12.63	20.27	-27.03	-24.54	-27.61
Et$^{\bullet}$									
**1**	8.28	-0.56	17.03	-8.75	10.05	18.89	-20.61	-20.82	-20.92
**2**	9.23	-0.33	18.52	-9.29	13.31	22.88	-21.78	-19.61	-22.42
**3**	9.37	-0.33	18.46	-9.09	12.43	22.13	-21.24	-20.12	-21.72
**4**	10.14	-0.50	19.80	-9.66	13.02	23.66	-22.52	-21.52	-22.96
**5**	8.28	-0.42	17.21	-8.93	12.33	21.03	-21.10	-19.43	-21.75
**6**	9.06	-0.22	17.74	-8.69	14.44	23.72	-20.27	-18.85	-20.79
**7**	9.66	-0.39	19.56	-9.90	13.55	23.60	-23.25	-21.28	-23.96
**8**	9.07	-0.35	17.32	-8.25	13.77	23.19	-19.15	-17.16	-19.73
**9**	9.41	-0.45	18.43	-9.02	12.94	22.81	-21.04	-20.07	-21.58
**10**	8.57	-0.54	17.96	-9.38	14.67	23.79	-22.20	-20.70	-22.99
$^{\bullet}CH_2CN$									
**1**	2.61	-0.10	9.00	-6.39	11.13	13.84	-16.62	-17.70	-16.48
**2**	3.12	-0.06	10.05	-6.93	12.85	16.03	-17.80	-16.48	-17.99
**3**	3.40	-0.02	10.20	-6.80	12.75	16.17	-17.25	-16.99	-17.29
**4**	3.85	-0.35	11.21	-7.35	12.13	16.33	-18.54	-18.40	-18.52
**5**	5.02	-0.29	12.06	-7.04	12.85	18.17	-17.11	-16.31	-17.32
**6**	5.10	-0.40	11.84	-6.74	12.86	18.36	-16.28	-15.73	-16.35
**7**	6.04	-0.49	14.02	-7.98	12.22	18.76	-19.26	-18.16	-19.52
**8**	5.51	-0.45	11.88	-6.37	13.30	19.25	-15.16	-14.04	-15.29
**9**	6.12	-0.36	13.28	-7.16	13.42	19.90	-17.05	-16.95	-17.15
**10**	7.82	-0.58	15.60	-7.78	12.61	21.01	-18.21	-17.57	-18.55

Table S7. Activation barrier ($\Delta E^{\neq}$), activation zero-point vibrational energy correction ($\Delta E^{\neq}_{ZPVE}$), intrinsic barrier ($\Delta E^{\neq}_{0}$), thermal contribution ($\Delta E^{\neq}_{therm}$), activation Gibbs free energy correction ($\Delta G^{\neq}_{corr}$), and free energy barrier ($\Delta G^{\neq}$) of the H-atom abstraction reactions of NHC-boranes by Me$^{\bullet}$, Et$^{\bullet}$, and $^{\bullet}CH_2CN$ computed at the M06-2X/6-311++G(d,p) level of theory in BTF at 376 K. All units are in kcal mol^{-1}.

NHC-boranes	$\Delta E^{\neq}$	$\Delta E^{\neq}_{ZPVE}$	$\Delta E^{\neq}_{0}$	$\Delta E^{\neq}_{therm}$	$\Delta G^{\neq}_{corr}$	$\Delta G^{\neq}$	$\Delta_r E$	$\Delta_r G$	$\Delta_r H$
Me$^{\bullet}$									
**1**	7.85	0.30	18.31	-10.47	12.17	19.72	-25.31	-23.20	-25.79
**2**	7.05	0.09	17.42	-10.37	13.43	20.39	-25.35	-22.53	-25.78
**3**	8.23	0.08	18.96	-10.72	11.29	19.44	-25.85	-24.55	-25.53
**4**	8.48	-0.04	18.98	-10.50	10.14	18.66	-25.17	-24.87	-24.76
**5**	7.21	-0.30	17.24	-10.03	11.50	19.01	-24.37	-22.07	-24.87
**6**	7.55	-0.05	17.31	-9.76	12.15	19.75	-23.52	-20.84	-24.19
**7**	7.77	0.37	18.35	-10.58	14.21	21.60	-25.63	-22.06	-26.32
**8**	6.73	-0.45	16.39	-9.66	11.14	18.32	-23.54	-21.21	-24.01
**9**	8.01	-0.48	18.11	-10.10	11.20	19.69	-24.26	-22.47	-25.07
**10**	8.18	0.58	18.27	-10.10	11.96	19.56	-24.20	-25.17	-23.57
Et$^{\bullet}$									
**1**	8.59	0.01	17.65	-9.06	13.88	22.46	-21.36	-20.31	-21.99
**2**	6.75	-0.44	15.61	-8.86	13.57	20.75	-21.39	-19.63	-21.98
**3**	9.19	-0.21	18.52	-9.33	12.76	22.16	-21.90	-21.65	-21.73
**4**	8.60	-0.07	17.61	-9.01	13.22	21.90	-21.22	-21.97	-20.96
**5**	7.39	-0.48	15.97	-8.57	13.53	21.40	-20.41	-19.17	-21.07
**6**	6.90	-0.35	15.09	-8.20	15.32	22.57	-19.56	-17.94	-20.39
**7**	7.53	0.28	16.60	-9.07	16.04	23.29	-21.68	-19.17	-22.52
**8**	7.29	-0.76	15.54	-8.25	14.16	22.21	-19.59	-18.31	-20.21
**9**	8.55	-0.51	17.20	-8.65	13.47	22.53	-20.30	-19.57	-21.27
**10**	7.72	0.27	16.27	-8.55	13.21	20.66	-20.24	-22.28	-19.77
$^{\bullet}CH_2CN$									
**1**	1.04	-0.13	7.46	-6.42	14.34	15.51	-18.70	-18.41	-18.98
**2**	1.36	0.35	7.98	-6.62	14.86	15.87	-18.74	-17.74	-18.97
**3**	1.70	0.05	8.65	-6.94	12.67	14.32	-19.24	-19.76	-18.72
**4**	2.56	0.40	9.60	-7.04	13.28	15.44	-18.56	-20.08	-17.95
**5**	3.04	-0.63	9.93	-6.89	12.45	16.12	-17.76	-17.28	-18.07
**6**	3.04	-0.18	9.64	-6.60	14.36	17.58	-16.91	-16.05	-17.38
**7**	3.89	0.06	11.42	-7.53	15.63	19.45	-19.02	-17.27	-19.51
**8**	2.73	-0.59	9.26	-6.53	13.86	17.18	-16.93	-16.42	-17.21
**9**	4.81	-0.44	12.02	-7.20	13.42	18.67	-17.65	-17.68	-18.26
**10**	5.95	-0.09	13.29	-7.34	12.51	18.54	-17.59	-20.38	-16.76

Table S8. Spin densities ($\rho_{s,B}$, *e*) on the central B atoms, global nucleophilicity (N, eV) and electrophilicity indices (ω, eV), and local nucleophilicity (N_B, eV) and electrophilicity indices (ω_B, eV) of the NHC-boranes and NHC-boryl radicals computed at the mPWPW91/6-311+G(d,p) level of theory in BTF.

Species	$\rho_{s,B}$	ω	ω_B	N	N_B
**1**	/	4.61	/	5.39	/
**2**	/	5.00	/	5.67	/
**3**	/	4.87	/	5.41	/
**4**	/	5.74	/	5.51	/
**5**	/	6.14	/	4.84	/
**6**	/	5.38	/	5.32	/
**7**	/	6.44	/	5.31	/
**8**	/	5.81	/	4.84	/
**9**	/	7.45	/	4.60	/
**10**	/	7.62	/	4.49	/
**R1**	0.68	19.20	13.05	7.87	5.35
**R2**	0.62	23.76	14.73	7.96	4.94
**R3**	0.52	18.09	9.41	8.17	4.25
**R4**	0.46	24.18	11.12	8.16	3.75
**R5**	0.66	29.62	19.55	7.29	4.81
**R6**	0.66	28.61	18.88	7.44	4.91
**R7**	0.66	31.87	21.03	7.42	4.89
**R8**	0.69	26.95	18.59	7.36	5.08
**R9**	0.52	29.72	15.46	7.53	3.92
**R10**	0.66	42.74	28.21	6.73	4.44

Table S9. CODs of the linear correlation of reaction energy ($\Delta_r E$) and thermal contribution ($\Delta E^{\neq}_{therm}$) with the spin density distributions of the HAA product NHC-boryl radicals. The basis set is 6-311++G(d,p).

$R^{\bullet}$	B3LYP		M06-2X	
	$\Delta_r E$	$\Delta E^{\neq}_{therm}$	$\Delta_r E$	$\Delta E^{\neq}_{therm}$
$Me^{\bullet}$	0.11	0.13	0.14	0.22
$Et^{\bullet}$	0.11	0.16	0.14	0.23
$^{\bullet}CH_2CN$	0.11	0.03	0.14	0.09

Table S10. CODs of the linear correlation of the reaction Gibbs free energies ($\Delta_r G$) with the free energy barriers ($\Delta G^{\neq}$) for the HAA reactions of NHC-boranes by different radicals. The basis set is 6-311++G(d,p).

$R^{\bullet}$	B3LYP	M06-2X
$Me^{\bullet}$	0.07	0.02
$Et^{\bullet}$	0.01	0.17
$^{\bullet}CH_2CN$	0.06	0.11

Table S11. Fitted equations and CODs using the linear three-variable model at the M06-2X/6-311++G(d,p) level of theory.

$R^{\bullet}$	$\Delta G^{\neq}$ vs ($\Delta E^{\neq}_0$, $\Delta_r G$)		$\Delta G^{\neq}$ vs ($\Delta E^{\neq}$, $\Delta_r G$)	
	Equations	R^2	Equations	R^2
$Me^{\bullet}$	$\Delta G^{\neq} = 1.05\Delta E^{\neq}_0 + 0.54\Delta_r G + 13.24$	0.85	$\Delta G^{\neq} = 2.75\Delta E^{\neq} + 0.31\Delta_r G + 12.61$	0.88
$Et^{\bullet}$	$\Delta G^{\neq} = 0.72\Delta E^{\neq}_0 + 0.59\Delta_r G + 11.70$	0.88	$\Delta G^{\neq} = 1.91\Delta E^{\neq} + 0.54\Delta_r G + 15.80$	0.84
$^{\bullet}CH_2CN$	$\Delta G^{\neq} = 0.81\Delta E^{\neq}_0 + 0.54\Delta_r G + 18.63$	0.86	$\Delta G^{\neq} = 0.93\Delta E^{\neq} + 0.48\Delta_r G + 22.76$	0.83

Table S12. Computed orbital interaction energies (kcal mol^{-1}) in the HAA transition states at the B3LYP/6-311++G(d,p) level of theory.

NHC-boranes	$^{\bullet}CH_2CN$		$Me^{\bullet}$		$Et^{\bullet}$	
	σ_{B-H} →SOMO(C)	SOMO(C)→ σ^*_{B-H}	σ_{B-H} →SOMO(C)	SOMO(C)→ σ^*_{B-H}	σ_{B-H} →SOMO(C)	SOMO(C)→ σ^*_{B-H}
**1**	72.52	6.73	52.28	4.20	55.68	5.20
**2**	70.85	6.86	55.02	4.43	56.32	5.23
**3**	78.95	8.62	56.29	5.44	58.88	6.40
**4**	80.51	8.48	60.21	5.76	61.84	6.51
**5**	74.18	7.76	48.85	4.33	51.74	5.38
**6**	79.70	8.12	/	/	57.35	5.94
**7**	82.22	8.17	49.05	4.22	51.90	5.11
**8**	81.95	8.35	54.12	4.84	57.50	6.03
**9**	81.24	9.38	54.92	5.64	57.14	6.69
**10**	68.86	7.73	44.27	4.16	46.18	5.15

Table S13. CODs of the linear correlation fittings of barriers with the global nucleophilicity/electrophilicity indices and charges.

Indices or charges	B3LYP6-311++G(d,p)			M06-2X/6-311++G(d,p)		
	$\Delta G^{\neq}$	$\Delta E^{\neq}$	$\Delta E_0^{\neq}$	$\Delta G^{\neq}$	$\Delta E^{\neq}$	$\Delta E_0^{\neq}$
$^{\bullet}CH_2CN$						
ω (NHC-boranes)	0.77	0.83	0.86	0.60	0.94	0.93
N (NHC-boranes)	0.65	0.67	0.52	0.31	0.64	0.56
Charges of borane fragments	0.36	0.34	0.21	0.34	0.17	0.10
Charges of the abstracted H atoms	0.68	0.76	0.64	0.47	0.59	0.51
ω (NHC-boryl radicals)	0.75	0.87	0.89	0.62	0.84	0.80
N (NHC-boryl radicals)	0.67	0.78	0.67	0.54	0.63	0.54
$Me^{\bullet}$						
ω (NHC-boranes)	0.24	0.29	0.25	0.01	0.04	0.01
N (NHC-boranes)	0.06	0.02	0.01	0.08	0.01	0.07
Charges of borane fragments	0.12	0.01	0.05	0.01	0.38	0.64
Charges of the abstracted H atoms	0.12	0.02	0.01	0.01	0.06	0.20
ω (NHC-boryl radicals)	0.28	0.21	0.20	0.02	0.01	0.03
N (NHC-boryl radicals)	0.12	0.03	0.01	0.01	0.05	0.17
$Et^{\bullet}$						
ω (NHC-boranes)	0.22	0.01	0.03	0.01	0.01	0.02
N (NHC-boranes)	0.02	0.15	0.14	0.02	0.01	0.03
Charges of borane fragments	0.06	0.21	0.27	0.01	0.61	0.79
Charges of the abstracted H atoms	0.06	0.24	0.16	0.02	0.18	0.29
ω (NHC-boryl radicals)	0.27	0.03	0.01	0.08	0.16	0.22
N (NHC-boryl radicals)	0.07	0.23	0.13	0.04	0.21	0.32

Table S14. CODs of the linear correlation fittings of the B3LYP- and M06-2X-computed BDEs and BDHs with the spin densities of the center B atoms of the product NHC-boryl radicals.

Levels of theory	BDE	BDH
Spin density: mPWPW91/6-311+G(d,p)		
B3LYP6-311++G(d,p)	0.11	0.08
M06-2X/6-311++G(d,p)	0.14	0.02
Spin density:B3LYP/6-311++G(d,p)		
B3LYP6-311++G(d,p)	0.04	0.02
M06-2X/6-311++G(d,p)	0.17	0.03
Spin density:M06-2X/6-311++G(d,p)		
B3LYP6-311++G(d,p)	0.01	0.01
M06-2X/6-311++G(d,p)	0.16	0.02

Relationship between the BEDs and HAA reaction energies for a series NHC-boranes

To determine the BDEs, it is essential to understand the method for predicting the BDEs using the HAA reactions with a given radical. For the closed-shell molecule AH, the A–H bond dissociation reaction is:

$$\mathrm{AH} \rightarrow \mathrm{A}^{\bullet} + \mathrm{H}^{\bullet} \tag{16}$$

The BDE of A–H can be defined as the reaction energy ($\Delta_r E(AH)$) for reaction (16), as follows:

$$\mathrm{BDE(A-H)} = \mathrm{E(A^{\bullet})} + \mathrm{E(H^{\bullet})} - \mathrm{E(AH)} \tag{17}$$

The HAA reaction of AH with radical $R^{\bullet}$ is:

$$\mathrm{AH} + \mathrm{R}^{\bullet} \rightarrow \mathrm{A}^{\bullet} + \mathrm{RH} \tag{18}$$

The corresponding reaction energy ($\Delta_r E(AR)$) can be expressed as:

$$\Delta_r \mathrm{E(AR)} = \mathrm{E(RH)} + \mathrm{E(A^{\bullet})} - \mathrm{E(AH)} - \mathrm{E(R^{\bullet})} \tag{19}$$

Subtracting reaction (16) from (18) gives the radical combination reaction:

$$\mathrm{R}^{\bullet} + \mathrm{H}^{\bullet} \rightarrow \mathrm{RH} \tag{20}$$

The corresponding reaction energy ($\Delta_r E(RH)$) is given by:

$$\Delta_r \mathrm{E(RH)} = \Delta_r \mathrm{E(AR)} - \mathrm{BDE(A-H)} \tag{21}$$

which can be rearranged as:

$$\mathrm{BDE(A-H)} = \Delta_r \mathrm{E(AR)} - \Delta_r \mathrm{E(RH)} \tag{22}$$

Note that $\Delta_r E(RH)$ is a constant for a given radical $R^{\bullet}$; thus, for a series of NHC-boranes, the BDEs are strictly linearly correlated with the reaction energies ($\Delta_r E(RH)$) of their HAA reactions with radical $R^{\bullet}$.

Table S15. List of Cartesian coordinates of located stationary points at the B3LYP/6-311++G(d,p) and M06-2X/6-311++G(d,p) levels of theory in BTF at 376 K.

Species		B3LYP/6-311++G(d,p)			M06-2X/6-311++G(d,p)		
		x	y	z	x	y	z
**1**	C	-0.011957	0.598409	0.000365	-0.009680	0.581684	0.001317
	C	0.737079	-1.530725	0.000118	0.723344	-1.541980	0.001192
	C	-0.618819	-1.576249	0.000422	-0.629884	-1.578706	-0.003845
	H	1.472095	-2.320468	-0.000002	1.452408	-2.333789	0.000455
	H	-1.298300	-2.414220	0.000511	-1.315858	-2.408056	-0.008864
	N	-1.057972	-0.267364	0.000542	-1.058104	-0.266895	-0.006069
	N	1.090678	-0.193425	0.000032	1.083844	-0.207812	0.003826
	C	-2.455760	0.130909	-0.000020	-2.447835	0.169138	0.006377
	H	-2.842031	0.140521	1.022424	-2.712369	0.547709	0.993606
	H	-2.524052	1.131312	-0.423819	-2.578751	0.961531	-0.728383
	H	-3.042627	-0.563614	-0.606592	-3.079844	-0.679435	-0.246338
	C	2.469889	0.267817	-0.000183	2.459438	0.278861	0.001870
	H	2.984130	-0.097605	-0.892794	2.627358	0.910679	-0.868092
	H	2.465159	1.356014	-0.000367	2.652467	0.852421	0.906526
	H	2.984303	-0.097287	0.892461	3.123336	-0.581882	-0.036185
	H	-0.752429	2.530273	0.997192	-0.840919	2.514071	0.904971
	H	0.976208	2.712933	0.001211	1.007990	2.654172	0.133236
	H	-0.750262	2.529885	-0.998600	-0.594241	2.519496	-1.065242
	B	-0.124747	2.195362	0.000031	-0.098810	2.182411	-0.002293
**2**	C	1.330245	-0.000401	0.468827	1.318501	-0.061042	0.471627
	C	3.251393	-0.122497	-0.706907	3.191035	0.209148	-0.736492
	C	2.800828	1.152359	-0.795176	2.558365	1.405777	-0.691993
	H	4.128331	-0.590909	-1.118864	4.113826	-0.086503	-1.204935
	H	3.208114	2.010521	-1.300984	2.817499	2.360593	-1.116195
	N	1.624947	1.210863	-0.069264	1.414433	1.218090	0.054881
	N	2.337300	-0.817562	0.068835	2.412177	-0.677536	-0.019247
	C	0.800178	2.408729	0.079521	0.413258	2.243181	0.325648
	H	-0.099946	2.326510	-0.530094	-0.413682	2.152050	-0.379108
	H	0.513763	2.523448	1.123453	0.035679	2.107934	1.337383
	H	1.381213	3.272094	-0.239294	0.882672	3.220124	0.229687

	C	2.485764	-2.235440	0.397371	2.739118	-2.085978	0.185247
	H	3.373255	-2.381432	1.014916	2.942671	-2.273817	1.237948
	H	1.606060	-2.559286	0.944338	1.906160	-2.706772	-0.137540
	H	2.583690	-2.813508	-0.522046	3.621972	-2.318421	-0.406226
	H	0.182095	-1.421629	1.894341	0.344887	-1.853978	1.542746
	H	0.044064	0.511609	2.288067	0.099068	-0.078436	2.413224
	B	0.045600	-0.320480	1.392743	0.114002	-0.677342	1.351098
	C	-1.347241	-0.249105	0.556902	-1.281513	-0.466274	0.552029
	C	-1.471715	-0.718740	-0.764438	-1.394605	-0.752062	-0.818167
	C	-2.523389	0.247118	1.149343	-2.437415	0.011354	1.184616
	C	-2.686966	-0.700365	-1.452692	-2.584830	-0.573835	-1.519487
	H	-0.595942	-1.112704	-1.274803	-0.522534	-1.121548	-1.355126
	C	-3.746768	0.267709	0.476566	-3.638223	0.191629	0.498755
	H	-2.478216	0.628778	2.165770	-2.393894	0.255815	2.242542
	C	-3.835517	-0.206336	-0.833213	-3.717188	-0.098555	-0.860807
	H	-2.738118	-1.071618	-2.471835	-2.631104	-0.803964	-2.578910
	H	-4.630593	0.658099	0.972106	-4.511748	0.564584	1.023547
	H	-4.782367	-0.188869	-1.362405	-4.646777	0.045123	-1.399817
**3**	C	-1.803075	0.991574	0.000091	-1.804163	0.980664	-0.000066
	C	-0.738299	1.828535	0.000064	-0.746650	1.823871	-0.000075
	N	0.399002	1.033883	-0.000015	0.390876	1.037339	0.000021
	N	-1.297154	-0.297007	0.000054	-1.286965	-0.298430	0.000038
	C	0.058486	-0.275311	-0.000032	0.061064	-0.267238	0.000059
	H	0.649698	-2.265381	-0.999079	0.638663	-2.252498	-0.999382
	B	0.953638	-1.629470	-0.000211	0.953955	-1.629218	0.000170
	C	1.757389	1.586276	-0.000146	1.742434	1.594569	0.000091
	H	2.466076	0.765704	-0.000240	2.453074	0.775641	0.000161
	H	1.898327	2.200663	-0.890275	1.876653	2.207860	-0.890393
	H	1.898533	2.200605	0.889991	1.876537	2.207925	0.890548
	C	-2.104755	-1.515703	-0.000045	-2.070691	-1.528009	-0.000016
	H	-1.884793	-2.108939	0.887510	-1.834973	-2.113089	0.888093
	H	-3.155777	-1.234849	0.000826	-3.125681	-1.264408	0.000388
	H	-1.886023	-2.108043	-0.888511	-1.835542	-2.112666	-0.888557
	H	-2.860136	1.193345	0.000173	-2.863581	1.170808	-0.000094
	H	-0.684301	2.903411	0.000125	-0.695132	2.899133	-0.000110
	F	2.378850	-1.331500	0.000287	2.364638	-1.324005	-0.000224

	H	0.649146	-2.266022	0.998081	0.639118	-2.252072	1.000137
**4**	C	-3.157320	0.594094	-0.834652	-3.133858	0.266143	-0.980094
	C	-2.799681	-0.618416	-1.323204	-2.522637	-0.852395	-1.441709
	N	-1.676249	-1.016004	-0.622447	-1.412655	-1.048341	-0.650968
	N	-2.246584	0.909673	0.159204	-2.378367	0.720680	0.081069
	C	-1.326917	-0.077019	0.288652	-1.318692	-0.083511	0.281819
	H	-0.196816	0.715469	2.165416	-0.345795	1.037740	2.070413
	B	-0.039630	-0.130556	1.304338	-0.103393	0.076539	1.368519
	C	-0.947195	-2.258977	-0.890955	-0.425808	-2.109189	-0.841657
	H	-0.346135	-2.507531	-0.022446	0.004926	-2.361538	0.123996
	H	-1.666554	-3.055529	-1.078201	-0.926948	-2.976998	-1.265985
	H	-0.306767	-2.133495	-1.765146	0.360697	-1.763169	-1.513167
	C	-2.292764	2.162672	0.916712	-2.694185	1.909121	0.873015
	H	-1.794220	2.958808	0.361736	-1.922836	2.664662	0.736141
	H	-3.335030	2.433139	1.079576	-3.650989	2.297271	0.531468
	H	-1.795288	2.020672	1.871226	-2.759487	1.642196	1.925206
	H	-3.973841	1.246381	-1.091343	-4.030771	0.766755	-1.301553
	H	-3.245323	-1.229349	-2.089101	-2.779948	-1.519051	-2.246726
	F	-0.036744	-1.453731	1.931940	-0.066803	-1.137835	2.158346
	C	1.342357	0.125709	0.493738	1.278223	0.263889	0.547556
	C	1.561666	1.297372	-0.252699	1.417183	1.247866	-0.441600
	C	2.394894	-0.802593	0.535459	2.384996	-0.557686	0.788174
	C	2.761647	1.532191	-0.924389	2.600645	1.408709	-1.157246
	H	0.779506	2.050541	-0.311235	0.577739	1.905331	-0.661399
	C	3.602474	-0.579192	-0.131748	3.577008	-0.407261	0.079821
	H	2.265181	-1.716918	1.105350	2.306754	-1.334917	1.542404
	C	3.791572	0.590715	-0.866757	3.689246	0.577237	-0.897840
	H	2.896144	2.448288	-1.491057	2.677769	2.179067	-1.917321
	H	4.396170	-1.317896	-0.076194	4.418192	-1.059900	0.288819
	H	4.727253	0.768757	-1.385969	4.613303	0.696452	-1.452137
**5**	C	-2.188910	0.103161	-0.447961	-2.158654	0.457420	-0.410315
	H	-3.164132	-0.223451	-0.764006	-3.184033	0.330435	-0.711370
	C	-1.649832	1.345380	-0.372005	-1.393144	1.569964	-0.304989
	H	-2.062881	2.309285	-0.613217	-1.617025	2.604702	-0.499565
	N	-0.363614	1.192312	0.111822	-0.154580	1.155508	0.135400
	C	0.583602	2.290600	0.306679	0.992998	2.028296	0.362608

	H	1.243849	2.047620	1.135939	1.518046	1.697182	1.256827
	H	1.176055	2.444510	-0.596112	1.669093	1.986153	-0.490952
	H	0.026946	3.197184	0.537109	0.631397	3.044312	0.502859
	N	-1.218348	-0.783070	-0.015120	-1.365233	-0.608001	-0.036923
	C	-1.407752	-2.234582	0.063388	-1.807766	-2.000591	-0.003215
	H	-2.376533	-2.472875	-0.370598	-2.830634	-2.038000	-0.369928
	H	-0.625778	-2.744223	-0.495982	-1.166852	-2.605649	-0.640815
	H	-1.384706	-2.562909	1.101563	-1.770029	-2.377895	1.016817
	C	-0.093218	-0.115690	0.330548	-0.134763	-0.180141	0.298029
	H	1.602893	-0.110623	1.898049	1.426299	-0.668008	1.917560
	H	1.115317	-1.897692	1.191810	0.819878	-2.224575	0.831790
	B	1.275036	-0.735593	0.912071	1.109255	-1.057795	0.817412
	C	2.436216	-0.619911	-0.163116	2.336407	-0.827716	-0.172044
	N	3.295270	-0.541042	-0.938147	3.226544	-0.653659	-0.887152
**6**	C	-1.493787	0.671197	0.100821	-1.658381	0.290471	0.127251
	C	-1.479651	-0.692125	0.097535	-1.198600	-0.984274	0.021708
	N	-0.186574	-1.072636	-0.260666	0.147125	-0.886751	-0.305083
	C	0.254770	-2.464267	-0.336966	1.033894	-2.030511	-0.478241
	H	1.253654	-2.491009	-0.760358	1.884883	-1.726624	-1.082400
	H	0.271798	-2.907321	0.660131	1.385982	-2.385710	0.491071
	H	-0.423392	-3.032582	-0.973593	0.494817	-2.827021	-0.988030
	N	-0.208400	1.080148	-0.254575	-0.574368	1.122861	-0.129213
	C	0.203655	2.481137	-0.323242	-0.676737	2.577088	-0.125175
	H	1.206402	2.530243	-0.735225	0.317930	2.998505	-0.220582
	H	-0.479219	3.036616	-0.966203	-1.295737	2.906631	-0.960090
	H	0.200101	2.921902	0.674984	-1.124608	2.905472	0.812080
	C	0.592068	0.012362	-0.476177	0.529613	0.400909	-0.393193
	H	2.401028	-0.946939	-1.580170	2.341728	0.427012	-1.819629
	H	2.393136	1.023109	-1.549509	2.089597	2.102534	-0.788450
	B	2.147864	0.027100	-0.910810	2.018572	0.904046	-0.756471
	C	3.077200	0.010560	0.376606	3.016644	0.354083	0.358617
	N	3.765589	-0.001137	1.310083	3.741992	-0.053885	1.159723
	C	-2.591906	1.634559	0.398382	-3.010572	0.824335	0.449640
	H	-2.334633	2.295830	1.230935	-3.002881	1.388856	1.385061
	H	-2.823306	2.263407	-0.466518	-3.375034	1.484140	-0.340989
	H	-3.499576	1.095700	0.668700	-3.717534	0.003334	0.556708

	C	-2.556589	-1.679865	0.391966	-1.884797	-2.294377	0.194870
	H	-2.791796	-2.294941	-0.481880	-1.907432	-2.859352	-0.740072
	H	-2.274255	-2.353336	1.206334	-1.382002	-2.904723	0.948531
	H	-3.468128	-1.162156	0.688969	-2.912194	-2.135198	0.518303
**7**	C	-3.223967	-1.058763	0.361779	-3.131642	-1.073247	0.464480
	H	-4.110053	-1.633612	0.156311	-3.980820	-1.721707	0.335944
	C	-2.724670	-0.583411	1.530032	-2.657231	-0.432550	1.560303
	H	-3.093346	-0.660788	2.538009	-3.012842	-0.407868	2.575856
	N	-1.551594	0.078315	1.222147	-1.534538	0.257075	1.159172
	C	-0.721542	0.782304	2.202263	-0.718568	1.105153	2.025195
	H	0.326240	0.673071	1.932406	0.333880	0.926722	1.808933
	H	-0.984552	1.840279	2.227145	-0.955512	2.153739	1.848768
	H	-0.891472	0.339777	3.181876	-0.931648	0.843492	3.059144
	N	-2.346753	-0.673655	-0.635936	-2.287938	-0.753600	-0.578423
	C	-2.543274	-0.982167	-2.056546	-2.439831	-1.249610	-1.945838
	H	-3.428949	-1.607639	-2.144552	-3.321950	-1.884644	-1.974993
	H	-2.689670	-0.064619	-2.624235	-2.565133	-0.413784	-2.630638
	H	-1.682094	-1.518567	-2.449449	-1.562887	-1.828039	-2.230721
	C	-1.316251	0.029450	-0.110089	-1.308190	0.064463	-0.153174
	H	-0.233646	0.448090	-2.090865	-0.255295	0.379298	-2.175564
	B	-0.059674	0.671784	-0.913967	-0.069014	0.646599	-1.013851
	C	-0.124139	2.255729	-0.750095	-0.082238	2.237811	-0.877224
	N	-0.180262	3.409407	-0.652575	-0.086706	3.387969	-0.776940
	C	1.364556	0.043311	-0.439492	1.321822	0.009494	-0.478499
	C	1.519122	-1.350436	-0.322762	1.403622	-1.365554	-0.214427
	C	2.502703	0.825983	-0.186102	2.484430	0.764180	-0.283018
	C	2.737875	-1.932890	0.024494	2.584567	-1.962338	0.217902
	H	0.669101	-2.001563	-0.509551	0.522046	-1.988954	-0.347919
	C	3.729743	0.254144	0.160017	3.673716	0.177895	0.148765
	H	2.434365	1.907014	-0.259423	2.462746	1.834294	-0.467556
	C	3.853619	-1.130012	0.267920	3.728808	-1.189146	0.401613
	H	2.818391	-3.012390	0.103431	2.613127	-3.028838	0.413069
	H	4.587759	0.891935	0.347031	4.557043	0.791034	0.291190
	H	4.803897	-1.577834	0.537743	4.650987	-1.647151	0.740610
**8**	C	0.000080	0.164007	0.380998	-0.113433	0.248970	0.249813
	C	-0.676919	-1.704979	-0.665415	-1.034008	-1.599713	-0.609986

	H	-1.371539	-2.421610	-1.066037	-1.815534	-2.240129	-0.977514
	N	-1.081213	-0.548213	-0.021820	-1.268715	-0.318592	-0.151776
	N	1.081576	-0.548028	-0.021594	0.846164	-0.671046	0.032114
	C	-2.500584	-0.156031	0.169655	-2.602081	0.313792	-0.034997
	H	-2.456591	0.803325	0.680675	-2.433814	1.373879	-0.219050
	C	2.500840	-0.155478	0.169905	2.287356	-0.475505	0.286132
	H	2.456577	0.803388	0.681827	2.359695	0.478965	0.806144
	H	-0.984372	1.625589	1.898317	-0.945483	2.284925	1.028509
	H	0.984386	1.626323	1.897743	0.659019	1.587474	1.963442
	B	-0.000170	1.558482	1.201034	0.102922	1.712786	0.897540
	C	0.677613	-1.704861	-0.665276	0.297822	-1.817634	-0.501850
	H	1.372443	-2.421355	-1.065776	0.888237	-2.678160	-0.765662
	C	3.218111	-1.163842	1.070072	2.813564	-1.579546	1.195251
	H	2.704774	-1.266700	2.028485	2.236735	-1.628695	2.120192
	H	3.282306	-2.149609	0.601689	2.775157	-2.554083	0.702883
	H	4.236072	-0.817584	1.261890	3.855058	-1.371332	1.444664
	C	3.189312	0.036850	-1.183357	3.034930	-0.391068	-1.039310
	H	2.648830	0.759561	-1.798001	2.636349	0.417380	-1.654087
	H	4.201556	0.413900	-1.022007	4.091820	-0.196636	-0.850045
	H	3.266679	-0.902912	-1.736601	2.952804	-1.332196	-1.589131
	C	-3.189450	0.034775	-1.183616	-3.551913	-0.227702	-1.093994
	H	-3.266621	-0.905530	-1.735959	-3.827787	-1.266021	-0.896645
	H	-4.201791	0.411598	-1.022361	-4.466948	0.365300	-1.073112
	H	-2.649387	0.757111	-1.799068	-3.118250	-0.155693	-2.092848
	C	-3.217238	-1.163866	1.070909	-3.135036	0.115568	1.378952
	H	-4.235314	-0.817884	1.262629	-4.103060	0.609235	1.481676
	H	-3.281070	-2.150088	0.603431	-3.264723	-0.949567	1.587442
	H	-2.703650	-1.265612	2.029306	-2.447082	0.538211	2.113171
	C	-0.000922	2.792033	0.202420	1.045310	2.560730	-0.068160
	N	-0.001434	3.703960	-0.514176	1.736571	3.170475	-0.764457
**9**	C	-2.412212	0.049366	0.344967	-2.391197	0.330404	0.327500
	C	-1.896391	1.304644	0.367168	-1.722846	1.506193	0.225325
	N	-0.560912	1.193306	0.035624	-0.415735	1.191385	-0.066942
	N	-1.381279	-0.802330	-0.003723	-1.473738	-0.670227	0.094581
	C	-0.240471	-0.100467	-0.188762	-0.262053	-0.141078	-0.144058
	H	1.108583	-1.849800	-0.922957	0.927276	-2.116565	-0.470141

	B	1.220628	-0.693624	-0.595656	1.124338	-0.929445	-0.464753
	C	0.377198	2.320912	0.008766	0.670057	2.161656	-0.203213
	H	1.174847	2.102893	-0.695158	1.412080	1.761715	-0.889608
	H	-0.159124	3.212566	-0.310878	0.260181	3.086788	-0.602494
	H	0.792440	2.481978	1.004463	1.123840	2.343832	0.770726
	C	-1.527084	-2.257693	-0.122414	-1.786394	-2.100079	0.106528
	H	-1.057800	-2.755213	0.725956	-1.263409	-2.588101	0.926511
	H	-2.589313	-2.491927	-0.137322	-2.859951	-2.206918	0.241495
	H	-1.066420	-2.600247	-1.045629	-1.489311	-2.548244	-0.838755
	H	-3.410317	-0.305237	0.533858	-3.426366	0.120882	0.534725
	H	-2.358212	2.253248	0.579240	-2.059743	2.523439	0.326906
	F	1.785135	0.102481	-1.658954	1.655097	-0.450333	-1.701956
	C	2.181684	-0.632408	0.690840	2.153787	-0.580636	0.730374
	N	2.870708	-0.620775	1.621514	2.873719	-0.340496	1.599113
**10**	C	-2.589287	0.204029	-0.000042	-2.571000	0.288534	-0.000448
	H	-3.629662	-0.070130	-0.000046	-3.622357	0.058147	-0.000121
	C	-1.991592	1.422928	-0.000155	-1.919181	1.477826	-0.002604
	H	-2.411132	2.413574	-0.000278	-2.289193	2.488324	-0.004511
	N	-0.629441	1.200485	-0.000192	-0.573784	1.191394	-0.001914
	C	0.386383	2.258883	-0.000181	0.499144	2.185770	-0.003526
	H	1.007787	2.178845	0.890669	1.111551	2.065383	0.889024
	H	1.008160	2.178498	-0.890741	1.113073	2.060809	-0.894399
	H	-0.122227	3.219898	-0.000487	0.045238	3.173507	-0.006468
	N	-1.579706	-0.738678	0.000024	-1.606193	-0.694100	0.001510
	C	-1.810879	-2.188375	0.000007	-1.884927	-2.131056	0.004158
	H	-2.885187	-2.356530	0.000110	-2.963843	-2.261794	0.004376
	H	-1.374724	-2.637594	-0.890270	-1.460183	-2.590178	-0.885974
	H	-1.374571	-2.637644	0.890186	-1.460233	-2.586881	0.896007
	C	-0.374959	-0.127268	-0.000069	-0.383486	-0.138993	0.000587
	H	0.912638	-2.044183	0.000113	0.849371	-2.096798	0.005239
	B	1.070507	-0.852897	0.000041	1.030212	-0.913231	0.002029
	C	1.889227	-0.469025	1.306609	1.854871	-0.508410	1.301908
	N	2.470358	-0.214359	2.274258	2.436150	-0.220902	2.254699
	C	1.889532	-0.469249	-1.306362	1.853644	-0.515293	-1.300761
	N	2.471050	-0.214845	-2.273847	2.433989	-0.232769	-2.255610
$Me^{\bullet}$	C	0.000001	0.000012	0.000000	-0.000004	0.000026	0.000000

	H	-0.929968	0.551226	0.000001	-0.863535	-0.647449	0.000000
	H	0.942408	0.529675	0.000001	0.992477	-0.424156	0.000000
	H	-0.012445	-1.080974	0.000001	-0.128918	1.071450	0.000000
Et•	C	-0.009829	-0.693509	0.000000	-0.012056	-0.694074	0.000000
	H	-0.504602	-1.100647	0.887174	-0.508136	-1.096382	0.885841
	H	-0.504602	-1.100647	-0.887174	-0.508136	-1.096382	-0.885841
	H	1.013692	-1.102986	0.000000	1.009622	-1.100061	0.000000
	C	-0.009829	0.794059	0.000000	-0.012056	0.794107	0.000000
	H	0.056732	1.350489	-0.927230	0.075662	1.346314	-0.925970
	H	0.056732	1.350489	0.927230	0.075662	1.346314	0.925970
•CH_2CN	C	-0.007816	-1.198574	0.000000	-0.001686	-1.197091	0.000000
	H	-0.011665	-1.750233	0.932909	-0.002471	-1.732025	0.937979
	H	-0.011665	-1.750233	-0.932909	-0.002471	-1.732025	-0.937979
	C	0.000000	0.182266	0.000000	0.000000	0.194309	0.000000
	N	0.010032	1.371188	0.000000	0.002151	1.354392	0.000000
CH_4	C	0.000000	0.000000	0.000000	0.000000	0.000000	0.000000
	H	0.629939	0.629939	0.629939	0.628958	0.628958	0.628958
	H	-0.629939	-0.629939	0.629939	-0.628958	-0.628958	0.628958
	H	-0.629939	0.629939	-0.629939	-0.628958	0.628958	-0.628958
	H	0.629939	-0.629939	-0.629939	0.628958	-0.628958	-0.628958
CH_3CH_3	C	0.765699	-0.000030	0.000000	-0.763677	0.000000	0.000032
	H	1.162380	0.509667	-0.882618	-1.158301	-0.881760	0.508942
	H	1.162380	0.509665	0.882619	-1.158301	0.881748	0.508964
	H	1.162407	-1.019202	-0.000001	-1.158285	0.000013	-1.018048
	C	-0.765703	0.000005	0.000000	0.763678	0.000000	-0.000033
	H	-1.162399	-0.509603	0.882644	1.158303	0.881762	-0.508938
	H	-1.162347	1.019220	0.000001	1.158280	-0.000013	1.018049
	H	-1.162399	-0.509602	-0.882645	1.158303	-0.881749	-0.508960
CH_3CN	C	0.000142	-1.175396	0.000000	-0.000060	-1.176832	0.000000
	H	0.513031	-1.548797	0.888061	0.512985	-1.542408	0.888693
	H	0.513031	-1.548797	-0.888061	0.512985	-1.542408	-0.888693
	C	0.000000	0.278793	0.000000	0.000000	0.280540	0.000000
	N	-0.000238	1.432320	0.000000	0.000067	1.429308	0.000000
	H	-1.025252	-1.549026	0.000000	-1.026077	-1.542586	0.000000
**R1**	C	0.679331	-1.508037	-0.000017	0.677910	-1.512118	0.000061
	H	1.375287	-2.329324	-0.000085	1.377474	-2.330420	0.000144

	C	-0.679348	-1.508045	0.000170	-0.677910	-1.512119	-0.000124
	H	-1.375319	-2.329319	0.000249	-1.377474	-2.330421	-0.000188
	N	-1.094593	-0.193022	-0.000075	-1.087346	-0.197155	-0.000047
	C	-2.477950	0.250004	-0.000035	-2.461275	0.263327	0.000112
	H	-2.686136	0.851541	0.887320	-2.653490	0.868132	0.887947
	H	-2.686003	0.852102	-0.887035	-2.653403	0.868821	-0.887267
	H	-3.126382	-0.624695	-0.000365	-3.119408	-0.603191	-0.000258
	N	1.094590	-0.193024	-0.000174	1.087346	-0.197155	-0.000114
	C	2.477952	0.249980	0.000045	2.461275	0.263326	0.000080
	H	3.126366	-0.624731	-0.001012	3.119407	-0.603192	-0.000750
	H	2.685896	0.852709	-0.886546	2.653290	0.869215	-0.887052
	H	2.686285	0.850882	0.887804	2.653606	0.867736	0.888162
	C	-0.000011	0.660972	-0.000027	-0.000001	0.647191	-0.000094
	B	0.000020	2.163537	0.000063	0.000001	2.155570	0.000012
	H	1.036756	2.758080	0.000127	1.041210	2.738995	0.000045
	H	-1.036677	2.758149	0.000159	-1.041206	2.739001	0.000084
**R2**	C	-2.971391	1.384497	-0.073463	2.866282	-1.437505	-0.065102
	H	-3.385314	2.374618	-0.156682	3.226345	-2.449121	-0.142483
	C	-3.575664	0.197206	0.178901	3.523178	-0.285791	0.204495
	H	-4.609660	-0.032777	0.370651	4.562195	-0.104112	0.420804
	N	-2.597574	-0.775315	0.162403	2.594696	0.730188	0.167836
	C	-2.836003	-2.188260	0.416146	2.877386	2.130804	0.423010
	H	-2.637723	-2.781214	-0.478701	2.750475	2.717065	-0.488172
	H	-2.188498	-2.540825	1.220591	2.195005	2.509380	1.184949
	H	-3.877250	-2.316539	0.707537	3.904124	2.218091	0.772354
	N	-1.621538	1.136385	-0.244485	1.538514	-1.117224	-0.267070
	C	-0.679618	2.146116	-0.709220	0.558862	-2.063095	-0.778254
	H	-1.218925	2.878506	-1.309930	1.074429	-2.790601	-1.403957
	H	-0.194700	2.656897	0.124606	0.043607	-2.581479	0.030936
	H	0.082657	1.670049	-1.323165	-0.172958	-1.522029	-1.375669
	C	-1.356624	-0.215225	-0.095000	1.345779	0.236368	-0.119811
	B	-0.062997	-1.011664	-0.232587	0.085857	1.087656	-0.275977
	H	-0.218778	-2.177040	-0.472696	0.263959	2.244584	-0.526312
	C	1.400092	-0.511381	-0.065891	-1.370585	0.569330	-0.078666
	C	2.456728	-1.217008	-0.696871	-2.452674	1.205551	-0.723289
	C	1.792980	0.569660	0.761968	-1.702078	-0.486833	0.795852

	C	3.791067	-0.852423	-0.546379	-3.767731	0.794979	-0.538791
	H	2.212777	-2.069787	-1.323628	-2.247829	2.038915	-1.388965
	C	3.126813	0.935241	0.924466	-3.014690	-0.901966	0.989607
	H	1.037571	1.117536	1.315320	-0.910418	-0.975832	1.356319
	C	4.138489	0.232560	0.264472	-4.058400	-0.267710	0.316502
	H	4.565790	-1.416531	-1.056348	-4.571442	1.305510	-1.058954
	H	3.381948	1.765519	1.575721	-3.228424	-1.715330	1.674962
	H	5.177652	0.516519	0.389450	-5.082800	-0.588874	0.465393
**R3**	C	-1.729070	1.053177	-0.000013	-1.732511	1.048705	-0.000012
	C	-0.616445	1.830875	0.000024	-0.624226	1.827252	-0.000023
	N	0.484134	0.994503	-0.000019	0.473569	0.991618	-0.000011
	N	-1.320047	-0.265760	-0.000048	-1.316378	-0.265558	-0.000011
	C	0.070201	-0.334555	-0.000044	0.063683	-0.328125	0.000007
	H	0.410270	-2.680840	-0.000025	0.414297	-2.670248	-0.000031
	B	0.891284	-1.590226	-0.000027	0.894236	-1.580426	0.000035
	C	1.868750	1.440357	0.000024	1.859653	1.420143	0.000039
	H	2.390845	1.076871	-0.886969	2.371723	1.046000	-0.887347
	H	1.881464	2.529237	-0.000111	1.883364	2.508122	-0.000072
	H	2.390739	1.077098	0.887175	2.371615	1.046179	0.887566
	C	-2.198477	-1.420784	0.000043	-2.170272	-1.433835	0.000020
	H	-2.025191	-2.034363	0.887823	-1.977035	-2.039736	0.888122
	H	-3.231202	-1.076050	0.000231	-3.208813	-1.109337	0.000121
	H	-2.025486	-2.034297	-0.887841	-1.977196	-2.039685	-0.888153
	H	-2.769870	1.328491	0.000007	-2.775598	1.316388	-0.000020
	H	-0.518357	2.902936	-0.000010	-0.523276	2.899201	-0.000048
	F	2.280223	-1.517064	0.000014	2.274604	-1.482446	-0.000038
**R4**	C	-3.543519	0.416444	0.237238	3.511832	-0.475833	0.221268
	C	-2.928761	1.579768	-0.085841	2.861528	-1.617711	-0.097155
	N	-1.587525	1.304016	-0.287967	1.530535	-1.299981	-0.290511
	N	-2.586260	-0.578796	0.234914	2.583520	0.542614	0.224090
	C	-1.343568	-0.045659	-0.077997	1.333911	0.048847	-0.080695
	B	-0.041306	-0.829229	-0.204523	0.059207	0.874259	-0.189502
	C	-0.654826	2.263203	-0.863027	0.566544	-2.218731	-0.872678
	H	0.039518	1.740318	-1.520348	-0.142817	-1.651190	-1.474444
	H	-1.218259	2.993094	-1.444074	1.100602	-2.922954	-1.509472
	H	-0.084296	2.785609	-0.092955	0.020127	-2.768972	-0.105750

	C	-2.834764	-1.950918	0.660287	2.850770	1.908425	0.641801
	H	-2.177884	-2.211095	1.493035	2.155507	2.189522	1.434907
	H	-3.870687	-2.025737	0.986805	3.870306	1.957585	1.018647
	H	-2.659926	-2.650136	-0.156512	2.732644	2.599045	-0.191901
	H	-4.575268	0.214767	0.468822	4.549898	-0.300144	0.447249
	H	-3.330443	2.570991	-0.206375	3.228619	-2.622479	-0.218285
	F	-0.202501	-2.192890	-0.492341	0.234968	2.229470	-0.454254
	C	1.418236	-0.343379	-0.025999	-1.399712	0.378508	-0.013132
	C	2.480548	-1.086342	-0.599027	-2.462772	1.099245	-0.593156
	C	1.787889	0.775129	0.758641	-1.741954	-0.740500	0.771382
	C	3.811757	-0.720056	-0.426748	-3.785669	0.705598	-0.429687
	H	2.247459	-1.963426	-1.193523	-2.238177	1.980088	-1.186175
	C	3.117982	1.144997	0.933382	-3.062445	-1.140416	0.936003
	H	1.020069	1.346591	1.268733	-0.957885	-1.286429	1.287088
	C	4.142002	0.403529	0.336780	-4.093471	-0.422525	0.330226
	H	4.597286	-1.310339	-0.887821	-4.581252	1.276937	-0.895642
	H	3.361159	2.005281	1.548795	-3.293822	-2.004181	1.549892
	H	5.178955	0.689400	0.474334	-5.124263	-0.731216	0.459567
**R5**	C	-2.242475	0.547016	-0.000036	-2.227791	0.588463	-0.000007
	H	-3.317803	0.505016	-0.000062	-3.303872	0.566964	-0.000012
	C	-1.401957	1.614553	-0.000016	-1.363148	1.632925	-0.000004
	H	-1.613120	2.669807	-0.000031	-1.543254	2.694073	-0.000006
	N	-0.113241	1.125099	0.000017	-0.090858	1.108934	0.000004
	C	1.083591	1.959591	0.000031	1.133590	1.895517	0.000006
	H	1.683781	1.762575	0.888380	1.722526	1.668562	0.888362
	H	1.683755	1.762652	-0.888353	1.722513	1.668590	-0.888365
	H	0.774206	3.002812	0.000082	0.862474	2.948771	0.000025
	N	-1.462853	-0.588682	-0.000017	-1.473349	-0.561292	-0.000003
	C	-1.981603	-1.951872	0.000004	-2.004791	-1.915175	0.000001
	H	-3.068779	-1.905835	-0.000066	-3.090647	-1.856373	-0.000029
	H	-1.643010	-2.486216	-0.888385	-1.666725	-2.447709	-0.888989
	H	-1.643119	-2.486149	0.888475	-1.666772	-2.447690	0.889020
	C	-0.127068	-0.251689	0.000023	-0.141199	-0.256223	0.000004
	H	0.769389	-2.410091	0.000066	0.722952	-2.435806	0.000018
	B	1.017477	-1.246466	0.000054	0.983426	-1.278334	0.000010
	C	2.487738	-0.852425	0.000021	2.460358	-0.869518	0.000002

	N	3.630088	-0.618316	-0.000075	3.590144	-0.608163	-0.000014
**R6**	C	1.732014	-0.233833	0.000017	-1.731662	-0.208198	-0.000017
	C	1.183309	1.019998	-0.000057	-1.160117	1.029432	0.000058
	N	-0.196847	0.859166	-0.000053	0.212880	0.838863	0.000058
	C	-1.170599	1.945079	-0.000115	1.211774	1.896027	0.000133
	H	-1.801220	1.888697	-0.887819	1.839134	1.818273	0.888201
	H	-1.801166	1.888851	0.887639	1.839126	1.818410	-0.887954
	H	-0.645391	2.896244	-0.000214	0.707926	2.858911	0.000213
	N	0.677023	-1.136520	0.000067	-0.695435	-1.127101	-0.000066
	C	0.806407	-2.589072	0.000149	-0.842646	-2.573546	-0.000145
	H	1.860410	-2.853320	0.000269	-1.900716	-2.821672	-0.000202
	H	0.333621	-3.011389	0.887903	-0.372887	-2.996020	-0.888710
	H	0.333799	-3.011481	-0.887658	-0.372960	-2.996112	0.888417
	C	-0.528248	-0.474518	0.000024	0.512222	-0.491870	-0.000020
	H	-1.940954	-2.338145	0.000103	1.907198	-2.378873	-0.000104
	B	-1.886405	-1.149246	0.000053	1.860734	-1.193014	-0.000053
	C	-3.216580	-0.409170	0.000018	3.190642	-0.431530	-0.000031
	N	-4.270834	0.090403	-0.000004	4.224429	0.094818	-0.000020
	C	3.163070	-0.654196	0.000048	-3.166319	-0.609292	-0.000054
	H	3.410549	-1.249493	0.884114	-3.414848	-1.200094	-0.884954
	H	3.410536	-1.249665	-0.883904	-3.414869	-1.200188	0.884776
	H	3.810707	0.221636	-0.000041	-3.797913	0.277357	-0.000016
	C	1.847906	2.355070	-0.000131	-1.787605	2.380459	0.000132
	H	1.580866	2.941823	-0.884207	-1.501008	2.953577	0.885104
	H	1.580868	2.941919	0.883883	-1.501002	2.953675	-0.884775
	H	2.930341	2.233057	-0.000125	-2.871715	2.282876	0.000123
**R7**	C	-2.793797	-1.790186	-0.272639	-2.722807	-1.842382	-0.271821
	H	-3.149045	-2.762296	-0.567533	-3.037528	-2.831543	-0.556530
	C	-3.425898	-0.775278	0.367380	-3.398682	-0.835272	0.329787
	H	-4.430918	-0.702684	0.745086	-4.414037	-0.779326	0.682434
	N	-2.516900	0.255735	0.493776	-2.522949	0.219864	0.453756
	C	-2.799180	1.516237	1.178212	-2.843932	1.490430	1.093194
	H	-1.936107	1.803939	1.777037	-2.013254	1.787819	1.732291
	H	-3.015752	2.307834	0.460421	-3.015768	2.263418	0.344773
	H	-3.658721	1.369634	1.829863	-3.739425	1.352479	1.695168
	N	-1.506037	-1.367178	-0.537946	-1.445390	-1.385253	-0.513838

	C	-0.550049	-2.139831	-1.329586	-0.449916	-2.133373	-1.273703
	H	-1.105229	-2.773231	-2.019736	-0.972511	-2.770752	-1.984362
	H	0.082018	-1.456110	-1.892628	0.185665	-1.430034	-1.808578
	H	0.077308	-2.760426	-0.689326	0.165869	-2.743748	-0.614033
	C	-1.318110	-0.095641	-0.065421	-1.310770	-0.108177	-0.065303
	B	-0.041014	0.782198	-0.179743	-0.056873	0.799111	-0.156343
	C	-0.259017	2.257966	-0.525468	-0.303870	2.282814	-0.475707
	N	-0.410269	3.378906	-0.809923	-0.479956	3.400539	-0.731814
	C	1.406486	0.257310	0.028897	1.394224	0.285018	0.043679
	C	2.528803	0.969049	-0.462197	2.502598	0.998934	-0.455640
	C	1.687421	-0.916038	0.770514	1.673815	-0.888744	0.773045
	C	3.831447	0.531440	-0.246876	3.805110	0.557089	-0.260448
	H	2.369296	1.881738	-1.027440	2.332705	1.916712	-1.010615
	C	2.989320	-1.356361	0.990927	2.974785	-1.335672	0.970923
	H	0.868975	-1.480412	1.206393	0.854362	-1.448115	1.216295
	C	4.072902	-0.638201	0.479468	4.049907	-0.617587	0.450037
	H	4.663756	1.102635	-0.645143	4.634158	1.128426	-0.663364
	H	3.162107	-2.257171	1.570927	3.154203	-2.241103	1.540335
	H	5.087861	-0.979466	0.650168	5.065226	-0.964281	0.602904
**R8**	C	0.116851	0.346751	-0.116414	0.131811	0.279287	-0.221452
	C	-0.117516	-1.893398	0.073932	-0.071501	-1.941265	0.072803
	H	-0.627292	-2.836936	0.152950	-0.569088	-2.887664	0.186248
	N	-0.786978	-0.691738	-0.032205	-0.752501	-0.757946	-0.102206
	N	1.357514	-0.261167	-0.084310	1.372586	-0.297028	-0.147365
	C	-2.262818	-0.573742	-0.002478	-2.219298	-0.611126	-0.070846
	H	-2.477891	0.458590	-0.263381	-2.449887	0.188756	-0.773789
	C	2.664432	0.436178	-0.099374	2.642961	0.450856	-0.172301
	H	2.454762	1.426210	-0.498037	2.490386	1.262458	-0.884003
	H	0.829652	2.578794	-0.250096	0.796490	2.523170	-0.465846
	B	-0.109867	1.850797	-0.204496	-0.120992	1.776634	-0.379636
	C	1.211280	-1.626242	0.043207	1.252036	-1.654354	0.044118
	H	2.048049	-2.299677	0.092182	2.100572	-2.309601	0.126861
	C	3.208905	0.580115	1.325269	2.931990	1.025582	1.211114
	H	2.492374	1.099625	1.965052	2.111013	1.661043	1.546760
	H	3.423905	-0.397393	1.766087	3.069046	0.215712	1.932340
	H	4.136461	1.157339	1.309872	3.845889	1.621765	1.181606

	C	3.646813	-0.261114	-1.042976	3.781182	-0.427457	-0.674646
	H	3.220546	-0.374039	-2.042138	3.530784	-0.900749	-1.625564
	H	4.551582	0.344950	-1.124062	4.662351	0.197923	-0.822693
	H	3.943537	-1.246585	-0.675286	4.043765	-1.200007	0.051513
	C	-2.901673	-1.476007	-1.061736	-2.904705	-1.884262	-0.549291
	H	-2.758855	-2.536354	-0.838067	-2.796521	-2.696473	0.173080
	H	-3.976750	-1.286132	-1.092311	-3.970847	-1.682818	-0.659594
	H	-2.489538	-1.269689	-2.051815	-2.514138	-2.209894	-1.514983
	C	-2.792280	-0.843805	1.409312	-2.669957	-0.210661	1.330861
	H	-3.872601	-0.683695	1.433117	-3.744527	-0.019031	1.331554
	H	-2.595451	-1.874175	1.718481	-2.458064	-1.017493	2.037499
	H	-2.330675	-0.169674	2.133900	-2.158357	0.694194	1.661832
	C	-1.465156	2.538691	-0.226363	-1.518098	2.401442	-0.419836
	N	-2.467037	3.137232	-0.244663	-2.551862	2.927441	-0.448684
**R9**	C	-1.733642	-1.634838	0.000063	1.698384	1.660784	-0.000080
	C	-2.413466	-0.460741	-0.000009	2.400552	0.502947	-0.000266
	N	-1.483871	0.558544	-0.000008	1.490282	-0.530112	-0.000128
	N	-0.387229	-1.335808	0.000155	0.361631	1.330734	0.000164
	C	-0.208061	0.031783	0.000074	0.214945	-0.030622	0.000120
	B	1.087379	0.817387	0.000109	-1.061166	-0.846713	0.000231
	C	-1.831204	1.978285	-0.000274	1.842984	-1.944246	-0.000286
	H	-1.432979	2.468962	-0.887894	1.441517	-2.430680	-0.888129
	H	-2.916477	2.059057	-0.000962	2.928013	-2.019430	-0.001629
	H	-1.434083	2.469066	0.887797	1.443750	-2.430397	0.888727
	C	0.673518	-2.336368	-0.000040	-0.730385	2.290581	0.000422
	H	1.296969	-2.231811	0.888490	-1.346820	2.158210	0.889856
	H	0.212563	-3.322093	0.000667	-0.303112	3.290752	0.000901
	H	1.295960	-2.232587	-0.889382	-1.346669	2.158948	-0.889230
	H	-2.091330	-2.649678	0.000068	2.030948	2.684569	-0.000085
	H	-3.471869	-0.266805	0.000065	3.462009	0.325227	-0.000436
	F	1.037720	2.203181	0.000298	-0.987908	-2.221073	0.000664
	C	2.486219	0.205264	-0.000058	-2.469759	-0.231431	-0.000277
	N	3.574626	-0.209875	-0.000235	-3.545194	0.199085	-0.000737
**R10**	C	2.484841	0.678490	0.017732	2.477387	0.677247	-0.000052
	C	2.484722	-0.678869	-0.017708	2.477118	-0.678027	-0.000135
	N	1.171418	-1.088539	-0.028948	1.164990	-1.082801	0.000017

	N	1.171591	1.088379	0.028922	1.165419	1.082563	-0.000054
	C	0.336485	0.000009	0.000001	0.340193	0.000036	-0.000079
	B	-1.196448	0.000057	0.000037	-1.192952	0.000208	-0.000208
	C	0.771224	-2.495599	-0.079749	0.747465	-2.481731	-0.000176
	H	0.152753	-2.681163	-0.956400	0.157998	-2.696025	-0.890069
	H	1.674623	-3.097999	-0.144874	1.644432	-3.096365	-0.000497
	H	0.220533	-2.768188	0.819303	0.158328	-2.696409	0.889869
	C	0.771651	2.495526	0.079726	0.748540	2.481686	0.000522
	H	0.153597	2.681308	0.956624	0.158746	2.695875	0.890219
	H	1.675183	3.097781	0.144323	1.645805	3.095885	0.001601
	H	0.220594	2.768063	-0.819114	0.159958	2.697051	-0.889721
	H	3.301427	1.378679	0.035864	3.291031	1.381246	-0.000388
	H	3.301199	-1.379192	-0.035773	3.290477	-1.382353	0.000107
	C	-2.025843	1.281815	-0.062917	-2.015018	1.294190	-0.000284
	C	-2.026050	-1.281552	0.062936	-2.015181	-1.293666	0.000149
	N	-2.707025	2.222837	-0.115502	-2.672664	2.246087	-0.000382
	N	-2.707391	-2.222462	0.115490	-2.672751	-2.245616	0.000454
**TS1**	C	0.029081	-0.000012	0.464158	0.089368	-0.167365	0.484965
	C	1.898055	-0.677801	-0.608419	1.888399	0.583199	-0.634886
	C	1.897980	0.677978	-0.608421	1.021028	1.616454	-0.519089
	H	2.606270	-1.381272	-1.010308	2.858935	0.522312	-1.096358
	H	2.606121	1.381525	-1.010307	1.085794	2.635435	-0.859827
	N	0.749140	1.080008	0.051936	-0.076252	1.136434	0.166729
	N	0.749262	-1.079955	0.051947	1.300586	-0.501510	-0.013348
	C	0.357104	2.471172	0.262383	-1.265114	1.904650	0.509212
	H	1.147180	3.113662	-0.121796	-1.237714	2.846126	-0.035114
	H	-0.572808	2.687164	-0.264187	-2.150785	1.337857	0.225004
	H	0.215902	2.663347	1.325281	-1.291214	2.098113	1.581542
	C	0.357353	-2.471161	0.262345	1.887958	-1.832776	0.071978
	H	1.147632	-3.113561	-0.121566	2.896132	-1.787398	-0.333874
	H	0.215860	-2.663296	1.325209	1.923073	-2.153353	1.111462
	H	-0.572376	-2.687316	-0.264487	1.292079	-2.540759	-0.502892
	H	-1.577687	-1.014848	1.801988	-0.554141	-2.184738	1.453702
	H	-1.577768	1.014626	1.802042	-1.561855	-0.524175	2.072572
	B	-1.377614	-0.000086	1.173309	-0.988170	-1.095017	1.173734
	H	-2.269180	-0.000094	0.196520	-1.882980	-1.223182	0.205431

	C	-3.527272	-0.000103	-0.941288	-2.926913	-1.182311	-1.081265
	H	-4.390366	-0.001308	-0.284911	-2.968969	-2.228670	-1.362361
	H	-3.327373	0.922681	-1.475099	-3.824205	-0.769985	-0.633029
	H	-3.325964	-0.921682	-1.476650	-2.381998	-0.528073	-1.754089
**TS2**	C	-1.332283	0.026391	-0.186873	-1.344193	0.067521	-0.210412
	C	-3.371959	-0.917532	0.020588	-3.362013	-0.846356	0.164519
	C	-2.899414	-0.692279	1.270905	-2.786864	-0.621204	1.369177
	H	-4.300028	-1.347711	-0.313580	-4.318764	-1.265559	-0.095163
	H	-3.335699	-0.889440	2.234840	-3.141801	-0.804729	2.368716
	N	-1.652347	-0.110447	1.128703	-1.553508	-0.055938	1.119770
	N	-2.400489	-0.477157	-0.862139	-2.462047	-0.420100	-0.791236
	C	-0.807164	0.298568	2.248687	-0.604454	0.343328	2.152696
	H	-0.225894	-0.546978	2.619299	0.006480	-0.508521	2.452745
	H	-0.130940	1.079898	1.912009	0.038856	1.122841	1.750568
	H	-1.440662	0.683326	3.047285	-1.159895	0.723900	3.008494
	C	-2.535817	-0.531569	-2.318023	-2.692238	-0.481823	-2.230748
	H	-2.787634	0.451931	-2.716413	-2.810529	0.521949	-2.635886
	H	-1.601596	-0.867659	-2.761396	-1.848478	-0.969259	-2.714467
	H	-3.329518	-1.235403	-2.562996	-3.599191	-1.056603	-2.404998
	H	-0.172178	0.894625	-1.987723	-0.245904	0.838332	-2.104160
	B	-0.036333	0.725000	-0.795220	-0.073975	0.722758	-0.913038
	H	-0.066598	1.955735	-0.298174	-0.044496	1.947570	-0.425732
	C	-0.047412	3.561051	0.223578	0.192511	3.436381	0.274005
	H	0.158955	4.072582	-0.710149	0.210261	4.079226	-0.598796
	H	0.756739	3.549424	0.951551	1.142910	3.276355	0.771888
	C	1.382596	0.065684	-0.424056	1.323361	0.039082	-0.516505
	C	2.577894	0.767737	-0.685889	2.530289	0.724443	-0.737981
	C	1.532954	-1.221786	0.126151	1.430439	-1.242603	0.045644
	C	3.834373	0.227046	-0.417703	3.766703	0.168452	-0.423645
	H	2.518480	1.766015	-1.111068	2.493014	1.723250	-1.165444
	C	2.786253	-1.776428	0.399731	2.662081	-1.811678	0.368076
	H	0.649535	-1.818163	0.338822	0.526971	-1.818751	0.235147
	C	3.947455	-1.053427	0.130374	3.839358	-1.107050	0.134935
	H	4.728616	0.803278	-0.635200	4.676730	0.728727	-0.611551
	H	2.854653	-2.775180	0.820276	2.703289	-2.806613	0.799135
	H	4.923082	-1.478362	0.340737	4.799856	-1.544020	0.383383

	H	-1.052656	3.669574	0.617203	-0.680430	3.519428	0.913010
**TS3**	C	2.192642	0.732666	0.378894	1.660042	1.503837	0.265263
	C	2.145282	-0.602847	0.601078	2.184143	0.287962	0.547290
	N	0.900788	-1.040252	0.175266	1.245257	-0.652019	0.165200
	N	0.980732	1.090233	-0.185065	0.411541	1.279366	-0.277621
	C	0.176660	-0.000122	-0.314417	0.154241	-0.043584	-0.341878
	H	-1.483489	0.890047	-1.691368	-1.630663	-0.169352	-1.844357
	B	-1.335348	0.065950	-0.814748	-1.250604	-0.648756	-0.799044
	H	-2.021919	0.510606	0.236554	-2.062758	-0.183059	0.158730
	C	-3.092754	0.857714	1.462303	-2.964680	0.341967	1.402236
	H	-3.972424	0.312915	1.136698	-3.337995	-0.588612	1.816376
	H	-2.574463	0.456485	2.327163	-2.223252	0.861294	2.001975
	H	-3.148948	1.939394	1.397182	-3.702982	0.970702	0.915027
	C	0.474484	-2.439618	0.252789	1.407994	-2.094697	0.335022
	H	-0.577585	-2.502276	-0.002854	0.816392	-2.440051	1.182126
	H	1.055087	-3.042868	-0.447300	1.079395	-2.605181	-0.565190
	H	0.635096	-2.807279	1.266684	2.461484	-2.296206	0.516222
	C	0.609240	2.452397	-0.562952	-0.516529	2.305285	-0.736407
	H	-0.234118	2.794903	0.037151	-1.506160	2.098618	-0.329813
	H	1.463835	3.102885	-0.388073	-0.166084	3.271806	-0.381424
	H	0.334463	2.486567	-1.616740	-0.564988	2.306600	-1.825351
	H	2.970803	1.451473	0.568371	2.058707	2.495001	0.396702
	H	2.876004	-1.274310	1.017615	3.130739	0.007567	0.976146
	F	-1.872173	-1.222144	-1.178324	-1.249741	-2.075709	-0.784907
**TS4**	C	-3.108026	-0.070901	-1.317370	2.833337	-1.266975	1.027411
	H	-3.736917	0.276205	-2.118776	3.229612	-1.860358	1.833274
	C	-3.234409	-1.132209	-0.484480	3.341861	-0.962249	-0.190188
	H	-3.997941	-1.887695	-0.417504	4.271749	-1.236550	-0.657648
	N	-2.134788	-1.119573	0.355056	2.409870	-0.165385	-0.824808
	N	-1.937291	0.577004	-0.964643	1.607015	-0.642151	1.111410
	C	-1.332291	-0.064211	0.066547	1.342386	0.032972	-0.026650
	B	0.020629	0.370830	0.837077	0.036026	0.934326	-0.278937
	C	1.396464	-0.066066	0.146124	-1.339395	0.112051	-0.294847
	C	1.543696	-0.324331	-1.229896	-1.420549	-1.286514	-0.335262
	C	2.563680	-0.160020	0.931035	-2.553148	0.817603	-0.262192
	C	2.778494	-0.647967	-1.794641	-2.645539	-1.953002	-0.339034

	H	0.676203	-0.286308	-1.882445	-0.506339	-1.875397	-0.370736
	C	3.801022	-0.487528	0.377894	-3.782218	0.165887	-0.274570
	H	2.495491	0.023228	1.998698	-2.528841	1.903428	-0.221415
	C	3.917085	-0.730638	-0.992373	-3.832456	-1.227191	-0.309334
	H	2.850538	-0.844254	-2.859843	-2.673709	-3.036901	-0.370448
	H	4.677499	-0.553366	1.015096	-4.702433	0.739786	-0.251074
	H	4.878060	-0.984782	-1.426435	-4.787350	-1.740445	-0.313507
	H	0.004869	1.708454	0.905669	-0.023639	1.743969	0.774543
	C	0.073015	3.282859	1.389772	-0.332880	2.910027	1.871596
	H	0.735669	3.724305	0.651709	-1.192917	2.495159	2.387452
	H	-0.965416	3.596272	1.343962	0.591259	2.948807	2.439488
	H	0.486686	3.167266	2.385948	-0.531519	3.762606	1.231232
	C	-1.895418	-2.127701	1.392602	2.565299	0.366457	-2.178902
	H	-0.830698	-2.328844	1.466911	1.677998	0.136728	-2.763517
	H	-2.261459	-1.776110	2.356779	2.704307	1.444720	-2.143770
	H	-2.420867	-3.038137	1.109130	3.435898	-0.107792	-2.626215
	C	-1.442016	1.774543	-1.645264	0.709090	-0.717401	2.259659
	H	-2.255214	2.494326	-1.739191	1.300746	-0.941420	3.144846
	H	-0.640072	2.208690	-1.056724	0.210617	0.242849	2.380277
	H	-1.070682	1.515682	-2.637715	-0.038498	-1.495357	2.103911
	F	-0.065792	-0.060676	2.216307	0.216668	1.767477	-1.430482
**TS5**	C	0.345087	-0.103218	-0.167770	0.363681	-0.190034	-0.100136
	C	1.908754	1.387754	0.462658	1.722046	1.585062	0.048035
	C	2.549008	0.239774	0.130124	2.497600	0.474822	0.037885
	H	2.290596	2.332087	0.809603	1.979620	2.628599	0.104734
	H	3.595864	-0.008786	0.133891	3.566362	0.357837	0.087529
	N	1.577403	-0.666500	-0.255716	1.645131	-0.606103	-0.054414
	N	0.556802	1.160609	0.280819	0.414231	1.155679	-0.030473
	C	1.858421	-2.038608	-0.682597	2.071866	-2.002167	-0.087121
	H	1.460016	-2.750859	0.039422	1.754378	-2.511622	0.821325
	H	1.411295	-2.225073	-1.657357	1.637363	-2.496345	-0.953490
	H	2.937732	-2.158653	-0.749436	3.156688	-2.022585	-0.158561
	C	-0.488833	2.151434	0.537895	-0.756287	2.027259	-0.045429
	H	-0.832724	2.596974	-0.395630	-1.119446	2.154661	-1.064759
	H	-1.328022	1.673608	1.039680	-1.539848	1.578468	0.563352
	H	-0.078229	2.926109	1.182460	-0.472560	2.990285	0.373167

	H	-0.895116	-1.914203	-0.925403	-0.706837	-2.261576	-0.195854
	B	-1.041042	-0.828536	-0.430470	-0.942637	-1.089536	-0.118422
	C	-2.078936	0.015525	-1.246010	-1.977956	-0.613637	-1.203100
	N	-2.881706	0.608952	-1.838787	-2.763653	-0.256033	-1.972322
	H	-1.582841	-1.040618	0.752056	-1.525804	-0.849970	1.039316
	C	-2.315265	-1.420385	2.210137	-2.258478	-0.446130	2.437514
	H	-1.454640	-1.735087	2.789976	-1.533475	0.233035	2.873561
	H	-2.760199	-0.467900	2.475687	-3.175673	-0.001531	2.066967
	H	-2.995438	-2.199994	1.886565	-2.352390	-1.417421	2.909397
**TS6**	C	-1.986730	0.311786	-0.000976	-1.975420	0.227623	0.005602
	C	-1.534894	-0.974448	-0.024335	-1.433752	-1.018614	0.050526
	N	-0.143580	-0.905016	0.031493	-0.056054	-0.845597	0.076098
	N	-0.858267	1.129146	0.078936	-0.911219	1.121480	0.016016
	C	0.274005	0.383037	0.103337	0.264080	0.464454	0.063438
	H	1.878717	2.089473	0.158797	1.802530	2.246333	-0.026928
	B	1.781886	0.892379	0.123287	1.742285	1.051921	0.022657
	C	2.660815	0.242857	1.246923	2.637467	0.469780	1.179158
	N	3.348210	-0.226213	2.057194	3.320005	0.027826	2.001524
	C	-3.372418	0.860497	-0.040664	-3.392079	0.683418	-0.042666
	H	-3.614529	1.414846	0.871168	-3.654276	1.263301	0.845613
	H	-3.513772	1.537677	-0.888039	-3.577168	1.307106	-0.920453
	H	-4.094582	0.050530	-0.138133	-4.057208	-0.177143	-0.092605
	C	-2.276230	-2.266020	-0.094890	-2.073510	-2.363151	0.068816
	H	-2.041686	-2.820068	-1.008974	-1.826607	-2.935410	-0.828796
	H	-2.040647	-2.912226	0.755559	-1.753462	-2.940829	0.939027
	H	-3.350120	-2.082268	-0.085843	-3.156146	-2.255807	0.112941
	H	2.326116	0.529146	-1.027670	2.260417	0.571145	-1.092869
	C	3.035908	0.181512	-2.482736	2.820626	-0.104296	-2.457473
	H	3.467520	1.145374	-2.728700	3.080969	0.774250	-3.036685
	H	3.732967	-0.599461	-2.198984	3.650525	-0.692415	-2.080578
	H	2.177448	-0.126198	-3.070246	1.951197	-0.659650	-2.793994
	C	0.739672	-2.069375	0.022658	0.911563	-1.934424	0.139937
	H	1.724688	-1.764820	-0.319057	1.835240	-1.615075	-0.337328
	H	0.822203	-2.499026	1.022636	1.114116	-2.203738	1.177396
	H	0.343277	-2.818711	-0.660714	0.511248	-2.795401	-0.391686
	C	-0.927472	2.588299	0.123692	-1.087618	2.567055	-0.028793

	H	-1.273977	2.979552	-0.834503	-1.480307	2.863975	-1.001717
	H	-1.615324	2.899732	0.909932	-1.783657	2.871288	0.752160
	H	0.061780	2.980259	0.334926	-0.126092	3.041418	0.136487
**TS7**	C	2.977098	-1.560189	0.337322	2.950930	-1.564513	0.323241
	H	3.502418	-2.349835	0.845814	3.457228	-2.382101	0.807009
	C	3.261893	-0.900195	-0.811464	3.251639	-0.868768	-0.799317
	H	4.083155	-1.004598	-1.498720	4.071523	-0.960417	-1.490486
	N	2.241570	0.010950	-1.016091	2.252531	0.066757	-0.967347
	N	1.790394	-1.038037	0.815129	1.778172	-1.034571	0.814690
	C	1.334922	-0.060650	-0.008605	1.350852	-0.026740	0.029064
	B	-0.004439	0.798132	0.224258	0.011581	0.811350	0.275613
	C	0.086067	2.254146	-0.368552	0.108765	2.296124	-0.247313
	N	0.117821	3.356437	-0.730345	0.148671	3.405873	-0.568340
	C	-1.381314	0.042737	-0.138561	-1.329762	0.054621	-0.167439
	C	-1.435889	-1.282741	-0.604846	-1.341158	-1.252029	-0.673833
	C	-2.615763	0.699014	0.037564	-2.573928	0.684403	0.000220
	C	-2.648214	-1.921712	-0.877613	-2.531795	-1.902047	-0.998515
	H	-0.515616	-1.835809	-0.769579	-0.402461	-1.779607	-0.826745
	C	-3.830015	0.071699	-0.232477	-3.766367	0.045994	-0.321712
	H	-2.625428	1.725294	0.392602	-2.604447	1.698359	0.390123
	C	-3.853452	-1.247168	-0.692223	-3.750457	-1.254904	-0.823571
	H	-2.648518	-2.945097	-1.239070	-2.505404	-2.912833	-1.390852
	H	-4.760426	0.610671	-0.085031	-4.711018	0.560226	-0.181910
	H	-4.796865	-1.738377	-0.904151	-4.677828	-1.755973	-1.075930
	H	-0.040387	1.065657	1.519109	-0.085995	0.989142	1.574547
	C	-0.213518	1.608520	3.098160	-0.530857	1.354531	3.107350
	H	-0.811235	0.816820	3.535971	-1.095576	0.468164	3.374946
	H	0.820757	1.672073	3.418461	0.427855	1.488651	3.596404
	H	-0.718227	2.549462	2.911374	-1.105264	2.254910	2.921061
	C	2.167746	0.886043	-2.190306	2.171331	0.992154	-2.096365
	H	1.145608	0.920739	-2.559421	1.149557	1.013250	-2.470222
	H	2.496377	1.893778	-1.939552	2.466072	1.993397	-1.787044
	H	2.813735	0.471782	-2.961909	2.837906	0.631309	-2.876196
	C	1.155614	-1.481835	2.058587	1.100847	-1.492111	2.025899
	H	1.577477	-0.946026	2.909188	1.351758	-0.841771	2.862865
	H	0.086863	-1.298801	2.005497	0.025079	-1.479591	1.860629

	H	1.332969	-2.549859	2.175345	1.427243	-2.508570	2.234463
**TS8**	C	0.194626	0.147099	-0.087002	0.222435	0.158324	-0.164922
	C	0.087970	-2.084529	0.180549	0.026280	-2.056270	0.097473
	H	-0.373179	-3.050182	0.287848	-0.476176	-2.999626	0.223286
	N	-0.641677	-0.918507	0.033263	-0.649339	-0.857602	0.009455
	N	1.452716	-0.364901	-0.007079	1.449553	-0.405649	-0.176604
	C	-2.124549	-0.859935	0.027927	-2.118233	-0.718910	0.069023
	H	-2.365790	0.200258	-0.011076	-2.300618	0.347235	0.198367
	C	2.722664	0.399215	-0.092727	2.716536	0.352273	-0.256316
	H	2.418038	1.424537	-0.287755	2.502247	1.199602	-0.906953
	H	0.721260	2.425136	-0.231159	0.824605	2.424609	-0.298694
	B	-0.222352	1.682698	-0.193481	-0.132662	1.711109	-0.209342
	C	1.396768	-1.738442	0.159821	1.345766	-1.771895	-0.008665
	H	2.277378	-2.349536	0.247298	2.201176	-2.423193	0.012837
	C	3.466279	0.347749	1.244109	3.093081	0.854899	1.132796
	H	2.838385	0.722617	2.055249	2.296780	1.476023	1.547310
	H	3.781595	-0.669733	1.490742	3.269531	0.010926	1.804824
	H	4.361017	0.971652	1.187137	4.006661	1.449514	1.076424
	C	3.568832	-0.094495	-1.268700	3.815046	-0.491716	-0.886545
	H	3.005192	-0.048637	-2.202922	3.492755	-0.917544	-1.838275
	H	4.450038	0.542855	-1.369330	4.679988	0.146591	-1.070537
	H	3.914995	-1.120686	-1.120372	4.136299	-1.298485	-0.223931
	C	-2.681918	-1.542774	-1.223334	-2.735543	-1.187865	-1.243198
	H	-2.452132	-2.611766	-1.230644	-2.539073	-2.251484	-1.401624
	H	-3.768207	-1.431760	-1.245310	-3.816200	-1.038869	-1.213167
	H	-2.273809	-1.091468	-2.129549	-2.330323	-0.621198	-2.082704
	C	-2.689454	-1.441074	1.326482	-2.669289	-1.462102	1.279797
	H	-3.772273	-1.299706	1.341958	-3.736163	-1.252456	1.369665
	H	-2.492053	-2.512757	1.411549	-2.549632	-2.543102	1.175947
	H	-2.265906	-0.938137	2.198179	-2.173587	-1.137394	2.196204
	C	-1.210040	2.009906	-1.364578	-1.222396	2.057086	-1.289357
	N	-1.952535	2.296151	-2.210571	-2.045123	2.325492	-2.056575
	H	-0.859330	1.985927	0.925050	-0.697227	1.920928	0.964397
	C	-1.573831	2.477752	2.332615	-1.335085	1.902711	2.456126
	H	-0.997980	1.921937	3.064793	-2.398182	1.821200	2.255011
	H	-1.389882	3.545291	2.284686	-0.854139	1.020094	2.865107

	H	-2.599205	2.158628	2.180766	-0.992640	2.856191	2.842004
**TS9**	C	-2.137170	1.448579	0.093510	-2.005826	1.558239	0.000944
	H	-2.606347	2.414838	0.156819	-2.399052	2.559936	0.016167
	C	-2.668635	0.201345	0.082808	-2.627121	0.354895	0.027459
	H	-3.691312	-0.128676	0.135355	-3.671472	0.098403	0.073356
	N	-1.614052	-0.689550	-0.014357	-1.639782	-0.608120	-0.018617
	N	-0.765607	1.299674	0.009394	-0.652577	1.303609	-0.051491
	C	-0.440402	-0.014021	-0.063565	-0.428882	-0.023812	-0.069993
	B	1.048758	-0.606191	-0.083580	1.015793	-0.712783	-0.036550
	H	1.643973	-0.142400	1.010429	1.599853	-0.210409	1.047036
	C	2.439233	0.088751	2.425501	2.259276	0.208445	2.440319
	H	1.635974	0.415131	3.077784	1.466913	0.794005	2.895751
	H	2.826392	-0.908499	2.602834	2.448224	-0.761615	2.886584
	H	3.166351	0.842218	2.142003	3.134602	0.760987	2.115180
	C	-1.786544	-2.147081	-0.067614	-1.895165	-2.049794	-0.007389
	H	-1.348663	-2.612098	0.813206	-1.557340	-2.480205	0.933033
	H	-2.854012	-2.354563	-0.098646	-2.966559	-2.197206	-0.119633
	H	-1.311218	-2.547523	-0.959956	-1.368089	-2.521626	-0.832139
	C	0.189769	2.408652	-0.025091	0.392858	2.321448	-0.116009
	H	0.516154	2.598589	-1.047848	0.719409	2.459520	-1.146382
	H	-0.297035	3.296488	0.372958	-0.010359	3.252903	0.274902
	H	1.052591	2.163861	0.591540	1.237049	2.004059	0.494417
	C	1.908522	-0.065198	-1.298873	1.914427	-0.230973	-1.256909
	N	2.578666	0.351254	-2.148522	2.598082	0.165551	-2.098814
	F	1.066853	-2.029134	-0.020470	0.943378	-2.116979	0.068490
**TS10**	C	-2.580798	-0.873627	-0.116833	-2.544741	-0.874236	-0.076274
	H	-3.309202	-1.662724	-0.182539	-3.266065	-1.670868	-0.134455
	C	-2.729749	0.473362	-0.102467	-2.701706	0.470531	-0.058029
	H	-3.613219	1.084793	-0.156770	-3.587892	1.079870	-0.098159
	N	-1.464429	1.021128	0.001514	-1.440813	1.021503	0.031142
	N	-1.226539	-1.127652	-0.030345	-1.191415	-1.115786	-0.006757
	C	-0.533987	0.036193	0.046355	-0.512851	0.046602	0.062879
	B	1.063878	0.119128	0.116582	1.079449	0.126658	0.100314
	C	1.710903	1.539793	-0.028702	1.713615	1.556501	-0.014539
	N	2.254376	2.550313	-0.186041	2.223240	2.581784	-0.154534
	H	1.506448	-0.525336	-0.942768	1.473292	-0.491710	-0.987308

	C	2.158046	-1.208178	-2.322958	1.938493	-1.176751	-2.387781
	H	1.265626	-1.377732	-2.915029	0.980375	-1.344229	-2.867562
	H	2.798427	-0.388731	-2.627512	2.545794	-0.370857	-2.782340
	H	2.646843	-2.081267	-1.905828	2.466996	-2.052564	-2.028685
	C	-1.221998	2.466959	0.072131	-1.187315	2.461566	0.096351
	H	-0.628197	2.703817	0.952112	-0.577777	2.688783	0.967998
	H	-0.706035	2.812015	-0.821793	-0.678938	2.795736	-0.805694
	H	-2.187047	2.962858	0.146849	-2.148144	2.962534	0.183877
	C	-0.649499	-2.473615	0.020156	-0.588422	-2.446392	0.037361
	H	-0.458629	-2.764619	1.053239	-0.369001	-2.722383	1.068231
	H	-1.356090	-3.166888	-0.431199	-1.291526	-3.153523	-0.396307
	H	0.281362	-2.493259	-0.540989	0.331913	-2.443425	-0.543211
	C	1.652325	-0.647445	1.357583	1.679651	-0.694980	1.300802
	N	2.095000	-1.240820	2.248832	2.115184	-1.340028	2.152639
**TS11**	C	0.537982	0.000020	0.458549	0.542398	0.150189	0.465670
	C	2.417213	-0.677461	-0.601309	1.793503	-1.445268	-0.512030
	C	2.416975	0.678074	-0.601364	2.462726	-0.274240	-0.627609
	H	3.128834	-1.380441	-0.998098	2.044612	-2.438638	-0.841737
	H	3.128346	1.381271	-0.998216	3.412698	-0.045810	-1.079071
	N	1.263093	1.081056	0.050448	1.681954	0.692490	-0.023427
	N	1.263470	-1.080792	0.050528	0.618569	-1.165812	0.157204
	C	0.870473	2.471094	0.259861	2.017291	2.107458	0.060981
	H	1.661380	3.114521	-0.121357	3.000003	2.254109	-0.382165
	H	-0.058169	2.687893	-0.269134	1.278743	2.697372	-0.480663
	H	0.725376	2.663600	1.322502	2.032431	2.422176	1.103215
	C	0.871319	-2.470943	0.260067	-0.421001	-2.130391	0.484444
	H	1.662198	-3.114145	-0.121588	-0.200246	-3.064751	-0.027207
	H	0.726800	-2.663558	1.322771	-0.454340	-2.297428	1.561011
	H	-0.057530	-2.687938	-0.268477	-1.384826	-1.745211	0.152469
	H	-1.087878	-1.015971	1.770764	-1.205574	0.196230	1.985068
	H	-1.088497	1.015566	1.770395	-0.439235	2.011838	1.456695
	B	-0.872118	-0.000247	1.148594	-0.686278	0.877031	1.130445
	H	-1.760053	-0.000737	0.150662	-1.566776	0.899133	0.130907
	C	-3.014596	-0.001371	-0.930856	-2.747243	0.701175	-0.976599
	C	-4.259130	0.000647	-0.090055	-3.897413	0.231991	-0.133474
	H	-2.780604	-0.913984	-1.474406	-2.274908	-0.027931	-1.631503

	H	-2.779325	0.909398	-1.476942	-2.809800	1.700873	-1.397943
	H	-4.305101	0.884892	0.553774	-4.184644	0.995610	0.594161
	H	-5.176226	0.000457	-0.697957	-4.789256	-0.005228	-0.727135
	H	-4.306318	-0.881798	0.556152	-3.632714	-0.669722	0.426938
**TS12**	C	1.177047	-0.417463	0.182395	1.089045	-0.395604	0.244653
	C	3.088361	-1.621191	0.132976	3.004968	-1.521359	-0.097003
	C	2.640866	-1.518258	-1.142043	2.472122	-1.247920	-1.311111
	H	3.958231	-2.110826	0.535244	3.906609	-2.040986	0.178059
	H	3.045561	-1.900587	-2.063228	2.818465	-1.479209	-2.303862
	N	1.472583	-0.777676	-1.098336	1.298697	-0.558714	-1.083253
	N	2.181614	-0.948071	0.934204	2.140944	-0.998869	0.843719
	C	0.692639	-0.415784	-2.279382	0.449521	-0.011916	-2.134286
	H	0.106606	-1.267801	-2.627493	-0.241052	-0.770802	-2.503032
	H	0.022893	0.398802	-2.019189	-0.116890	0.820487	-1.721719
	H	1.370150	-0.093543	-3.070367	1.083262	0.338668	-2.948163
	C	2.315006	-0.815741	2.384320	2.359955	-1.049013	2.284456
	H	2.635235	0.192419	2.649693	3.172357	-1.745364	2.481078
	H	1.361056	-1.024407	2.863619	2.623970	-0.060033	2.658524
	H	3.059072	-1.533058	2.726446	1.453685	-1.391075	2.779242
	H	0.163992	0.831568	1.836823	0.108000	0.554066	2.117399
	B	-0.007169	0.518852	0.678278	-0.066208	0.456091	0.924989
	H	0.187261	1.669866	0.021295	0.182643	1.663132	0.430372
	C	0.434011	3.177575	-0.614323	0.804439	3.054342	-0.169312
	H	-0.430248	3.689316	-0.198411	0.537020	3.729530	0.638843
	H	0.349903	2.967623	-1.678238	0.241222	3.208858	-1.086021
	C	-1.511070	0.035053	0.391314	-1.561871	0.060410	0.509044
	C	-2.587887	0.905855	0.666060	-2.623439	0.936285	0.798349
	C	-1.862343	-1.239647	-0.095222	-1.906030	-1.138397	-0.135548
	C	-3.916835	0.537182	0.467030	-3.941734	0.639599	0.467141
	H	-2.373458	1.900444	1.047768	-2.404016	1.877775	1.295532
	C	-3.190784	-1.622439	-0.299809	-3.222485	-1.447500	-0.477454
	H	-1.081498	-1.964202	-0.310323	-1.126272	-1.859458	-0.371131
	C	-4.229196	-0.734821	-0.020809	-4.249788	-0.557901	-0.177833
	H	-4.712740	1.240574	0.692617	-4.732516	1.342629	0.707509
	H	-3.414104	-2.617631	-0.672525	-3.446017	-2.385756	-0.974561
	H	-5.262084	-1.027382	-0.176454	-5.275142	-0.792783	-0.440515

	C	1.785918	3.576418	-0.095321	2.250752	2.668104	-0.289945
	H	2.567079	2.890338	-0.439746	2.405162	1.944087	-1.095266
	H	2.081768	4.584235	-0.422568	2.902108	3.526504	-0.495632
	H	1.807392	3.579771	0.999443	2.608216	2.207576	0.638245
**TS13**	C	-2.364749	1.198713	-0.475011	-1.893623	1.703967	-0.288529
	C	-2.622084	-0.107941	-0.721962	-2.567510	0.568354	-0.585152
	N	-1.553664	-0.834293	-0.218798	-1.759192	-0.488990	-0.210402
	N	-1.145536	1.254046	0.177930	-0.688458	1.316558	0.261227
	C	-0.633984	0.000391	0.338612	-0.603222	-0.030518	0.316238
	H	1.122107	0.478847	1.797868	1.159985	-0.363741	1.808083
	B	0.809983	-0.300824	0.924448	0.695918	-0.817711	0.784766
	H	1.653182	-0.072742	-0.097062	1.583025	-0.514344	-0.184412
	C	2.874030	-0.069191	-1.174951	2.785035	-0.195447	-1.185089
	H	2.884673	-1.121386	-1.450114	2.885034	-1.139022	-1.715453
	H	2.440798	0.577676	-1.935370	2.352844	0.602512	-1.786057
	C	-1.448726	-2.289745	-0.336474	-2.118581	-1.897512	-0.357210
	H	-0.577913	-2.624850	0.216559	-2.244335	-2.356205	0.621805
	H	-2.346517	-2.752745	0.075154	-3.053641	-1.949711	-0.910576
	H	-1.348871	-2.567956	-1.387343	-1.336395	-2.421529	-0.900910
	C	-0.492264	2.484964	0.615219	0.365307	2.209167	0.723447
	H	0.451916	2.622606	0.086953	1.316694	1.890704	0.297091
	H	-1.152164	3.321930	0.395457	0.133789	3.219387	0.393601
	H	-0.297447	2.446003	1.686800	0.430942	2.180981	1.811252
	H	-2.936924	2.083117	-0.695753	-2.157520	2.739048	-0.420341
	H	-3.461847	-0.582528	-1.199488	-3.537532	0.417208	-1.026710
	F	0.995556	-1.675391	1.327016	0.488493	-2.230491	0.820835
	C	4.067874	0.456268	-0.427951	3.893260	0.179758	-0.241265
	H	4.325505	-0.188755	0.418144	4.141153	-0.654322	0.420624
	H	4.963931	0.523340	-1.062612	4.814796	0.468038	-0.761984
	H	3.883472	1.461061	-0.033402	3.604682	1.024375	0.392229
**TS14**	C	-2.747065	-1.390181	-1.228046	-2.592660	-1.638236	-0.908358
	H	-3.255469	-1.614422	-2.149857	-3.074375	-2.090834	-1.758157
	C	-3.007551	-1.771208	0.044997	-2.886422	-1.683341	0.412232
	H	-3.785816	-2.395577	0.448765	-3.676094	-2.185268	0.944871
	N	-2.042768	-1.187484	0.848675	-1.924823	-0.935658	1.061429
	N	-1.629693	-0.575376	-1.182014	-1.461307	-0.858689	-1.035870

	C	-1.184324	-0.447617	0.097646	-1.053269	-0.416590	0.172948
	B	0.049029	0.474547	0.568782	0.124054	0.647554	0.434074
	C	1.517929	-0.049818	0.213251	1.614098	0.130911	0.200986
	C	1.801264	-1.133208	-0.639550	1.950286	-1.065661	-0.450608
	C	2.629972	0.618610	0.767043	2.681912	0.932166	0.641504
	C	3.109696	-1.522752	-0.933571	3.275779	-1.439172	-0.665878
	H	0.986714	-1.703344	-1.076777	1.163178	-1.735642	-0.788460
	C	3.939733	0.234517	0.485699	4.008171	0.566453	0.437415
	H	2.459779	1.456200	1.436502	2.461411	1.862006	1.157467
	C	4.188457	-0.839654	-0.372303	4.312947	-0.622786	-0.223791
	H	3.286493	-2.367150	-1.592512	3.499112	-2.372940	-1.170790
	H	4.769359	0.772750	0.933552	4.807827	1.208314	0.791660
	H	5.206305	-1.141424	-0.594684	5.345229	-0.910531	-0.386987
	H	-0.126358	1.655345	-0.066052	-0.176115	1.581884	-0.478796
	C	-0.307900	3.225720	-0.421337	-1.025064	2.769404	-1.143268
	H	0.082178	3.261190	-1.436881	-0.804233	2.671602	-2.203479
	H	0.367555	3.653673	0.315716	-0.551125	3.624443	-0.668146
	C	-1.935926	-1.463482	2.284609	-1.878418	-0.735431	2.508525
	H	-2.930432	-1.415220	2.727942	-2.319931	0.225555	2.768762
	H	-1.519698	-2.460795	2.438004	-2.436041	-1.542493	2.978932
	H	-1.291892	-0.720609	2.741466	-0.844390	-0.757327	2.840557
	C	-1.041373	0.057004	-2.362402	-0.867104	-0.493577	-2.318075
	H	-0.471068	-0.672161	-2.939435	-0.611949	-1.397722	-2.870204
	H	-1.839325	0.465951	-2.982078	-1.579436	0.101842	-2.891118
	H	-0.383338	0.858751	-2.041450	0.032124	0.088673	-2.137091
	F	-0.079260	0.823484	1.971704	-0.059239	1.285521	1.704131
	C	-1.774022	3.509075	-0.242413	-2.416495	2.427162	-0.686814
	H	-2.104597	3.272698	0.774248	-2.427217	2.212143	0.387740
	H	-2.021819	4.566167	-0.419226	-3.132466	3.239194	-0.861600
	H	-2.386497	2.921870	-0.934845	-2.802281	1.542269	-1.202516
**TS15**	C	-0.709608	-0.043468	0.221142	-0.752135	-0.169217	0.155711
	C	-2.556818	0.537545	-0.929558	-2.122893	1.490603	-0.469580
	C	-2.797138	-0.665164	-0.352246	-2.845848	0.345818	-0.454189
	H	-3.177976	1.155228	-1.554424	-2.402562	2.501766	-0.710577
	H	-3.667711	-1.296844	-0.379218	-3.881619	0.161023	-0.681199
	N	-1.656503	-1.010136	0.351625	-1.987700	-0.663223	-0.067149

	N	-1.270769	0.904359	-0.575509	-0.837838	1.153425	-0.099461
	C	-1.503981	-2.250994	1.112144	-2.360522	-2.067627	0.071557
	H	-0.761722	-2.896686	0.642925	-1.830106	-2.668677	-0.665407
	H	-1.193341	-2.027761	2.131297	-2.111901	-2.415533	1.072263
	H	-2.465368	-2.760393	1.129241	-3.432446	-2.151917	-0.091044
	C	-0.616370	2.138591	-1.008477	0.278804	2.086504	0.001457
	H	-0.618090	2.877104	-0.206629	0.464721	2.344878	1.043455
	H	0.410945	1.924489	-1.297495	1.169309	1.619609	-0.417792
	H	-1.156580	2.532064	-1.867409	0.031670	2.980497	-0.566619
	H	0.922633	-1.004418	1.562753	0.343872	-2.141820	0.740418
	B	0.765787	-0.097529	0.789016	0.554354	-0.982582	0.522130
	C	1.294931	1.245087	1.393405	1.389165	-0.302442	1.666784
	N	1.733872	2.222001	1.843238	2.024426	0.195707	2.495392
	H	1.555758	-0.374567	-0.246832	1.315219	-0.892240	-0.563934
	C	2.629451	-0.795138	-1.412850	2.355547	-0.678672	-1.766302
	H	2.023769	-1.526775	-1.942521	2.353552	-1.674344	-2.201259
	H	2.716986	0.161435	-1.922340	1.832065	0.067376	-2.360175
	C	3.834763	-1.297704	-0.673152	3.593502	-0.243950	-1.037260
	H	4.632909	-1.617867	-1.358949	4.430892	-0.054676	-1.720274
	H	4.261348	-0.523535	-0.028182	3.420997	0.676918	-0.473137
	H	3.589930	-2.159924	-0.045227	3.918635	-1.007422	-0.325918
**TS16**	C	-2.122571	-0.398535	-0.131284	-1.996847	-0.217399	-0.222997
	C	-1.783113	0.711865	0.582583	-1.476685	1.033585	-0.107255
	N	-0.425582	0.931424	0.350933	-0.101690	0.901099	-0.254522
	C	0.341767	2.027689	0.937012	0.859958	1.991386	-0.149874
	H	1.393299	1.755692	0.954581	1.742399	1.634918	0.380435
	H	0.217043	2.942653	0.354863	1.152620	2.343096	-1.139361
	H	0.003505	2.200710	1.957445	0.408985	2.807479	0.410547
	N	-0.965379	-0.819724	-0.788485	-0.924702	-1.072780	-0.448461
	C	-0.922348	-1.988964	-1.663353	-1.062914	-2.516118	-0.584800
	H	0.045509	-2.025950	-2.152069	-0.163339	-2.913059	-1.044437
	H	-1.068836	-2.901399	-1.082389	-1.205223	-2.974625	0.395258
	H	-1.705341	-1.913657	-2.418467	-1.921427	-2.737044	-1.217066
	C	0.078744	-0.002118	-0.496335	0.237298	-0.389162	-0.458456
	H	1.774272	-1.067114	-1.709548	1.791103	-2.137723	-0.696867
	B	1.589152	-0.142763	-0.964154	1.716712	-0.955419	-0.522861

	C	2.206529	1.166975	-1.557976	2.637013	-0.154451	-1.512127
	N	2.705310	2.121761	-1.994002	3.338551	0.438900	-2.215153
	H	2.314595	-0.429017	0.123659	2.180638	-0.699378	0.700323
	C	3.227700	-0.924397	1.365330	2.372790	-0.439937	2.268360
	H	4.168979	-1.063250	0.839286	2.903340	-1.351777	2.528567
	H	3.202233	-0.036409	1.992939	2.990916	0.453990	2.285333
	C	-2.602670	1.590227	1.465600	-2.137340	2.346920	0.131435
	H	-2.250968	1.566513	2.501614	-1.834366	2.777501	1.089206
	H	-2.580168	2.631216	1.130491	-1.891558	3.062143	-0.656706
	H	-3.641489	1.261560	1.461247	-3.218373	2.218631	0.146743
	C	-3.425561	-1.110259	-0.266793	-3.401885	-0.706900	-0.156285
	H	-3.788004	-1.100017	-1.299164	-3.727062	-1.119321	-1.114693
	H	-3.348804	-2.155368	0.047119	-3.512879	-1.485309	0.602177
	H	-4.180977	-0.630989	0.355067	-4.069103	0.113806	0.101653
	C	2.539711	-2.154697	1.883375	0.951360	-0.317941	2.738858
	H	2.465235	-2.927030	1.111242	0.334509	-1.131370	2.339892
	H	3.078047	-2.602949	2.731139	0.871302	-0.361057	3.831700
	H	1.526536	-1.932333	2.233651	0.502358	0.625771	2.417396
**TS17**	C	-2.607332	-1.677283	-1.210936	-2.486328	-2.031106	-0.425827
	H	-3.020606	-2.080489	-2.119013	-2.910878	-2.757700	-1.097377
	C	-2.983350	-1.831588	0.081138	-2.815054	-1.688746	0.841478
	H	-3.789726	-2.394783	0.517613	-3.583543	-2.058445	1.498122
	N	-2.098385	-1.097260	0.850609	-1.927150	-0.708897	1.233875
	N	-1.504753	-0.844203	-1.210480	-1.408405	-1.248938	-0.780244
	C	-1.183103	-0.477613	0.058886	-1.066837	-0.425757	0.234256
	B	0.018502	0.509066	0.445865	0.092328	0.671588	0.153161
	C	-0.139344	1.200851	1.849032	-0.112562	1.873758	1.150616
	N	-0.214690	1.793710	2.844554	-0.242365	2.809775	1.816950
	C	1.503847	-0.065404	0.196430	1.589821	0.109376	0.118270
	C	1.766896	-1.403086	-0.148493	1.897664	-1.258691	0.096418
	C	2.621353	0.783825	0.318672	2.671748	1.004291	0.070261
	C	3.065802	-1.869902	-0.364473	3.213825	-1.713835	0.027527
	H	0.944662	-2.106709	-0.242966	1.096143	-1.991709	0.144440
	C	3.921340	0.328350	0.108158	3.987610	0.560948	0.003277
	H	2.469299	1.825144	0.586896	2.474557	2.072756	0.088270
	C	4.151680	-1.005259	-0.237758	4.266084	-0.805387	-0.019918

	H	3.228241	-2.910671	-0.626244	3.416226	-2.779371	0.016848
	H	4.756974	1.012972	0.213048	4.799363	1.279437	-0.031666
	H	5.162014	-1.363449	-0.402746	5.290621	-1.155422	-0.071163
	H	-0.126838	1.570192	-0.358274	-0.167754	1.267370	-0.996334
	C	-0.254445	3.007732	-1.152148	-0.922627	2.118538	-2.160479
	H	0.241383	2.778074	-2.092447	-0.626696	1.609024	-3.073236
	H	0.349857	3.600174	-0.470245	-0.431743	3.073669	-1.995448
	C	-2.161662	-1.050423	2.314248	-1.925696	-0.098001	2.562112
	H	-1.168451	-1.193928	2.733076	-0.904559	-0.037857	2.931914
	H	-2.562161	-0.096028	2.652785	-2.357139	0.900784	2.520727
	H	-2.811478	-1.856882	2.647838	-2.515883	-0.730107	3.221712
	C	-0.783874	-0.465782	-2.426189	-0.775314	-1.298539	-2.095164
	H	-0.164448	0.400208	-2.217400	0.138823	-0.712288	-2.073290
	H	-0.152947	-1.290611	-2.760090	-0.538668	-2.334659	-2.334105
	H	-1.506307	-0.220394	-3.204150	-1.457120	-0.892943	-2.843782
	C	-1.726034	3.301575	-1.195718	-2.347633	1.993143	-1.706689
	H	-2.270372	2.565672	-1.795753	-2.729433	0.977553	-1.846802
	H	-2.163419	3.306894	-0.192326	-2.439094	2.239890	-0.643198
	H	-1.934033	4.287606	-1.636806	-3.017637	2.668536	-2.252344
**TS18**	C	-0.332585	-0.107763	0.183576	-0.340798	-0.173756	0.271892
	C	-0.805266	-2.086044	-0.783160	-0.552654	-2.109917	-0.833584
	H	-0.614331	-3.064820	-1.185726	-0.232067	-3.021012	-1.308297
	N	0.192107	-1.284916	-0.255571	0.330581	-1.211968	-0.271311
	N	-1.666325	-0.182230	-0.082969	-1.649295	-0.417346	0.037300
	C	1.624658	-1.666912	-0.202807	1.798112	-1.369275	-0.270697
	H	2.140384	-0.781943	0.164246	2.188250	-0.415097	0.080862
	C	-2.685480	0.852137	0.222518	-2.743079	0.515908	0.380216
	H	-2.139742	1.636293	0.741715	-2.444693	0.972632	1.323697
	H	-0.240217	2.068004	1.043742	-0.499610	1.944851	1.273816
	B	0.465603	1.132996	0.777419	0.301033	1.120874	0.935349
	C	-1.965037	-1.395757	-0.680136	-1.795600	-1.606217	-0.649167
	H	-2.962247	-1.666527	-0.978066	-2.754390	-1.997004	-0.939491
	C	-3.269229	1.425443	-1.071051	-2.848748	1.588337	-0.698582
	H	-2.481901	1.832647	-1.709018	-1.899983	2.116517	-0.812538
	H	-3.816497	0.665913	-1.635980	-3.117852	1.133372	-1.655478
	H	-3.966260	2.231070	-0.829929	-3.621331	2.310490	-0.428358

	C	-3.755216	0.290459	1.162543	-4.052965	-0.232139	0.581978
	H	-3.303905	-0.117367	2.069520	-3.938135	-1.052222	1.292649
	H	-4.438490	1.092420	1.450619	-4.792505	0.464959	0.977679
	H	-4.345882	-0.494864	0.683493	-4.441874	-0.625442	-0.360048
	C	1.836934	-2.812619	0.789710	2.202336	-2.471909	0.699910
	H	1.322659	-3.721463	0.465405	1.797514	-3.435563	0.379548
	H	2.903095	-3.037379	0.864984	3.290322	-2.551097	0.731044
	H	1.473860	-2.541481	1.782673	1.840848	-2.248639	1.704810
	C	2.148615	-1.988898	-1.604691	2.300382	-1.611076	-1.688998
	H	3.225715	-2.162421	-1.554023	3.391364	-1.619835	-1.684152
	H	1.685972	-2.890165	-2.015350	1.959783	-2.573890	-2.077213
	H	1.966996	-1.159762	-2.291566	1.961961	-0.819331	-2.359929
	C	1.398615	0.799921	1.987576	1.318855	0.779154	2.082367
	N	2.115796	0.597546	2.879396	2.099894	0.548284	2.904233
	H	1.266300	1.573204	-0.199835	1.032380	1.631567	-0.047274
	C	2.197441	2.254031	-1.333300	1.863973	2.259632	-1.252298
	H	1.531132	2.163886	-2.188414	1.273122	1.920879	-2.100322
	H	3.024133	1.547351	-1.343039	2.819005	1.751037	-1.141052
	C	2.483589	3.638376	-0.826153	1.817355	3.721993	-0.914853
	H	3.005545	3.612877	0.135358	2.270683	3.912224	0.061607
	H	3.119328	4.207660	-1.519933	2.354380	4.335203	-1.648848
	H	1.563058	4.214837	-0.691715	0.786401	4.083828	-0.880113
**TS19**	C	-2.420169	1.441142	0.491415	-2.209506	1.702674	0.187384
	H	-2.860620	2.403299	0.686520	-2.511465	2.735289	0.221266
	C	-2.947558	0.194089	0.550758	-2.914046	0.560874	0.370193
	H	-3.936367	-0.140476	0.811619	-3.953390	0.399644	0.598710
	N	-1.944492	-0.690158	0.191893	-2.030705	-0.487367	0.208352
	N	-1.099513	1.300177	0.107171	-0.908666	1.327826	-0.073877
	C	-0.803284	-0.010403	-0.084781	-0.800152	-0.015437	-0.067887
	B	0.642466	-0.594477	-0.435160	0.566044	-0.824363	-0.228525
	H	1.444648	-0.205624	0.570782	1.354561	-0.357725	0.752663
	C	2.477132	-0.115396	1.798632	2.436818	0.013264	1.826866
	H	2.076416	0.753600	2.316712	1.980820	0.876281	2.307918
	H	2.248578	-1.059538	2.287473	2.439277	-0.882736	2.442054
	C	-2.130825	-2.145208	0.140212	-2.400438	-1.899520	0.316861
	H	-1.639087	-2.621557	0.987720	-2.094501	-2.429242	-0.581615

	H	-3.199412	-2.346623	0.179200	-1.918227	-2.346073	1.183717
	H	-1.717569	-2.540035	-0.783384	-3.480985	-1.952092	0.426441
	C	-0.171313	2.414303	-0.088481	0.192198	2.246499	-0.347213
	H	-0.107766	2.679384	-1.144293	0.358126	2.327463	-1.421167
	H	-0.531635	3.268513	0.481468	-0.061967	3.220189	0.065423
	H	0.815460	2.131488	0.273085	1.095430	1.870876	0.132052
	C	1.257819	0.023602	-1.753023	1.322845	-0.437175	-1.570059
	N	1.751846	0.491349	-2.692868	1.913523	-0.117990	-2.510362
	F	0.669491	-2.020327	-0.461911	0.400605	-2.217171	-0.074216
	C	3.837680	0.022350	1.176197	3.645265	0.259726	0.968385
	H	4.070294	-0.828471	0.528675	3.912036	-0.636373	0.402219
	H	3.915517	0.932178	0.573342	3.464488	1.060768	0.245534
	H	4.632014	0.072743	1.935140	4.523180	0.547388	1.559375
**TS20**	C	-2.706207	-1.219089	-0.456483	-2.362041	-1.710717	-0.243263
	H	-3.292829	-2.107327	-0.612960	-2.727769	-2.720596	-0.310822
	C	-3.033647	0.091424	-0.559982	-2.996472	-0.522319	-0.375677
	H	-3.960786	0.567375	-0.826988	-4.026080	-0.289361	-0.585169
	N	-1.900879	0.819804	-0.245404	-2.049700	0.462652	-0.187404
	N	-1.376018	-1.272152	-0.089052	-1.039260	-1.427170	0.014141
	C	-0.874556	-0.017865	0.048460	-0.846382	-0.093206	0.052800
	B	0.646966	0.301873	0.418238	0.580157	0.577400	0.268535
	C	1.089454	1.804591	0.401836	0.662887	2.135600	0.123742
	N	1.495872	2.888704	0.357360	0.787434	3.272390	-0.030274
	H	1.377438	-0.238399	-0.555522	1.325129	0.124047	-0.723247
	C	2.389550	-0.725767	-1.753792	2.420056	-0.336918	-1.804984
	H	2.104821	-1.772057	-1.834431	1.921746	-1.176514	-2.283247
	H	1.994420	-0.093913	-2.545271	2.447665	0.569572	-2.402899
	C	-1.875306	2.286687	-0.229985	-2.348518	1.894582	-0.228870
	H	-1.530417	2.645674	0.737069	-2.003436	2.368277	0.687445
	H	-1.223463	2.666228	-1.014546	-1.863872	2.356809	-1.086479
	H	-2.890048	2.638202	-0.402503	-3.426894	2.004996	-0.312621
	C	-0.647890	-2.520615	0.148303	-0.006770	-2.433635	0.249458
	H	-0.639656	-2.760017	1.211717	0.153440	-2.564138	1.319185
	H	-1.144474	-3.316671	-0.402846	-0.335954	-3.369917	-0.195241
	H	0.374024	-2.420773	-0.208189	0.921006	-2.112588	-0.220679
	C	1.114567	-0.403844	1.741604	1.283951	0.067737	1.579619

	N	1.477901	-0.945705	2.699722	1.812953	-0.344033	2.519394
	C	3.762938	-0.418379	-1.236594	3.594585	-0.623068	-0.918202
	H	3.988766	-0.983918	-0.328038	3.383334	-1.439794	-0.222708
	H	4.536021	-0.676734	-1.975007	4.480552	-0.909313	-1.498077
	H	3.879800	0.645538	-1.012261	3.862581	0.256321	-0.326855
**TS21**	C	-0.730658	-0.154391	0.591330	-0.486152	0.001409	0.780953
	C	-2.301395	0.321141	-0.953946	-1.790183	-0.679243	-0.912027
	C	-1.701748	1.488290	-0.608122	-1.790457	0.675952	-0.914279
	H	-3.099068	0.118444	-1.647236	-2.282223	-1.387407	-1.555926
	H	-1.874180	2.495074	-0.946870	-2.282782	1.381762	-1.560541
	N	-0.745491	1.182584	0.341747	-0.986389	1.073492	0.131301
	N	-1.697759	-0.676153	-0.210832	-0.986003	-1.073005	0.134888
	C	0.168575	2.157926	0.934793	-0.647794	2.454493	0.450980
	H	-0.259396	3.151466	0.814945	-1.210653	3.107480	-0.212053
	H	0.295156	1.939726	1.992853	-0.904748	2.669153	1.486517
	H	1.139437	2.115718	0.438300	0.420497	2.612432	0.301997
	C	-2.041376	-2.094926	-0.300304	-0.646517	-2.452800	0.458822
	H	-1.227932	-2.653665	-0.763971	0.421486	-2.611149	0.308195
	H	-2.229543	-2.495478	0.693983	-0.901296	-2.663716	1.495649
	H	-2.938690	-2.192562	-0.908044	-1.210674	-3.108231	-0.200686
	H	0.629587	-0.282756	2.484188	0.531329	1.025523	2.613037
	H	-0.136399	-2.047892	1.820196	0.532279	-1.016167	2.616191
	B	0.249416	-0.939232	1.545352	0.554391	0.003692	1.975440
	H	1.353915	-1.123504	0.820396	1.699209	0.003347	1.322881
	C	3.081619	-0.227168	-0.638620	2.206336	-0.002458	-1.005644
	N	3.275181	0.785019	-1.184441	1.553062	-0.007589	-1.964704
	C	2.768920	-1.405005	0.052008	2.914491	0.003644	0.208204
	H	2.450130	-2.252407	-0.542995	3.399315	-0.918725	0.500429
	H	3.362755	-1.631363	0.929347	3.393279	0.930964	0.494664
**TS22**	C	1.026270	-0.690752	0.213414	0.986499	-0.577351	0.264037
	C	2.736723	-2.127293	-0.074861	2.807773	-1.747228	-0.320110
	C	2.546577	-1.478939	-1.250531	2.355506	-1.113802	-1.428834
	H	3.444662	-2.888866	0.202146	3.649501	-2.401589	-0.172813
	H	3.060449	-1.561523	-2.192527	2.728710	-1.100085	-2.438415
	N	1.493004	-0.604717	-1.058750	1.238549	-0.402997	-1.050437
	N	1.796865	-1.635110	0.811998	1.953621	-1.407111	0.707131

	C	1.021686	0.330384	-2.080916	0.474146	0.450190	-1.953293
	H	0.845623	-0.210016	-3.010815	-0.163035	-0.159499	-2.594407
	H	0.093044	0.782940	-1.747431	-0.147575	1.118046	-1.361848
	H	1.770160	1.106693	-2.243966	1.171116	1.031793	-2.554516
	C	1.668988	-2.080814	2.201484	2.093167	-1.868232	2.085760
	H	1.994756	-1.295569	2.883073	2.377183	-1.037585	2.729724
	H	0.633994	-2.338182	2.416559	1.150693	-2.289037	2.428935
	H	2.296034	-2.960107	2.335122	2.867245	-2.631918	2.110112
	H	-0.037832	0.113760	2.105033	-0.051547	-0.116794	2.295753
	B	-0.132041	0.166468	0.901528	-0.167382	0.122692	1.118768
	H	0.201466	1.439868	0.658217	0.146156	1.389078	1.004318
	C	1.828499	3.209203	0.156949	1.934950	2.676399	0.023824
	N	2.943323	3.271857	-0.180688	2.910032	2.395711	-0.539725
	C	0.496189	3.047364	0.556766	0.732211	2.924832	0.704897
	H	0.263440	3.289945	1.586616	0.803522	3.245929	1.735647
	H	-0.267405	3.287507	-0.173806	-0.100922	3.296289	0.121332
	C	-1.629450	-0.040376	0.373308	-1.648348	-0.068834	0.535599
	C	-2.682777	0.670652	0.987166	-2.679031	0.810230	0.905487
	C	-1.994072	-0.907888	-0.674177	-1.995000	-1.107961	-0.341245
	C	-4.007956	0.532820	0.580931	-3.979953	0.663049	0.433169
	H	-2.453792	1.345454	1.807427	-2.452237	1.632484	1.579335
	C	-3.320170	-1.058638	-1.087264	-3.294440	-1.268718	-0.819915
	H	-1.230210	-1.494330	-1.176662	-1.232090	-1.815102	-0.659238
	C	-4.335833	-0.335736	-0.463631	-4.294694	-0.381127	-0.435240
	H	-4.788700	1.098240	1.079988	-4.750240	1.362735	0.739775
	H	-3.559245	-1.743392	-1.894915	-3.525768	-2.086827	-1.493779
	H	-5.366635	-0.448903	-0.781450	-5.305938	-0.499293	-0.807346
**TS23**	C	1.600254	-1.926826	-0.292116	2.209331	-0.489789	-0.561968
	C	2.315314	-0.943368	-0.891984	1.938529	0.810183	-0.829768
	N	1.789489	0.260807	-0.458414	0.846514	1.158457	-0.064273
	N	0.652241	-1.309070	0.500575	1.271665	-0.906270	0.357266
	C	0.761115	0.041666	0.399762	0.431161	0.102977	0.661132
	H	-0.599926	0.701157	2.194861	-0.669111	-0.762786	2.553183
	B	-0.229238	1.072845	1.106592	-0.860222	-0.069496	1.585301
	H	-1.348118	1.023545	0.350349	-1.640362	-0.786407	0.741751
	C	-2.694010	1.249394	-0.488029	-2.502968	-1.167197	-0.528078

	H	-3.233357	1.846719	0.238029	-2.524242	-2.247153	-0.444539
	H	-2.276252	1.791802	-1.328480	-3.421014	-0.644789	-0.288438
	C	2.269580	1.562136	-0.934428	0.159029	2.443931	-0.161504
	H	1.912519	2.338933	-0.267502	-0.175644	2.753941	0.823223
	H	3.359003	1.553520	-0.945067	0.858371	3.172288	-0.566324
	H	1.898580	1.745480	-1.944321	-0.697355	2.342993	-0.829176
	C	-0.351448	-2.019206	1.295408	1.167354	-2.250388	0.913230
	H	-1.338206	-1.908346	0.844032	0.157335	-2.629266	0.758912
	H	-0.083303	-3.073359	1.323047	1.879599	-2.890749	0.398576
	H	-0.368783	-1.620815	2.308369	1.389339	-2.232759	1.979612
	H	1.687630	-2.997592	-0.351350	2.975889	-1.146105	-0.936162
	H	3.147064	-0.992239	-1.573099	2.418126	1.511017	-1.490980
	F	0.233205	2.422829	1.097518	-1.521252	1.152884	1.845648
	C	-3.207219	-0.027578	-0.756327	-1.741620	-0.635106	-1.587280
	N	-3.566161	-1.122566	-0.935476	-1.050100	-0.160239	-2.388930
**TS24**	C	-2.573645	-1.566011	-1.313428	-2.430860	-1.564299	-1.173477
	H	-3.143443	-1.637740	-2.223529	-2.958860	-1.796460	-2.082319
	C	-2.631942	-2.289624	-0.169244	-2.580408	-2.030739	0.089090
	H	-3.255443	-3.122087	0.107460	-3.262897	-2.754789	0.499887
	N	-1.681005	-1.766848	0.688303	-1.633161	-1.395860	0.863390
	N	-1.585457	-0.616119	-1.136688	-1.396675	-0.655137	-1.139121
	C	-1.032982	-0.733207	0.096581	-0.912828	-0.543427	0.112507
	B	0.124308	0.211146	0.713898	0.231408	0.480670	0.613231
	C	1.613702	-0.014559	0.182006	1.728741	0.135821	0.193280
	C	1.945576	-0.788006	-0.945045	2.069412	-0.766091	-0.824826
	C	2.680828	0.608553	0.859948	2.784930	0.783197	0.855105
	C	3.265097	-0.929907	-1.378631	3.396586	-1.006262	-1.175224
	H	1.165735	-1.308058	-1.493530	1.287882	-1.309194	-1.350252
	C	4.001341	0.469756	0.437419	4.113025	0.547359	0.515534
	H	2.469348	1.208488	1.739520	2.555656	1.480305	1.655296
	C	4.300395	-0.299669	-0.689283	4.424674	-0.347769	-0.506555
	H	3.484997	-1.539415	-2.249160	3.628386	-1.713254	-1.964278
	H	4.799278	0.960769	0.985142	4.907993	1.060022	1.046404
	H	5.326938	-0.409135	-1.021656	5.458517	-0.534134	-0.774019
	H	-0.231647	1.468673	0.349528	-0.148703	1.605389	-0.001496
	C	-0.497289	3.042280	0.406827	-1.087236	2.894039	-0.228906

	H	0.237263	3.354407	-0.327353	-0.707465	3.247523	-1.180211
	H	-0.219827	3.192971	1.443766	-0.797468	3.452308	0.652631
	C	-1.372275	-2.362747	1.993680	-1.455133	-1.624755	2.297455
	H	-2.304207	-2.677826	2.461139	-1.962669	-0.846550	2.864645
	H	-0.722168	-3.228567	1.858528	-1.877618	-2.598036	2.537006
	H	-0.879557	-1.624329	2.616067	-0.394727	-1.612941	2.534000
	C	-1.273976	0.414884	-2.130932	-0.967751	0.127640	-2.294735
	H	-1.302328	-0.035463	-3.122173	-0.751050	-0.545903	-3.122923
	H	-2.005648	1.221337	-2.072019	-1.763299	0.818920	-2.575194
	H	-0.278877	0.808415	-1.947857	-0.069008	0.681358	-2.035780
	C	-1.841142	3.275694	0.081304	-2.386104	2.352796	-0.230521
	N	-2.965744	3.392283	-0.205231	-3.417529	1.820592	-0.226368
	F	0.040151	0.253555	2.146081	0.071952	0.758874	1.996975
**TS25**	C	2.526812	-0.933652	-0.235874	2.402174	-0.567608	0.493373
	H	3.346303	-1.607067	-0.055319	3.468835	-0.637152	0.368531
	C	2.158044	-0.266182	-1.358462	1.650757	-0.268191	1.580860
	H	2.595453	-0.246600	-2.341262	1.934136	-0.020637	2.589227
	N	1.025325	0.456989	-1.042051	0.335779	-0.315931	1.180261
	C	0.303369	1.311046	-1.987395	-0.806992	0.008940	2.027964
	H	-0.767458	1.145488	-1.887419	-1.494892	0.645861	1.472836
	H	0.526933	2.361175	-1.800969	-1.322916	-0.899573	2.335720
	H	0.613590	1.044411	-2.995511	-0.442545	0.546622	2.900108
	N	1.616054	-0.601517	0.747724	1.526596	-0.792585	-0.545380
	C	1.653065	-1.132768	2.112731	1.922627	-1.119044	-1.913100
	H	2.577828	-1.692811	2.232582	2.999218	-1.269747	-1.926358
	H	1.628139	-0.315962	2.831260	1.420248	-2.029416	-2.232871
	H	0.804899	-1.794916	2.284109	1.659168	-0.299577	-2.580101
	C	0.687685	0.258562	0.257473	0.256018	-0.637734	-0.126108
	H	-0.533045	0.567739	2.216656	-0.853573	-1.082625	-2.138683
	B	-0.566700	0.818811	1.044927	-1.047800	-0.690251	-1.026260
	C	-0.923449	2.318223	0.787283	-2.274520	-1.383252	-0.335646
	N	-1.228925	3.424986	0.620948	-3.195336	-1.871857	0.162203
	H	-1.620051	0.120682	0.593443	-1.374173	0.596143	-1.125698
	C	-2.950522	-0.655733	0.187444	-1.488145	2.176040	-1.105834
	H	-3.408176	-0.692489	1.169240	-1.489379	2.359401	-2.173106
	H	-3.359249	0.075487	-0.500508	-2.432899	2.291996	-0.588244

	C	-2.541118	-1.880380	-0.362939	-0.328453	2.558876	-0.400918
	N	-2.134593	-2.876727	-0.808407	0.642528	2.786345	0.189544
**TS26**	C	-2.185763	-0.143149	-0.330068	-2.017861	0.411506	0.040803
	C	-1.770675	0.121168	0.943266	-1.467878	-0.541033	0.842116
	N	-0.435919	0.504265	0.852119	-0.101164	-0.320030	0.832773
	C	0.390705	0.858496	2.005159	0.857664	-1.166019	1.531899
	H	1.438702	0.782144	1.732948	1.837612	-1.051095	1.075846
	H	0.178001	1.877953	2.331230	0.914653	-0.886392	2.584648
	H	0.187094	0.166525	2.820802	0.542212	-2.203755	1.438641
	N	-1.094128	0.095095	-1.161911	-0.966247	1.185730	-0.428723
	C	-1.135745	-0.094258	-2.611566	-1.144153	2.302135	-1.349314
	H	-2.007175	0.415492	-3.021536	-1.997399	2.897126	-1.027959
	H	-0.236013	0.324644	-3.049033	-0.250387	2.917135	-1.336718
	H	-1.192856	-1.157665	-2.849935	-1.319513	1.928917	-2.358957
	C	-0.019641	0.496308	-0.438196	0.208186	0.732881	0.051342
	H	1.541420	0.755909	-2.172426	1.717630	2.142299	-1.089790
	B	1.437063	0.819773	-0.980483	1.671038	1.217007	-0.336058
	C	2.086293	2.131746	-0.430981	2.634163	1.388554	0.892184
	N	2.607265	3.092543	-0.040860	3.365834	1.495011	1.780094
	H	2.228922	-0.178234	-0.544141	2.137597	0.162119	-1.009766
	C	3.254325	-1.321547	-0.166034	2.412434	-1.180195	-1.791938
	H	3.745293	-1.411068	-1.128326	2.634565	-0.760260	-2.765347
	H	3.812458	-0.797060	0.601403	3.252944	-1.561235	-1.223719
	C	2.493387	-2.424544	0.253560	1.201534	-1.894847	-1.679512
	N	1.804281	-3.301287	0.589936	0.178203	-2.420673	-1.537309
	C	-2.501113	0.047400	2.240447	-2.085508	-1.662930	1.599915
	H	-2.070336	-0.707685	2.904766	-1.774553	-2.627030	1.189122
	H	-2.481715	1.005689	2.767135	-1.800888	-1.632570	2.654093
	H	-3.543681	-0.217910	2.069015	-3.170808	-1.601500	1.538611
	C	-3.508117	-0.595728	-0.848119	-3.432939	0.675642	-0.338836
	H	-3.950980	0.140920	-1.525039	-3.774242	1.643812	0.035651
	H	-3.425011	-1.540865	-1.392196	-3.557123	0.667924	-1.424012
	H	-4.201885	-0.747314	-0.022048	-4.077724	-0.094969	0.080145
**TS27**	C	-3.206362	0.449623	-1.390844	-3.052004	1.200481	-1.252162
	H	-3.754815	1.034521	-2.108260	-3.442559	2.026752	-1.820579
	C	-3.502546	-0.731547	-0.794398	-3.570073	-0.023495	-0.987382

	H	-4.358746	-1.375720	-0.893549	-4.500576	-0.477241	-1.281748
	N	-2.439705	-1.040814	0.031938	-2.640696	-0.691098	-0.219810
	N	-1.968411	0.839379	-0.916642	-1.820774	1.250898	-0.637128
	C	-1.495522	-0.070201	-0.031979	-1.570471	0.093625	0.002690
	B	-0.093211	-0.040965	0.759984	-0.227962	-0.262243	0.794113
	C	-0.274954	-0.423326	2.274649	-0.481040	-1.155844	2.067237
	N	-0.383739	-0.643557	3.408084	-0.630289	-1.770522	3.033579
	C	1.135930	-0.749140	0.020585	0.993874	-0.723174	-0.122338
	C	1.137934	-0.986081	-1.367158	0.978302	-0.595461	-1.518178
	C	2.298483	-1.114449	0.727900	2.176381	-1.197994	0.466663
	C	2.236008	-1.551685	-2.015657	2.090884	-0.918796	-2.292579
	H	0.264744	-0.730735	-1.960031	0.081257	-0.240676	-2.019841
	C	3.399746	-1.679340	0.088039	3.291564	-1.521744	-0.297972
	H	2.341376	-0.957723	1.801288	2.222937	-1.316812	1.546008
	C	3.374735	-1.900486	-1.290259	3.253606	-1.380166	-1.684007
	H	2.201276	-1.721622	-3.086725	2.049015	-0.810041	-3.370686
	H	4.278235	-1.949868	0.664593	4.192200	-1.884509	0.184822
	H	4.230176	-2.340791	-1.790567	4.121876	-1.631004	-2.282456
	H	0.282042	1.261342	0.910615	0.208529	0.861191	1.358307
	C	0.871816	2.618020	1.457285	1.175044	1.879397	2.105275
	H	1.501321	2.187417	2.228133	1.602540	1.176828	2.811263
	H	-0.043748	3.080412	1.810084	0.459423	2.591469	2.499061
	C	-2.361405	-2.264508	0.836529	-2.786305	-2.069567	0.247113
	H	-1.345000	-2.651162	0.815104	-1.844491	-2.596333	0.104728
	H	-2.652963	-2.062332	1.866624	-3.058187	-2.083773	1.301327
	H	-3.034144	-3.001373	0.402698	-3.563576	-2.546974	-0.344731
	C	-1.304934	2.083728	-1.316609	-0.919699	2.401868	-0.665601
	H	-1.534493	2.879095	-0.607538	-0.983683	2.947930	0.275190
	H	-0.230766	1.930449	-1.365013	0.101955	2.061062	-0.828402
	H	-1.668638	2.363838	-2.303257	-1.220359	3.048755	-1.486325
	C	1.537255	3.276344	0.410511	2.023711	2.334315	1.077296
	N	2.065151	3.776044	-0.499904	2.676168	2.674140	0.181523
**TS28**	C	0.312023	0.305402	0.268632	0.216446	0.360848	0.017802
	C	0.325926	1.436869	-1.671932	-0.019637	-0.672696	-1.948571
	H	-0.076252	1.987601	-2.503485	-0.537869	-1.098460	-2.789898
	N	-0.458474	0.962273	-0.638682	-0.675402	-0.085962	-0.890390

	N	1.582549	0.366665	-0.213189	1.434228	0.036812	-0.464011
	C	-1.930403	1.150185	-0.559295	-2.144822	0.005646	-0.768259
	H	-2.249994	0.540307	0.283187	-2.332255	0.284850	0.268321
	C	2.802765	-0.190649	0.426739	2.714492	0.357792	0.207145
	H	2.447336	-0.681990	1.328846	2.525025	0.221458	1.271501
	H	0.709797	-0.960033	2.206922	0.847663	1.308891	2.078722
	B	-0.175057	-0.448255	1.582592	-0.111824	1.029343	1.425036
	C	1.601057	1.062892	-1.407279	1.306383	-0.595586	-1.680708
	H	2.503485	1.234339	-1.966711	2.150598	-0.943940	-2.248030
	C	3.451865	-1.236038	-0.482708	3.806441	-0.611642	-0.221414
	H	2.742943	-2.025577	-0.740128	3.491103	-1.648448	-0.092690
	H	3.828122	-0.791369	-1.407660	4.102991	-0.451963	-1.260438
	H	4.297868	-1.690357	0.037521	4.684546	-0.438438	0.401697
	C	3.756848	0.938569	0.823312	3.083912	1.810079	-0.070590
	H	3.259848	1.658397	1.477115	2.294927	2.483218	0.269337
	H	4.609328	0.518537	1.361576	4.007116	2.061785	0.454224
	H	4.141303	1.470125	-0.051030	3.240275	1.960972	-1.141738
	C	-2.265401	2.614237	-0.265746	-2.674642	1.097606	-1.689626
	H	-1.950914	3.266175	-1.084999	-2.465280	0.851727	-2.733888
	H	-3.345381	2.722217	-0.144195	-3.755514	1.186017	-1.567302
	H	-1.782583	2.949253	0.653907	-2.217814	2.058760	-1.450427
	C	-2.608098	0.624080	-1.826661	-2.779568	-1.354364	-1.036197
	H	-3.691474	0.674940	-1.699453	-3.842508	-1.303309	-0.795826
	H	-2.348725	1.222080	-2.703913	-2.689051	-1.637911	-2.087229
	H	-2.332985	-0.414783	-2.018714	-2.315916	-2.130351	-0.424280
	C	-1.121380	0.360775	2.526810	-1.145855	2.206517	1.346208
	N	-1.832871	0.917870	3.254940	-1.922213	3.061594	1.306766
	H	-0.906405	-1.500227	1.165118	-0.727132	0.028387	2.066494
	C	-1.721739	-2.809371	0.843465	-1.228410	-1.388098	2.512742
	H	-2.724915	-2.422518	0.983894	-2.285174	-1.322299	2.277846
	H	-1.339880	-3.429655	1.646394	-0.963621	-1.350824	3.562474
	C	-1.375301	-3.193785	-0.462278	-0.459246	-2.249081	1.700491
	N	-1.046092	-3.456120	-1.548140	0.205395	-2.879439	0.990750
**TS29**	C	-2.117144	-0.925917	1.384410	-1.936539	-0.193103	1.485759
	H	-2.473953	-1.313621	2.322537	-2.383469	-0.746508	2.293533
	C	-2.614979	-1.042106	0.127722	-2.487890	0.604350	0.540673

	H	-3.489245	-1.551294	-0.238065	-3.510331	0.889405	0.360934
	N	-1.762440	-0.352631	-0.712951	-1.457395	1.052830	-0.257136
	N	-0.966014	-0.170117	1.290995	-0.582938	-0.215257	1.245945
	C	-0.748472	0.190112	0.002924	-0.286705	0.551411	0.177069
	B	0.506135	1.015035	-0.547546	1.180792	0.660139	-0.458661
	H	1.588024	0.240809	-0.269976	1.414933	-0.631126	-0.794437
	C	2.931797	-0.557102	-0.324121	1.369648	-2.037377	-1.423656
	H	3.253334	-0.259461	-1.316092	1.547938	-1.781410	-2.461485
	H	3.449667	-0.078221	0.499881	2.184794	-2.527914	-0.903747
	C	-1.968804	-0.242685	-2.163792	-1.669168	1.963004	-1.384020
	H	-2.885163	-0.774513	-2.409464	-0.721857	2.144702	-1.877683
	H	-2.064570	0.802315	-2.449999	-2.369762	1.504448	-2.080075
	H	-1.132759	-0.689870	-2.696622	-2.079820	2.899701	-1.009704
	C	-0.122948	0.193343	2.432931	0.381020	-0.986407	2.027027
	H	-0.320071	1.220351	2.739642	0.876851	-0.345124	2.754823
	H	-0.348164	-0.484000	3.253676	-0.154143	-1.786041	2.533762
	H	0.925960	0.088391	2.163153	1.125187	-1.419261	1.360391
	C	0.783348	2.367000	0.220519	2.287682	1.020184	0.616748
	N	1.038099	3.342948	0.790432	3.107042	1.223772	1.403166
	C	2.609273	-1.914542	-0.154761	0.068414	-2.488062	-1.108745
	N	2.273235	-3.019776	-0.008373	-1.011620	-2.776674	-0.803997
	F	0.485964	1.190261	-1.948518	1.269037	1.433527	-1.620443
**TS30**	C	-2.296041	-1.513021	0.454844	-2.367700	0.253335	1.052615
	H	-2.715492	-2.416985	0.860277	-3.264381	-0.213552	1.421725
	C	-2.778190	-0.646621	-0.470251	-2.139971	1.507863	0.594365
	H	-3.699144	-0.651429	-1.026255	-2.798390	2.352487	0.489509
	N	-1.833760	0.348133	-0.626978	-0.811704	1.570997	0.238815
	N	-1.062805	-1.036882	0.845795	-1.174951	-0.422651	0.965754
	C	-0.773578	0.112862	0.185330	-0.218160	0.384610	0.467214
	B	0.591059	0.925952	0.344665	1.265542	-0.098718	0.146877
	C	0.794253	2.200700	-0.539527	2.239211	0.945169	-0.498361
	N	1.034189	3.124966	-1.193748	2.977716	1.662645	-1.016689
	H	1.551991	0.101324	-0.120215	1.078348	-0.991275	-0.819429
	C	2.793636	-0.671931	-0.730689	0.608396	-1.799039	-2.091667
	H	3.140421	0.119787	-1.385291	1.264442	-1.338132	-2.820475
	H	3.358560	-0.816569	0.183587	0.845931	-2.810723	-1.784138

	C	-2.009378	1.475291	-1.552025	-0.177419	2.768882	-0.314859
	H	-1.907956	2.417818	-1.019164	0.680948	3.047818	0.291776
	H	-1.277440	1.425246	-2.355954	0.137651	2.584003	-1.339879
	H	-3.010253	1.406624	-1.971562	-0.912787	3.569225	-0.297160
	C	-0.222128	-1.695583	1.850357	-0.998790	-1.827989	1.324681
	H	-0.344297	-1.215691	2.820953	-0.545815	-1.912439	2.311492
	H	-0.524313	-2.738425	1.914807	-1.976796	-2.302979	1.317885
	H	0.821289	-1.647059	1.549298	-0.360516	-2.313876	0.588487
	C	0.994635	1.208631	1.834227	1.936998	-0.877274	1.333605
	N	1.320365	1.404635	2.927898	2.418153	-1.478731	2.191745
	C	2.289097	-1.826578	-1.351906	-0.758792	-1.456659	-2.160973
	N	1.813083	-2.766124	-1.846676	-1.869070	-1.128025	-2.141611
